# Natural Biomaterials for Osteochondral Repair: From Source to Strategy

**DOI:** 10.1002/adhm.71344

**Published:** 2026-06-16

**Authors:** Hengyu Liu, Hanyang Zhang, Wenbo Yang, Hao Chen, Jincheng Wang, Fei Chang

**Affiliations:** ^1^ Department of Orthopedic Surgery The Second Hospital of Jilin University Changchun P. R. China; ^2^ Jilin Provincial Institute of Orthopedics Changchun P. R. China

**Keywords:** biological origin, decellularized extracellular matrix, natural biomaterials, osteochondral repair

## Abstract

Osteochondral tissue repair remains a formidable clinical challenge due to its complex layered structure and limited intrinsic healing capacity. Compared with synthetic materials, natural biomaterials offer distinct advantages, including excellent biocompatibility, intrinsic bioactivity, and structural similarity to native tissues, making them particularly well‐suited for recreating the native tissue microenvironment and guiding cellular behavior. This review systematically examines natural biomaterials used in osteochondral repair according to their biological origin, covering human‐derived, animal‐derived, plant‐derived, and microbial‐derived categories, and discusses how the source‐specific biological properties of each category underpin their respective repair mechanisms and applications. Within animal‐derived materials, particular attention is given to decellularization strategies, as the choice of method critically determines the preservation of bioactive components and the immunogenic profile of the resulting scaffold. Notably, 91.5% of the 47 osteochondral repair products approved globally since 2000 incorporate natural materials, further underscoring their enduring clinical relevance. We further highlight that the absence of standardized preparation, quality control, and evaluation frameworks remains an important challenge for broader clinical adoption. Establishing reproducible and verifiable standards will be essential to bridging the gap between laboratory innovation and clinical translation.

## Graphical abstract

Biological origin‐guided overview of natural biomaterials and therapeutic strategies for osteochondral tissue engineering. The circular diagram categorizes representative materials and strategies into plant/algae‐derived, microbial‐derived, animal‐derived, and human‐derived sources, centered on an osteochondral defect repair model. The outer arrows highlight their integrated and synergistic contributions to cartilage regeneration and subchondral bone repair.

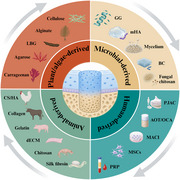

## 1 Introduction

Osteochondral tissue exhibits a complex stratified architecture comprising three distinct yet integrated zones: the superficial articular cartilage, the intermediate cartilage‐bone interface represented by the calcified cartilage layer, and the underlying subchondral bone. These hierarchical regions collectively constitute a functionally integrated osteochondral unit [1]. From the cartilage surface to subchondral bone, the osteochondral unit exhibits gradient transitions across multiple biological parameters, including cell phenotypes ranging from chondrocytes to hypertrophic chondrocytes and osteoblasts, extracellular matrix (ECM) components such as type II collagen and proteoglycans, vascularization density, and mechanical properties. However, it is precisely this intricate stratified architecture, coupled with the tissue's inherently limited self‐healing capacity, that renders osteochondral injury repair a formidable clinical challenge [2].

Current clinical treatments encompass palliative therapies (non‐steroidal anti‐inflammatory drugs, intra‐articular hyaluronic acid injections), reparative therapies (microfracture, bone marrow stimulation techniques), and regenerative therapies (autologous chondrocyte implantation (ACI), osteochondral autograft transplantation (OATS) [3]. However, these approaches remain limited by several constraints: palliative therapies merely alleviate symptoms without achieving tissue regeneration; microfracture frequently yields fibrocartilage with inferior mechanical properties; autologous chondrocyte implantation requires secondary surgery and is associated with donor site morbidity and chondrocyte phenotype loss [4]. More critically, most existing treatments focus exclusively on either cartilage or bone repair while neglecting cartilage‐bone interface reconstruction, resulting in suboptimal long‐term outcomes [5].

To address these challenges, osteochondral tissue engineering has emerged as a more promising therapeutic avenue [6]. Ideal scaffold materials must simultaneously fulfill multiple requirements, including biocompatibility, mechanical compatibility, biodegradability, gradient architecture, and bioactivity. Over the past decades, researchers have developed synthetic polymers, bioceramics, and natural biomaterials [7]. While synthetic materials offer advantages such as controllable mechanical properties and facile scalability for mass production, they lack the intrinsic biological signaling molecules of native ECM, failing to effectively modulate cellular behaviors. Moreover, their degradation products may induce local acidic environments and inflammatory responses [8].

In contrast, natural biomaterials demonstrate unique advantages in osteochondral repair owing to their superior biocompatibility, intrinsic bioactivity, and structural similarity to native tissues [9]. The native ECM comprises a complex repertoire of components, including collagen, laminin, fibronectin, proteoglycans, glycosaminoglycans (GAGs), and various growth factors. These inherent mechanical and biochemical properties regulate cellular functions such as adhesion, proliferation, migration, and differentiation, while actively participating in cell signaling pathways through integrin binding and signaling molecule modulation [10].

Our analysis of 47 globally approved osteochondral repair products since 2000 reveals that 43 products (91.5%) are based on natural biomaterials, encompassing various categories including decellularized matrices, collagen scaffolds, hyaluronic acid derivatives, and chondrocyte‐scaffold constructs, thereby confirming the clinical value of natural biomaterials in osteochondral regeneration. However, the sources of natural biomaterials are extremely diverse, ranging from human‐derived materials (autologous and allogeneic tissues) to xenogeneic animal‐derived materials (decellularized matrices, collagen, and chitosan from porcine, bovine, and marine species), plant/algae‐derived materials (alginate, cellulose), and microbial‐derived materials (bacterial cellulose, hyaluronic acid, gellan gum). Materials from different sources exhibit significant differences in compositional components, structural characteristics, mechanical properties, and biological functions [11]. More importantly, natural biomaterials also face safety challenges including endotoxin contamination, residual DNA/RNA fragments, pathogen transmission risks, and immunogenicity. This raises a fundamental scientific question: when natural biomaterials vary in their sources and preparation conditions, how can we ensure their performance consistency, biosafety, and reproducibility in osteochondral repair?

To our knowledge, although numerous studies have reported the application of various natural materials in osteochondral repair, comprehensive reviews systematically analyzing the correlations between material sources and their compositional characteristics, biological functions, and repair outcomes remain scarce. Currently, many studies primarily select natural biomaterials based on availability, cost, and processing convenience, while paying insufficient attention to the intrinsic relationships between material sources and their osteochondral repair efficacy [9]. For instance, type I collagen, predominantly found in bone tissue, provides high‐strength mechanical support, while type II collagen, as the major component of cartilage, is more suitable for cartilage layer repair [12]. Similarly, although decellularized matrices from different sources [13], decellularized cartilage matrix (DCM), decellularized bone matrix (DBM)) all undergo decellularization processing, they exhibit significant differences in retained ECM components, growth factor content, mechanical properties, residual DNA content (<50 ng/mg dry weight safety threshold), and endotoxin levels (<20 EU/device FDA limit) [14]. Furthermore, the translation of natural biomaterials from laboratory research to clinical application faces critical challenges, including endotoxin removal, DNA clearance validation, batch‐to‐batch consistency, and quality control [15].

**FIGURE 1:**
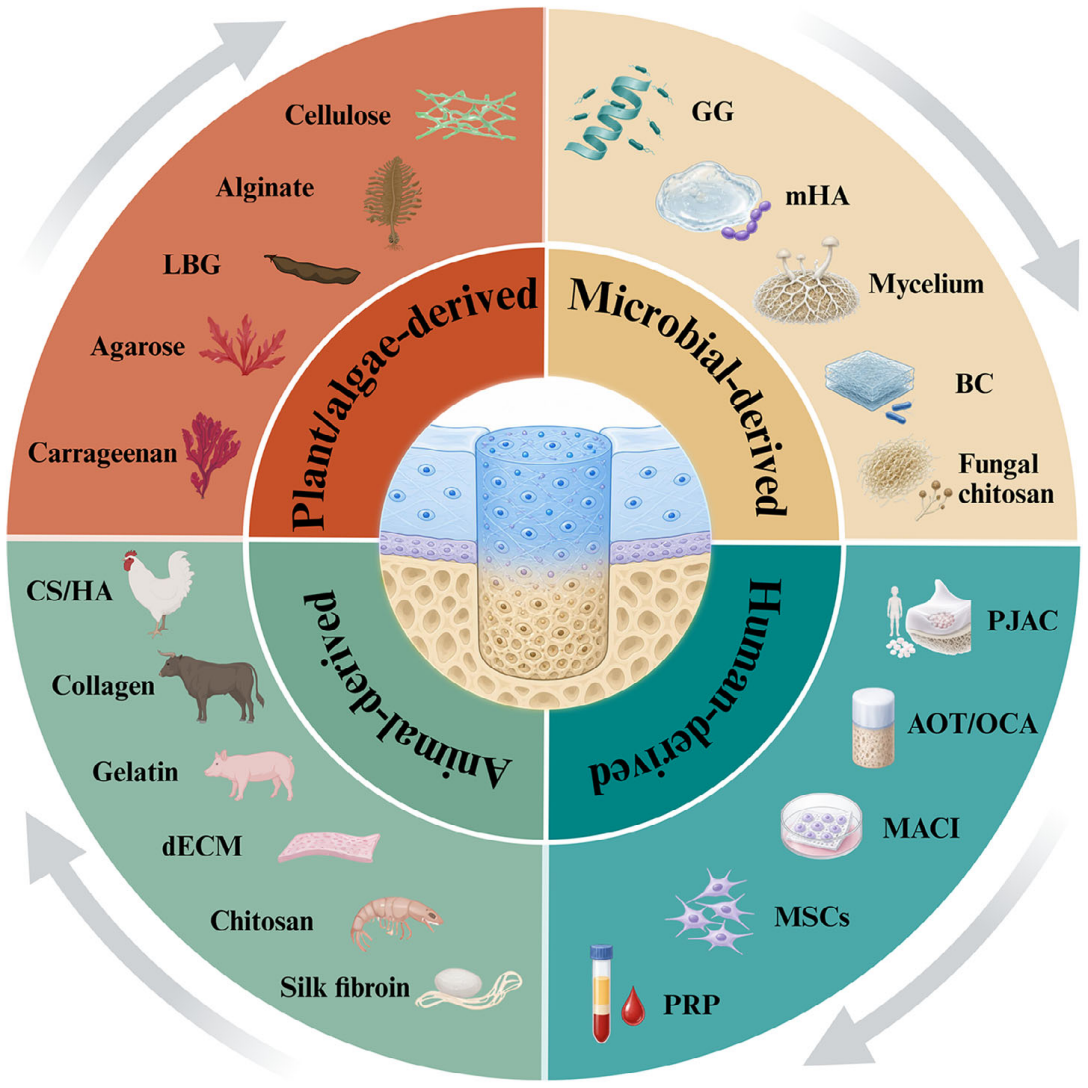
Biological origin‐guided overview of natural biomaterials and therapeutic strategies for osteochondral tissue engineering. The central panel illustrates an osteochondral defect repair model, highlighting the role of natural biomaterials and biologically derived therapeutic strategies in osteochondral tissue engineering, particularly in cartilage regeneration and subchondral bone repair. The surrounding circular diagram categorizes representative materials and therapeutic strategies according to their biological origins, including plant/algae‐derived, microbial‐derived, animal‐derived, and human‐derived sources. The outer arrows emphasize the integrated contribution of these biologically derived materials and strategies, which may act individually or synergistically to promote osteochondral repair. These materials and strategies encompass polysaccharide‐based biomaterials, extracellular matrix‐derived and protein‐based biomaterials, cell‐based therapies, platelet‐derived products, and osteochondral grafting approaches. This figure was created by the authors. The abbreviations of materials and related therapeutic strategies are provided for quick reference. CS/HA: chondroitin sulfate/hyaluronic acid; dECM: decellularized extracellular matrix; LBG: locust bean gum; GG: gellan gum; mHA: microbial hyaluronic acid; BC: bacterial cellulose; PJAC: particulated juvenile articular cartilage; AOT/OCA: autologous osteochondral transplantation/osteochondral allograft transplantation; MACI: matrix‐induced autologous chondrocyte implantation; MSCs: mesenchymal stem cells; PRP: platelet‐rich plasma.

Therefore, this review focuses on systematically analyzing the differential performance of human‐derived, xenogeneic animal‐derived, plant/algal‐derived, and microbial‐derived materials in osteochondral repair from a “biological origin‐repair outcome” perspective, elucidating the relationships among compositional characteristics, biological functions, and repair efficacy of materials from different origins. Building upon these correlation analyses, we further identify that the core bottleneck hindering clinical translation of natural materials lies in the absence of standardized “fabrication‐quality control‐evaluation” frameworks. Only through establishing reproducible and verifiable fabrication and evaluation standards can natural biomaterials truly bridge the gap from laboratory research to clinical implementation in the field of osteochondral repair. An overview of the biological origins of natural biomaterials and their corresponding therapeutic strategies discussed in this review is illustrated in Figure 1.

## 2 Multiscale Characteristics of Osteochondral Tissue and Repair Challenges

### 2.1 Mechanical Gradients in Stratified Architecture

**FIGURE 2:**
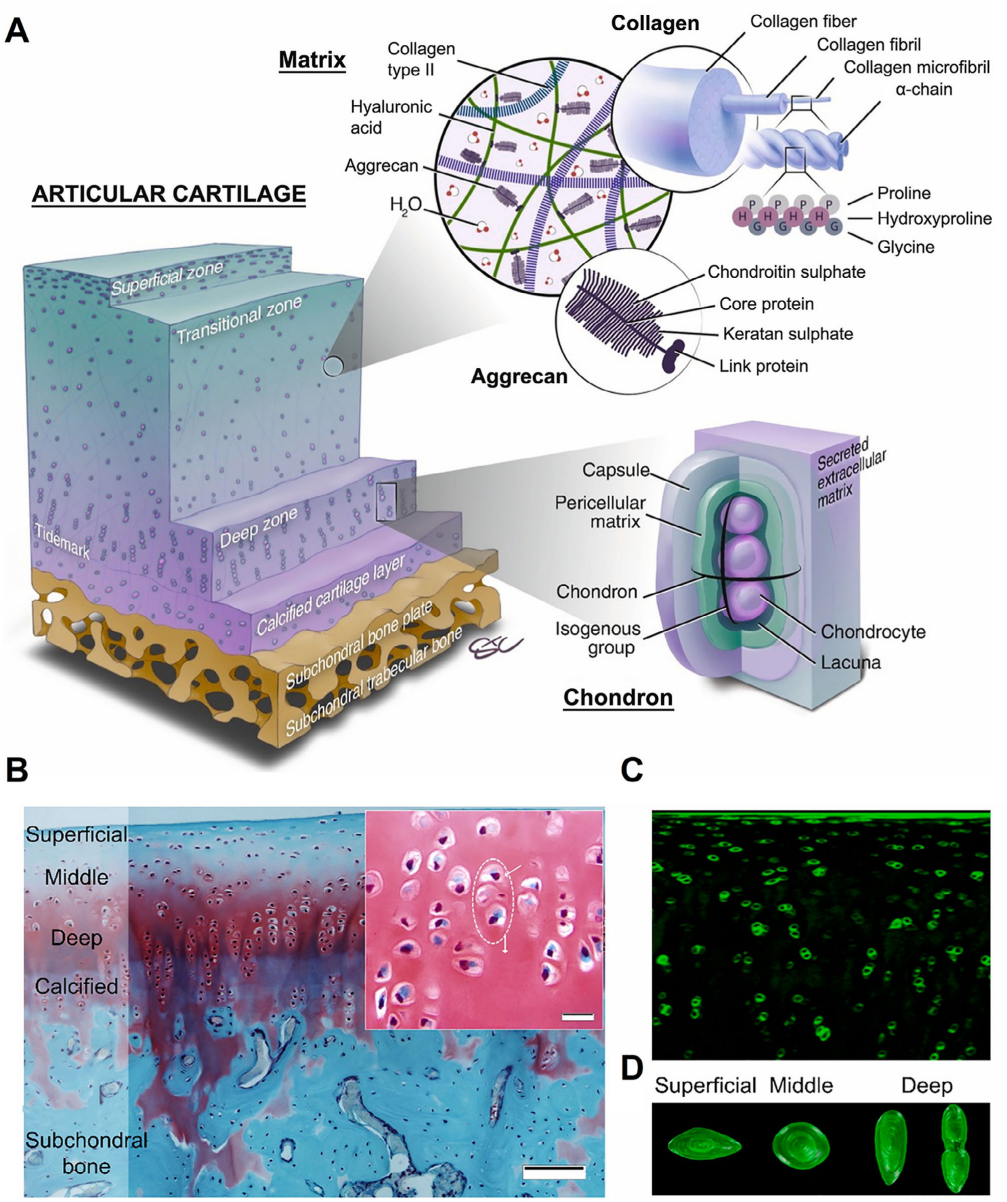
Schematic illustration of the osteochondral unit and its zonal structure. (A) Schematic diagram of the extracellular structure of the osteochondral unit, showing the main components, including collagen, aggrecan, and proteoglycans in the cartilage matrix. Reproduced with permission [29]. Copyright 2019, Springer. (B) Safranin O/Fast Green staining showing the zonal architecture of healthy osteochondral tissue in rabbit knee joints. Reproduced with permission [30]. Copyright 2018, Elsevier. (C) Fluorescence confocal microscopy image of osteochondral tissue with type VI collagen immunolabeling. (D) Typical morphology of chondrocytes under compression in different zones. Significant changes in chondrocyte height, shape, and volume were observed. Reproduced with permission [31]. Copyright 2007, Elsevier.

Osteochondral tissue is a highly specialized composite structure that exhibits a continuous stratified architecture from the articular surface to the subchondral bone, with each layer possessing distinct compositional, structural, and functional characteristics (Figure 2). This elaborate stratified design enables the osteochondral unit to simultaneously fulfill the lubrication requirements for joint motion, mechanical demands for load transmission, and buffering functions for stress dissipation [16]. Figure 2A illustrates a schematic representation of the extracellular architecture of the osteochondral unit and its principal components, including collagen and aggrecan proteoglycans within the matrix. Figure 2B displays the zonal histological organization of articular cartilage through Safranin O/Fast Green staining of rabbit knee joint tissue sections, clearly revealing distinct regions of the cartilage ECM. Figure 2C presents confocal fluorescence images of osteochondral tissue blocks immunolabeled for type VI collagen. Figure 2D demonstrates typical morphological changes of chondrocytes across different zones under compression, with chondrocyte height, shape, and volume undergoing substantial alterations. Understanding this stratified architecture and its mechanical gradients is crucial for designing effective osteochondral repair materials.

Articular cartilage itself can be further subdivided into four functional zones: the superficial zone, middle zone, deep zone, and calcified cartilage layer (Table 1) [17]. The superficial zone, located at the articular surface, accounts for approximately 10‐20% of total cartilage thickness and is characterized by flattened chondrocytes aligned parallel to the articular surface, with collagen fibers (predominantly type II collagen) also distributed parallel to the surface, forming a dense fibrous network (Figure 2B,D). This unique arrangement confers the superficial zone with the highest tensile strength and lowest permeability, enabling it to resist shear forces and maintain synovial fluid lubrication. Its compressive modulus ranges from approximately 0.1–0.5 MPa, making it the softest region within cartilage [18]. The middle zone comprises 40%–60% of cartilage thickness, representing the largest volumetric region, where chondrocytes exhibit round or oval morphologies with random distribution, collagen fiber orientation becomes increasingly random, and proteoglycan content significantly increases (Figure 2A,B). The primary function of the middle zone is to provide compressive resistance, with a compressive modulus of approximately 0.5–0.9 MPa. The highly negative charge of proteoglycans enables them to attract and retain substantial water content, forming a highly hydrated gel‐like matrix that serves as the key source of cartilage compressive resistance [19]. The deep zone accounts for approximately 30% of cartilage thickness and is characterized by columnar chondrocytes arranged perpendicular to the articular surface, with collagen fibers also exhibiting radial vertical alignment, anchoring into the underlying calcified cartilage layer (Figure 2B,D). The deep zone exhibits the highest proteoglycan content, with a compressive modulus of approximately 0.9–2.0 MPa, making it the hardest non‐calcified region within cartilage. This vertically aligned fibrous architecture effectively transmits compressive loads to the underlying subchondral bone while preventing cartilage delamination from the bone surface [20].

**TABLE 1:** Zonal organization and composition of osteochondral unit.

Layer	Function	Characteristics	Primary Collagen	Reference
Superficial layer	Resist shear stress, boundary lubrication, smooth gliding	Flattened chondrocytes parallel to surface, high PRG4/Lubricin secretion, highest water content (∼80%), lowest proteoglycan	Type II (parallel), Type IX, XI	[18]
Middle layer	Resist compression, shock absorption	Spherical chondrocytes, random collagen orientation, moderate water and proteoglycan content	Type II (random), Type IX, XI	[32]
Deep layer	Maximum compressive resistance, anchor cartilage to bone	Columnar chondrocytes perpendicular to surface, highest proteoglycan content, lowest water content (∼65%)	Type II (perpendicular), Type IX, X, XI	[33]
Calcified cartilage	Mechanical transition between cartilage and bone, anchor collagen fibers	Hypertrophic chondrocytes, mineralized matrix, separated from deep zone by tidemark	Type II, X, Type IX	[34]
Subchondral Bone	Mechanical support, load distribution, nutrient supply	Dense cortical plate, high mineralization, vascular network, active bone remodeling	Type I, Type III, V	[35]

The calcified cartilage layer serves as a critical transitional zone connecting articular cartilage and subchondral bone, with a thickness of approximately 50–200 µm. It is characterized by the presence of hypertrophic chondrocytes and mineralized ECM, where hydroxyapatite crystals begin to deposit on collagen fibers, resulting in dramatic changes in tissue mechanical properties (Figure 2A,C). The compressive modulus of the calcified cartilage layer ranges from approximately 50–100 MPa, intermediate between cartilage and bone, serving as a mechanical buffer and stress transition zone [21]. The interface between the calcified cartilage layer and non‐calcified cartilage is termed the “tidemark,” which represents a distinct boundary marking the transition from non‐mineralized to mineralized cartilage. The tidemark functions not only as a structural demarcation but also as a biological barrier that restricts vascular and neural invasion from subchondral bone into cartilage [22].

Subchondral bone comprises two principal components: the subchondral bone plate and subchondral trabecular bone (Figure 2A). The subchondral bone plate is a layer of dense cortical bone with a thickness of approximately 0.5–2 mm, located directly beneath the calcified cartilage layer. It exhibits a high degree of mineralization and dense architecture, with a compressive modulus of approximately 100–500 MPa, making it the hardest component of the osteochondral unit [23]. The primary functions of the subchondral bone plate are to provide robust mechanical support and serve as the nutritional supply foundation for articular cartilage. The subchondral trabecular bone, situated beneath the bone plate, consists of an interconnected trabecular network characterized by high porosity (approximately 50%–90%) and vascularization, with a compressive modulus of approximately 10–50 MPa, lower than cortical bone but higher than cartilage [24]. The porous architecture of trabecular bone enables it to absorb and dissipate impact forces from the articular surface while providing space for bone marrow and vasculature, supporting bone metabolism and nutrient supply [25]. The orientation of subchondral bone trabeculae typically aligns with the principal loading direction, reflecting the adaptive remodeling of bone tissue in response to the mechanical environment [26].

Osteochondral tissue exhibits a smooth mechanical gradient from the articular cartilage surface to deep subchondral bone, with compressive modulus gradually increasing from approximately 0.1–500 MPa across three orders of magnitude (Figure 2D). This transition is achieved through progressive changes in tissue composition and structure: water content decreases from about 80%–20%, proteoglycan content rises, then falls, collagen content increases from roughly 15%–30%, and mineralization steadily intensifies. Biologically, the low modulus of cartilage allows for significant deformation and effective stress distribution, while the intermediate modulus of the calcified cartilage and subchondral bone plate buffers stress transitions. The porous subchondral trabecular bone further absorbs impact energy via microdamage and remodeling, helping to protect joint integrity [27].

The mechanical gradient of osteochondral tissue imposes critical requirements on tissue engineering scaffold design. An ideal osteochondral scaffold should mimic this native mechanical gradient, providing a low‐modulus, highly elastic environment in the cartilage layer to support chondrocyte phenotype maintenance and ECM secretion, a high‐modulus, high‐strength structure in the bone layer to support osteoblast mineralization and bone formation, and an intermediate‐modulus transition in the interface region to achieve mechanical integration between the two layers [28].

### 2.2 Biological Barriers at the Cartilage‐Bone Interface

The cartilage‐bone interface serves not only as a transitional zone for mechanical properties but also as a complex biological barrier that plays a crucial role in maintaining the avascular nature of articular cartilage, regulating nutrient exchange, preventing abnormal ossification, and coordinating the metabolic balance between cartilage and bone [18]. Disruption of this interface represents one of the core reasons why osteochondral injuries are difficult to self‐repair and constitutes a critical structure that must be reconstructed in tissue engineering repair [36]. Understanding the biological characteristics of the cartilage‐bone interface holds significant importance for developing effective osteochondral repair strategies.

The core structure of the cartilage‐bone interface is the calcified cartilage layer and its upper and lower boundaries. The upper boundary of the calcified cartilage layer is the tidemark, which distinctly separates non‐calcified articular cartilage from calcified cartilage [37]. Histologically, the tidemark appears as a wavy basophilic line composed of highly mineralized matrix and specialized collagen fiber arrangements. Electron microscopy studies reveal that collagen fibers at the tidemark exhibit greater diameter, denser arrangement, and abundant calcium‐phosphate deposition, forming a relatively impermeable barrier [38]. These structural characteristics render the tidemark an important interface that restricts molecular diffusion, with macromolecules (such as growth factors and proteoglycans) unable to readily cross this barrier, thereby maintaining biochemical microenvironmental differences between the deep zone of cartilage and the calcified layer [39]. The lower boundary of the calcified cartilage layer is the interface with the subchondral bone plate, which exhibits a highly interdigitated structure where collagen fibers from the calcified cartilage penetrate into the subchondral bone plate, forming nail‐like anchoring structures that provide robust interfacial bonding strength [40].

One of the most prominent biological characteristics of the cartilage‐bone interface is its stringent control over vascular invasion. Under normal conditions, articular cartilage is a completely avascular tissue, relying on synovial fluid and diffusion from subchondral bone for nutrient supply [41]. The calcified cartilage layer prevents upward vascular growth from subchondral bone into articular cartilage through multiple mechanisms. First, the highly mineralized matrix of the calcified cartilage layer forms a physical barrier that restricts vascular endothelial cell migration and invasion [42]. Second, cells and matrix within the calcified cartilage layer secrete various anti‐angiogenic factors, such as cartilage oligomeric matrix protein (COMP), pigment epithelium‐derived factor (PEDF), and thrombospondin‐1 (TSP‐1), which inhibit vascular endothelial growth factor (VEGF) activity and prevent vascular bud formation [43]. Furthermore, although hypertrophic chondrocytes in the calcified cartilage layer express VEGF, their expression levels are tightly regulated, sufficient only to maintain the vascular network of subchondral bone without causing vessels to breach the calcified layer into cartilage [36]. This precise vascular regulatory mechanism is crucial for maintaining the hypoxic environment of cartilage and the chondrocyte phenotype, as vascular invasion introduces oxygen, inflammatory factors, and immune cells, leading to cartilage degradation and fibrosis [44].

The cartilage‐bone interface also plays a critical role in nutrient exchange and metabolic waste removal. Although the calcified cartilage layer restricts macromolecular diffusion, small molecule nutrients (such as glucose, oxygen, and amino acids) can still diffuse across this interface, supplying upward from the vascular network of subchondral bone to the deep zone of articular cartilage [45]. Studies have demonstrated that the subchondral bone plate contains numerous microchannels (approximately 10–100 µm in diameter) that traverse the bone plate and calcified cartilage layer, reaching directly into the deep zone of non‐calcified cartilage, providing direct nutritional pathways for cartilage [46]. These microchannels contain not only blood vessels but also nerve fibers and undifferentiated mesenchymal stem cells, which play important roles in cartilage nutrient supply, signal transduction, and injury repair [47]. However, under pathological conditions such as osteoarthritis, the number and diameter of these microchannels increase significantly, leading to abnormal vascular and neural invasion into cartilage, disrupting the integrity of the cartilage‐bone interface and accelerating cartilage degradation [44].

Another important function of the cartilage‐bone interface is coordinating the metabolic balance between cartilage and bone. The calcified cartilage layer resides at the frontier of endochondral ossification and represents a critical region for cartilage‐to‐bone transformation [34]. In this region, chondrocytes undergo hypertrophic differentiation, expressing type X collagen, matrix metalloproteinase‐13 (MMP‐13), and alkaline phosphatase (ALP), promoting matrix mineralization and degradation [42]. Concurrently, osteoclasts from subchondral bone invade the calcified cartilage layer, resorbing the mineralized cartilage matrix and creating space for osteoblast‐mediated bone formation [48]. This coupled process of cartilage degradation and bone formation is precisely regulated by multiple signaling pathways, including the parathyroid hormone‐related peptide (PTHrP)/Indian hedgehog (Ihh) signaling axis, transforming growth factor‐β (TGF‐β)/bone morphogenetic protein (BMP) signaling pathways, and the Wnt/β‐catenin signaling pathway [28]. Dysregulation of these signaling pathways leads to abnormal thickening or thinning of the calcified cartilage layer, disrupting the structure and function of the cartilage‐bone interface and consequently affecting the integrity of the entire osteochondral unit [5].

During osteochondral injury and repair, reconstruction of the cartilage‐bone interface represents one of the most challenging tasks. When osteochondral defects occur, the calcified cartilage layer is often destroyed, resulting in direct exposure of subchondral bone vasculature and bone marrow cells to the defect area [49]. Under these circumstances, blood vessels rapidly invade the repair tissue, introducing abundant fibroblasts and inflammatory cells, causing the repair tissue to predominantly consist of fibrocartilage rather than hyaline cartilage, with mechanical properties and biological functions far inferior to native cartilage [50]. Furthermore, lacking the buffering effect of the calcified cartilage layer, the interfacial bonding strength between newly formed cartilage and subchondral bone is weak, prone to delamination and detachment [51]. Therefore, an ideal osteochondral tissue engineering strategy should not only repair the cartilage and bone layers but also reconstruct a cartilage‐bone interface with anti‐vascular invasion capability, nutrient exchange function, and mechanical transition properties [52].

In recent years, researchers have begun exploring multiple strategies to reconstruct the cartilage‐bone interface. One strategy involves introducing gradient mineralization structures in the interface region of scaffolds to mimic the mineralization gradient of the calcified cartilage layer, while loading anti‐angiogenic factors (such as TSP‐1 and endostatin) to inhibit vascular invasion [53]. Another strategy utilizes a stratified scaffold design, inserting an intermediate layer containing hypertrophic chondrocytes or pre‐differentiated mesenchymal stem cells between the cartilage and bone layers to induce formation of calcified cartilage‐like structures [54]. Other studies have attempted to use decellularized calcified cartilage matrix as an interface material, preserving the compositional and structural characteristics of native calcified cartilage layer to provide a natural biological template for interface reconstruction [55]. However, these strategies remain in early research stages, and achieving stable, functional cartilage‐bone interface reconstruction in the in vivo environment continues to represent a major challenge in the field of osteochondral tissue engineering [56].

### 2.3 Limitations of Current Treatment Methods

**FIGURE 3:**
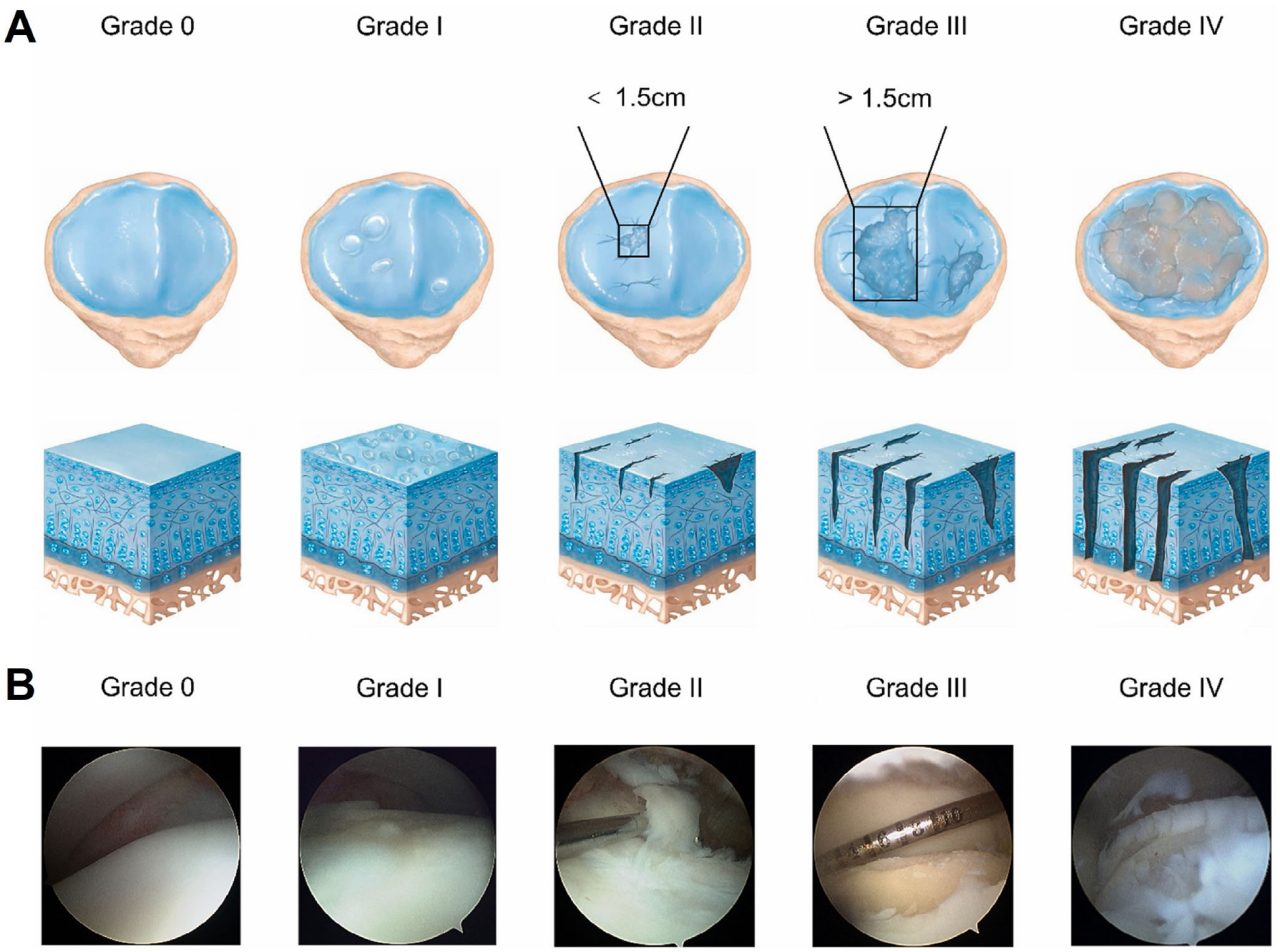
Schematic illustration and arthroscopic images of the Outerbridge classification system. (A) Schematic diagram of the Outerbridge grading system, detailing the following grades: Grade 0 represents normal cartilage; Grade I indicates focal softening and swelling of cartilage; Grade II describes a partial‐thickness cartilage defect with a diameter less than 1.5 cm; Grade III refers to a full‐thickness cartilage defect with a diameter greater than 1.5 cm; and Grade IV denotes an osteochondral defect with full‐thickness cartilage loss and exposed subchondral bone. Reproduced with permission [83]. Copyright 2020, Elsevier. (B) Intraoperative arthroscopic images illustrating the Outerbridge grading system. Reproduced under the terms of the CC BY 4.0 license [22]. Copyright 2025, The Author(s).

The treatment of osteochondral injuries has long represented a major challenge in the fields of orthopedics and sports medicine. Due to the lack of vasculature, nerves, and lymphatic tissue in articular cartilage, its self‐repair capacity is extremely limited; even small‐area injuries are difficult to heal spontaneously and often progressively develop into osteoarthritis [57]. Clinically, osteochondral injuries are classified into five grades (Grade 0–IV) based on injury severity, ranging from normal cartilage (Grade 0), cartilage softening (Grade I), focal defects smaller than 1.5 cm (Grade II), large‐area defects larger than 1.5 cm (Grade III), to complete cartilage wear exposing subchondral bone (Grade IV) (Figure 3). For different degrees of injury, various treatment strategies have been developed clinically, but these methods all have their respective limitations and are unable to achieve true tissue regeneration and functional recovery [58].

For early‐stage injuries (Grade I‐II), conservative treatments include rest, physical therapy, non‐steroidal anti‐inflammatory drugs (NSAIDs), and intra‐articular injections of hyaluronic acid or platelet‐rich plasma (PRP) [59]. These approaches primarily delay disease progression by reducing inflammation, relieving pain, and improving joint lubrication, but cannot repair damaged cartilage tissue. NSAIDs may produce gastrointestinal and cardiovascular side effects, while the efficacy of hyaluronic acid and PRP injections exhibits individual variability with limited duration of action [59, 60].

When injuries progress to the moderate stage (Grade II‐III), microfracture is the most commonly used treatment method [61]. Its principle involves drilling holes in the subchondral bone to release bone marrow mesenchymal stem cells, growth factors, and blood into the defect area, inducing repair tissue formation [62]. Microfracture is simple to perform, minimally invasive, and low‐cost with favorable short‐term outcomes, but its major limitation lies in the repair tissue predominantly consisting of fibrocartilage rather than hyaline cartilage [63]. Fibrocartilage contains abundant type I collagen while lacking type II collagen, with mechanical properties and wear resistance far inferior to native hyaline cartilage, typically exhibiting degradation and failure within 2–5 years postoperatively [64]. Furthermore, microfracture disrupts the integrity of the subchondral bone plate, potentially leading to subchondral bone sclerosis and cyst formation [65].

For larger‐area injuries (Grade III‐IV), osteochondral autograft transplantation (OAT) involves harvesting cylindrical osteochondral plugs from non‐weight‐bearing regions of the patient and transplanting them to the defect site [66]. This method provides mature hyaline cartilage and subchondral bone, immediately offering mechanical support without immune rejection issues [67]. However, donor tissue is limited, typically only capable of repairing defects smaller than 2–4 cm^2^, and harvesting causes donor site morbidity [68]. Gaps between transplanted plugs are filled with fibrous tissue, unable to form a continuous cartilage surface; incomplete integration between transplanted plugs and surrounding tissue leads to stress concentration and degradation at the interface [69]. Long‐term follow‐up demonstrates that clinical outcomes gradually decline 5–10 years postoperatively [70].

Autologous chondrocyte implantation (ACI) involves transplanting chondrocytes to the defect area after in vitro expansion, comprising three generations of technology [71]. First‐generation ACI uses periosteal flap coverage, second‐generation employs collagen membrane replacement, and third‐generation (MACI) seeds chondrocytes onto scaffolds, simplifying surgical procedures [72]. ACI can repair larger‐area defects (exceeding 10 cm^2^), with repair tissue more closely resembling hyaline cartilage. However, ACI requires two surgeries, chondrocytes are prone to dedifferentiation during in vitro culture, and repair tissue often consists only of the cartilage layer, lacking subchondral bone reconstruction, resulting in poor interfacial integration with failure rates of 20%–30% at 5–10 years postoperatively [73, 74].

For end‐stage injuries (Grade IV), joint replacement surgery represents the ultimate option [75]. Total knee arthroplasty (TKA) or total hip arthroplasty (THA) can effectively relieve pain and restore joint function [76]. However, joint replacement surgery is irreversible, with prosthesis lifespan typically ranging from 15–20 years; young patients may require multiple revision surgeries [77]. Furthermore, complications such as periprosthetic infection, prosthetic loosening, polyethylene wear, and osteolysis remain major challenges, and artificial joints cannot fully replicate the biomechanical performance of native joints [78].

Osteochondral allografts are primarily used for large‐area defects (>4 cm^2^) or cases of autograft failure [79]. Although they can provide complete cartilage and bone structures, they face challenges including limited donor availability, immune rejection risk, disease transmission risk, and low graft survival rates, with approximately 70%–85% survival at 5 years and declining to 60%–75% at 10 years [80].

Overall, the fundamental limitations of current treatment methods lie in: lack of precise reconstruction of the complex stratified structure and interfacial characteristics of osteochondral tissue; difficulty in simultaneously meeting the distinct biological and mechanical requirements of cartilage and bone layers; and absence of effective biological signal regulation to guide tissue regeneration and integration [81]. Therefore, developing novel osteochondral tissue engineering strategies that achieve functional regeneration of osteochondral tissue through optimized combinations of biomimetic scaffolds, seed cells, and bioactive factors represents an urgent need in current research [82].

## 3 Decellularization Technology and Validation

Decellularized ECM (dECM) technology removes cellular components from tissues while preserving the three‐dimensional structure, bioactive molecules, and mechanical properties of native ECM, providing biological scaffolds superior to synthetic materials for tissue engineering [84]. This technology has been widely applied in regenerative repair of parenchymal organs and connective tissues and can be processed into various forms such as scaffolds, patches, powders, or hydrogels according to clinical needs [85, 86].

**FIGURE 4:**
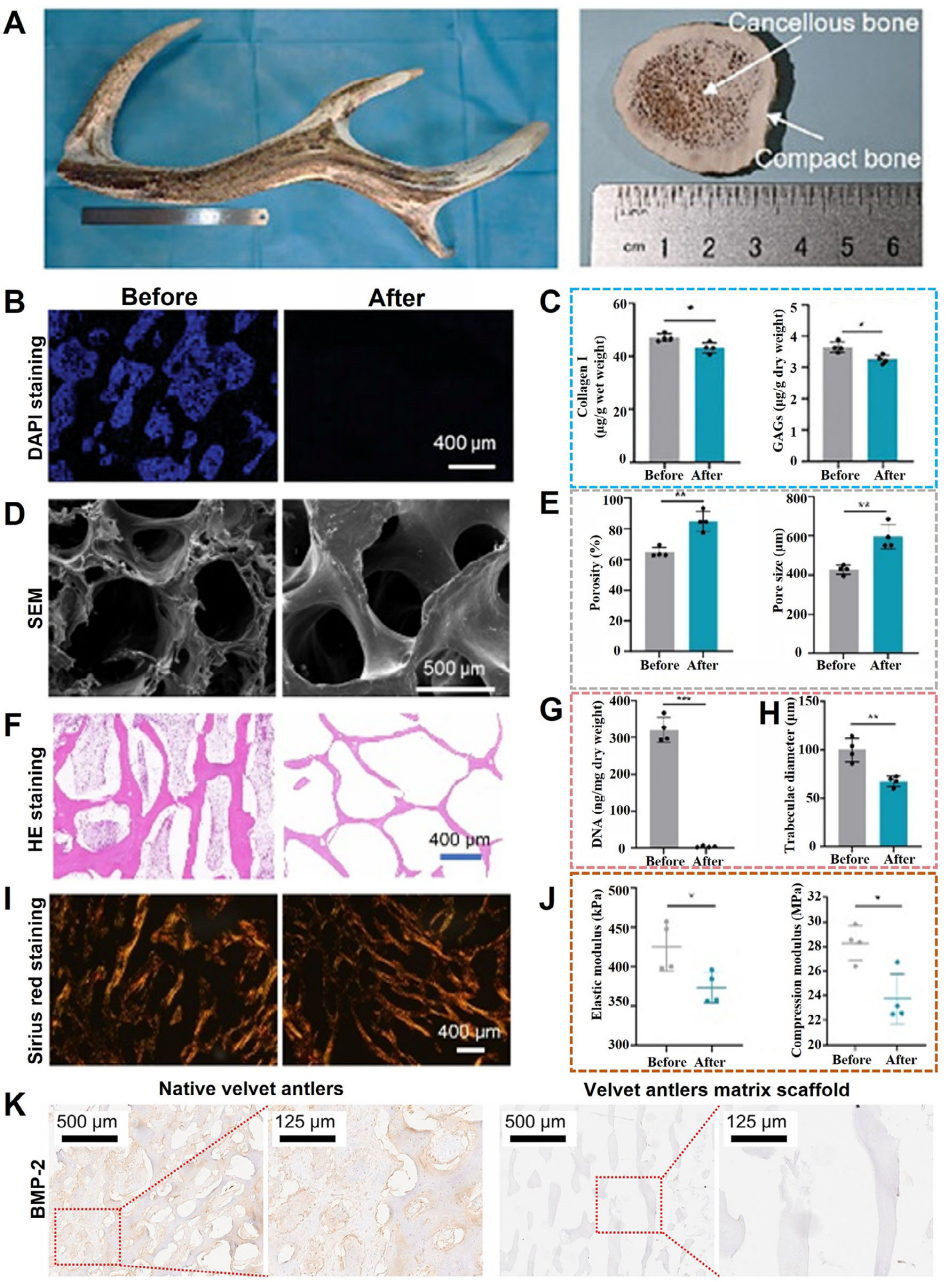
Characterization of native velvet antlers and decellularized antler matrix scaffolds. (A) Naturally shed Sika deer antlers. (B) DAPI staining of antler tissue before and after decellularization. (C) Quantitative analysis of collagen I and GAGs content in antler before and after decellularization (*n* = 4). (D) Scanning electron microscope (SEM) images showing the microstructure of antler before and after decellularization. (E) Porosity and pore size measurements in antler before and after decellularization (*n* = 4). (F) Hematoxylin and eosin (HE) staining of antler before and after decellularization. (G) DNA contents of antler before and after decellularization. (H) Trabecular diameter measurement in antler before and after decellularization (*n* = 4). (I) Sirius red staining of antler before and after decellularization. (J) Elastic modulus and compression modulus measurements in antler before and after decellularization (*n* = 4). (K) Immunohistochemical staining of BMP‐2 in native velvet antlers and decellularized antler matrix scaffolds. Panels A–J reproduced under the terms of the CC BY‐NC‐ND 4.0 license [89]. Copyright 2024, The Author(s); Panel K reproduced under the terms of the CC BY 4.0 license [87]. Copyright 2025, The Author(s).

Taking deer antler as an example (Figure 4A), this rapidly regenerating tissue possesses a natural stratified structure and abundant ECM components, providing an ideal scaffold source for osteochondral repair. Decellularization treatment requires achieving a balance between removing cellular components (reducing immunogenicity) and preserving ECM structure, bioactivity, and mechanical properties. Cell removal was confirmed by DAPI staining (Figure 4B), which demonstrated complete elimination of cell nuclei, with quantitative DNA content analysis (Figure 4G) further confirming effective clearance of nucleic acid components, while biochemical analysis (Figure 4C) demonstrated good preservation of key ECM components such as Collagen I and GAGs, establishing the foundation for scaffold bioactivity. Scanning electron microscopy (Figure 4D) reveals the formation of an open porous network structure after decellularization, with quantitative analysis (Figure 4E,H) confirming significant increases in porosity and pore size along with alterations in trabecular diameter; this three‐dimensional porous structure facilitates cell infiltration, migration, and nutrient exchange. Histological staining (Figure 4F,I) through H&E staining and Sirius Red staining further confirms the thoroughness of decellularization and the integrity of the collagen fiber network. Mechanical testing (Figure 4J) shows that although elastic modulus and compressive modulus decrease after decellularization, the scaffold still maintains sufficient mechanical strength to support tissue regeneration. Notably, immunohistochemistry (Figure 4K) reveals retention of growth factors such as BMP‐2 in decellularized scaffolds, providing an endogenous molecular basis for subsequent osteogenic induction and tissue repair.

The successful practice of deer antler decellularization highlights the importance of method selection. Achieving the ideal “removal‐preservation” balance critically depends on selecting appropriate decellularization methods based on tissue characteristics and precisely optimizing treatment parameters. Current decellularization technologies are primarily categorized into three major classes—physical, chemical, and enzymatic methods—each with unique mechanisms of action, advantages, and limitations [87]. Practical applications typically require combining multiple methods, with personalized protocols designed according to tissue type, density, thickness, and cellular content [88]. For osteochondral tissue, due to its stratified structural characteristics, decellularization strategies require particular optimization to simultaneously meet the distinct requirements of both tissue layers.

### 3.1 Decellularization Technology

#### 3.1.1 Physical Decellularization

Physical decellularization methods disrupt cell membrane integrity through mechanical forces or physical energy, causing intracellular contents to be released and removed from tissues, primarily including freeze‐thaw cycling, mechanical agitation, ultrasonic treatment, and high hydrostatic pressure (Table 2).

**TABLE 2:** Physical methods for tissue decellularization.

Physical method	Core principle	Advantages	Drawback	Reference
Freeze‐thaw cycles	Disrupt cell membranes by forming intracellular ice crystals	Facilitating infiltration of decellularization agents	Disruption of basement membranes; Not sufficient when used solely	[90, 91, 92]
Mechanical methods (Mincing/Chopping, Scraping, Sonication, Agitation)	Mechanically disrupt/remove cells by cutting, scraping, acoustic cavitation or shaking/stirring	Aiding the separation of tissue sub‐layers; Assisting cell removal; Facilitating infiltration of agents	Damage ECM architecture; Need force standards; Not sufficient when used solely	[90, 91, 93]
High hydrostatic pressure	Burst cells and force cellular components out	Efficient cell lysis	Difficult ECM penetration; May denature proteins	[94, 95]
Supercritical fluids	Facilitate chemical exposure for cell removal	Effective cell removal	Not yet widely used	[99]
Non‐thermal irreversible electroporation	Micropore formation in cell membranes	Effective cell lysis	Hard for large tissues	[96, 97, 98]
Perfusion	Remove cells from ECM and help the removal of cellular components within the organ's natural vasculature	Efficient for vascularized tissues	Cannot process tissues without innate vasculature; Sophisticated to operate	[100]

Freeze‐thaw cycling is one of the most commonly used physical decellularization methods. Its principle involves utilizing ice crystals formed during repeated freezing and thawing processes to puncture cell membranes, leading to cell lysis [90]. The typical procedure involves freezing tissues at −80°C or in liquid nitrogen (−196°C), then thawing in a 37°C water bath or at room temperature, repeating for 3–5 cycles. The advantages of freeze‐thaw cycling lie in its simple operation, low cost, and absence of chemical reagent introduction, thus minimally affecting the chemical composition of ECM [91]. However, the decellularization efficiency of freeze‐thaw cycling is relatively low, typically requiring combination with chemical or enzymatic methods to achieve adequate decellularization. Furthermore, ice crystal formation may damage the ultrastructure of ECM; particularly for dense tissues such as cartilage, freeze‐thaw may cause mechanical damage to the collagen fiber network. Studies demonstrate that small ice crystals formed by rapid freezing cause less structural damage than large ice crystals formed by slow freezing; therefore, liquid nitrogen flash‐freezing is superior to slow freezing at −80°C [92].

Mechanical agitation and oscillation promote cell detachment from tissues through sustained mechanical forces. Placing tissues in decellularization solution and continuously agitating at 50–200 rpm on an orbital shaker or magnetic stirrer can enhance reagent penetration, accelerate diffusion of cellular debris, and promote cell detachment from ECM through shear forces. Mechanical agitation is typically not used alone but serves as an adjunct to chemical or enzymatic decellularization, significantly shortening decellularization time and improving decellularization efficiency. For thin sheet‐like tissues (such as pericardium and dermis) or small tissue pieces, mechanical agitation is highly effective; however, for large or dense tissues (such as osteochondral tissue), excessive mechanical force may lead to tissue fragmentation or ECM damage [91, 93].

Ultrasonic treatment utilizes the cavitation effect generated by high‐frequency sound waves (20‐40 kHz) to disrupt cell membranes. Microbubbles produced during ultrasound propagation in liquid rapidly expand and collapse, creating localized high pressure and shear forces that lead to cell lysis [90, 93]. The advantages of ultrasonic decellularization include short processing time (typically minutes to hours) and the ability to precisely control the degree of decellularization by adjusting ultrasonic power, frequency, and treatment duration. However, ultrasonic treatment may also cause significant ECM damage; particularly, high‐power ultrasound can lead to collagen fiber breakage, GAG loss, and decreased mechanical properties. Therefore, ultrasonic decellularization requires careful parameter optimization, typically employing low‐power, intermittent ultrasound (such as 5 seconds on, 10 seconds off) to minimize ECM damage [90].

High hydrostatic pressure (HHP) is an emerging physical decellularization method that disrupts cell membranes and organelles by applying high pressure (typically 200–980 MPa) while maintaining relatively intact ECM structure [94]. The advantages of HHP include uniform treatment, no requirement for chemical reagents, minimal impact on the biochemical composition of the ECM, and certain sterilization effects. Studies demonstrate that HHP can effectively remove cells while preserving collagen, GAGs, and growth factors. However, HHP equipment is expensive, and operation is complex; moreover, for highly mineralized bone tissue, high pressure may affect mineral structure [95].

For osteochondral tissue, physical methods typically serve as the first step or adjunct in decellularization protocols [96]. Freeze‐thaw cycling can pretreat tissues, disrupting cell membranes and creating conditions for subsequent chemical or enzymatic treatment. Mechanical agitation can enhance reagent penetration, particularly for the dense cartilage layer [97, 98]. However, physical methods alone are insufficient to achieve adequate decellularization and must be combined with chemical and enzymatic methods.

#### 3.1.2 Chemical Decellularization

Chemical decellularization methods utilize chemical reagents such as detergents, acidic and alkaline solutions, alcohols, and chelating agents to dissolve cell membranes, disrupt cell‐ECM connections, and remove nucleic acids and lipids, representing the most effective and commonly used decellularization approach [96, 101, 102, 103, 104, 105, 106, 107, 108, 109]. According to their chemical properties, detergents can be classified into three categories: ionic, non‐ionic, and zwitterionic, with each class exhibiting distinct mechanisms of action and effects on ECM (Table 3).

**TABLE 3:** Chemical Method for Tissue Decellularization.

Chemical method	Core principle	Advantages	Drawback	Reference
Alkaline/Acidic solutions (NaOH, Peracetic acid)	Hydrolysis of cellular debris	High removal efficiency	ECM protein damage, GAG loss	[101, 102]
Alcoholic solutions (Ethanol, Glycerol)	Dehydration, protein denaturation	Simple, lipid removal	Incomplete cell removal	[96, 103]
Non‐ionic detergents (Triton X‐100)	Disrupt lipid‐protein bonds	Mild, preserves ECM	Lower efficiency	[104, 105, 106]
Ionic detergents (SDS, Sodium deoxycholate)	Solubilize cell membranes	Strong cell lysis	Severe ECM damage	[107, 108, 109]
Zwitterionic detergents (CHAPS)	Gentle membrane disruption	Less ECM damage	Lower efficiency	[101, 106]
Osmotic solutions (Tris‐HCl, NaCl, KCl)	Osmotic cell lysis	Simple, low cost	Incomplete removal	[110]
Chelating agents (EDTA, EGTA)	Chelate Ca^2^^+^/Mg^2^^+^	Preserves ECM	Needs combination	[111, 112]

Ionic detergents disrupt cell membranes and nuclear membranes through charged groups, dissolving cellular components, and represent the most efficient chemical reagents for decellularization [101, 102]. Sodium dodecyl sulfate (SDS) is the most commonly used anionic detergent, typically at concentrations of 0.1%–1.0%, with treatment durations ranging from hours to days. SDS inserts its hydrophobic alkyl chain and negatively charged sulfate group into the lipid bilayer, disrupting cell and nuclear membranes to release intracellular contents while simultaneously dissolving nucleic acids and proteins [103]. The decellularization efficiency of SDS is extremely high, capable of thoroughly removing cells and DNA residues; however, its potent detergent action also significantly disrupts ECM structure. Studies demonstrate that SDS treatment leads to substantial GAG loss (up to 50%–90%), growth factor inactivation, collagen fiber network disruption, and decreased mechanical properties [107]. Furthermore, SDS residues are cytotoxic and require extensive washing (typically days to weeks) for removal. Sodium deoxycholate (SDC) is another commonly used anionic detergent at concentrations of 1%–4%; its decellularization efficiency is slightly lower than SDS, but it causes relatively less ECM disruption. SDC is more easily washed out with lower residual toxicity; therefore, it is used in some protocols as an alternative to SDS or in combination with SDS [108, 109].

Non‐ionic detergents disrupt lipid‐lipid and lipid‐protein interactions through hydrophobic effects but do not disrupt protein‐protein interactions, thus providing better preservation of ECM structure. Triton X‐100 is the most commonly used non‐ionic detergent, typically at concentrations of 0.1%–1.0%. Triton X‐100 can effectively remove cell membranes and cytoplasmic components but has a weaker capacity to disrupt nuclear membranes; therefore, its DNA removal efficiency is lower than SDS [104]. Its advantages lie in minimal impact on the biochemical composition and mechanical properties of ECM, enabling better preservation of collagen, GAGs, laminin, and growth factors. However, Triton X‐100 also presents residual toxicity issues and is difficult to completely wash out. Studies demonstrate that scaffolds treated with Triton X‐100 require at least 7 days of repeated washing to reach safe levels [105, 106].

Zwitterionic detergents contain both positively and negatively charged groups, exhibiting different ionic properties under varying pH conditions [101]. CHAPS (3‐[(3‐cholamidopropyl)dimethylammonio]‐1‐propanesulfonate) is the most commonly used zwitterionic detergent at concentrations of 8–10 mm. The decellularization efficiency of CHAPS falls between ionic and non‐ionic detergents, with relatively less ECM disruption and easier washout [106]. CHAPS is particularly suitable for processing tissues with high lipid content (such as neural tissue and adipose tissue).

Acidic and alkaline treatments disrupt cell‐ECM connections and hydrolyze nucleic acids and proteins by altering pH values. Acetic acid or hydrochloric acid (pH 2‐3) can dissolve cellular components and remove GAGs, commonly used for decellularization and demineralization of bone tissue. Sodium hydroxide or ammonium hydroxide (pH 11‐12) can hydrolyze nucleic acids and proteins, but also cause severe ECM disruption [110]. Acidic and alkaline treatments are typically used as adjuncts in combination with detergents. Alcohols and organic solvents such as ethanol, isopropanol, and acetone can fix tissues, precipitate proteins, remove lipids, and provide certain sterilization effects. However, alcohols cause protein denaturation, tissue shrinkage, and embrittlement; therefore, they are primarily used in decellularization for defatting or as a final sterilization step [111]. Chelating agents such as EDTA and EGTA disrupt calcium‐dependent connections between cell‐cell and cell‐ECM by chelating Ca^2^^+^ and Mg^2^^+^, promoting cell detachment from tissues. EDTA (typically 0.5–10 mm) is often used in combination with trypsin to enhance enzymatic decellularization efficacy [112].

For osteochondral tissue, the selection of chemical decellularization methods must consider the distinct characteristics of the cartilage and bone layers. The cartilage layer has dense ECM and low cell density, requiring stronger chemical reagents and longer treatment durations to ensure adequate reagent penetration; the bone layer has high mineralization and high cell density, making chemical reagent penetration more difficult. Common strategies include: (1) initial aggressive decellularization with SDS or SDC, followed by gentle treatment with Triton X‐100 to protect ECM; (2) separate treatment of cartilage and bone layers using different concentrations and durations of chemical reagents; (3) freeze‐thaw or mechanical pretreatment before chemical processing to open tissue structure and enhance reagent penetration.

#### 3.1.3 Enzymatic Decellularization

Enzymatic decellularization methods utilize specific enzymes to degrade cellular components or cell‐ECM connections, exhibiting high selectivity and controllability. Compared to chemical methods, enzymatic approaches cause less non‐specific damage to ECM structure; however, decellularization efficiency is typically lower, and enzyme activity is influenced by conditions such as temperature, pH, and ionic strength (Table 4).

**TABLE 4:** Enzymatic method for tissue decellularization.

Enzymatic method	Core principle	Advantages	Drawback	Reference
Nucleases (DNase, RNase)	Degrade nucleic acids (DNA/RNA)	Efficient separation of cellular components	Decreasing bioactive components	[104, 113]
cDispase, Collagenase)	Lysis of cytoplasmic and nuclear constituents	Efficient cell removal	Disruption of ECM structure; Decreasing mechanical properties	[114, 115]
Lipase	Hydrolyze lipid ester bonds	Removal of lipid residues	Decreasing bioactive components	[116]

Trypsin is the most commonly used proteolytic enzyme, cleaving peptide bonds at the carboxyl terminus of lysine or arginine residues to degrade cell membrane proteins and cell‐ECM adhesion proteins, thereby promoting cell detachment from tissues. Trypsin concentrations typically range from 0.05%–0.25%, with treatment at 37°C for hours to days. The advantage of trypsin lies in its ability to effectively remove cells while relatively preserving major ECM structural proteins (such as collagen). However, prolonged or high‐concentration trypsin treatment degrades ECM components, including collagen, laminin, and fibronectin, leading to decreased mechanical properties and loss of biological activity. Furthermore, trypsin has limited capacity for nucleic acid removal and typically requires a combination with nucleases. To enhance trypsin efficiency while reducing ECM damage, it is often used in combination with EDTA (such as 0.05% Trypsin + 0.5 mm EDTA).

Nucleases include deoxyribonuclease (DNase) and ribonuclease (RNase), which degrade DNA and RNA, respectively [104]. DNase I is the most commonly used nuclease at concentrations of 10–100 U/mL, with treatment at 37°C for several hours in Mg^2^^+^‐containing buffer. DNase degrades high‐molecular‐weight DNA into small fragments by cleaving DNA phosphodiester bonds, facilitating removal from tissues by washing. Nuclease treatment is a critical step in decellularization protocols, as DNA residues are the primary cause of immune responses [113]. However, nucleases alone cannot remove intact cells and can only function after cell membranes have been disrupted; therefore, they are typically used following physical or chemical decellularization.

Dispase is a neutral protease that specifically degrades type IV collagen and fibronectin, disrupting basement membranes and cell‐ECM connections [114]. Dispase concentrations range from 1–5 U/mL, with treatment at 37°C for several hours. Dispase has minimal impact on major ECM structural proteins (such as type I and type II collagen); therefore, it is considered a gentler decellularization enzyme [115]. However, the decellularization efficiency of dispase is relatively low, and it is primarily used for thin tissue layers (such as epithelial tissue) or as part of combination protocols.

Lipase degrades lipids and is used to remove cell membranes and lipid‐rich tissues (such as adipose tissue and neural tissue). Exonucleases and endonucleases can further degrade nucleic acid fragments, improving DNA removal efficiency [116].

For osteochondral tissue, enzymatic decellularization methods face special challenges. The dense cartilage ECM impedes enzyme penetration, requiring longer treatment durations or higher enzyme concentrations, which may lead to ECM degradation. The mineralized matrix of bone tissue inhibits enzyme activity, reducing decellularization efficiency. Therefore, enzymatic methods are typically not used alone but rather as part of a combination decellularization protocols. A typical strategy involves: initial disruption of cell membranes and opening of tissue structure using physical methods (freeze‐thaw) or chemical methods (detergents), followed by trypsin treatment to remove cells, and finally DNase treatment to degrade residual DNA.

### 3.2 Decellularization Validation

**FIGURE 5:**
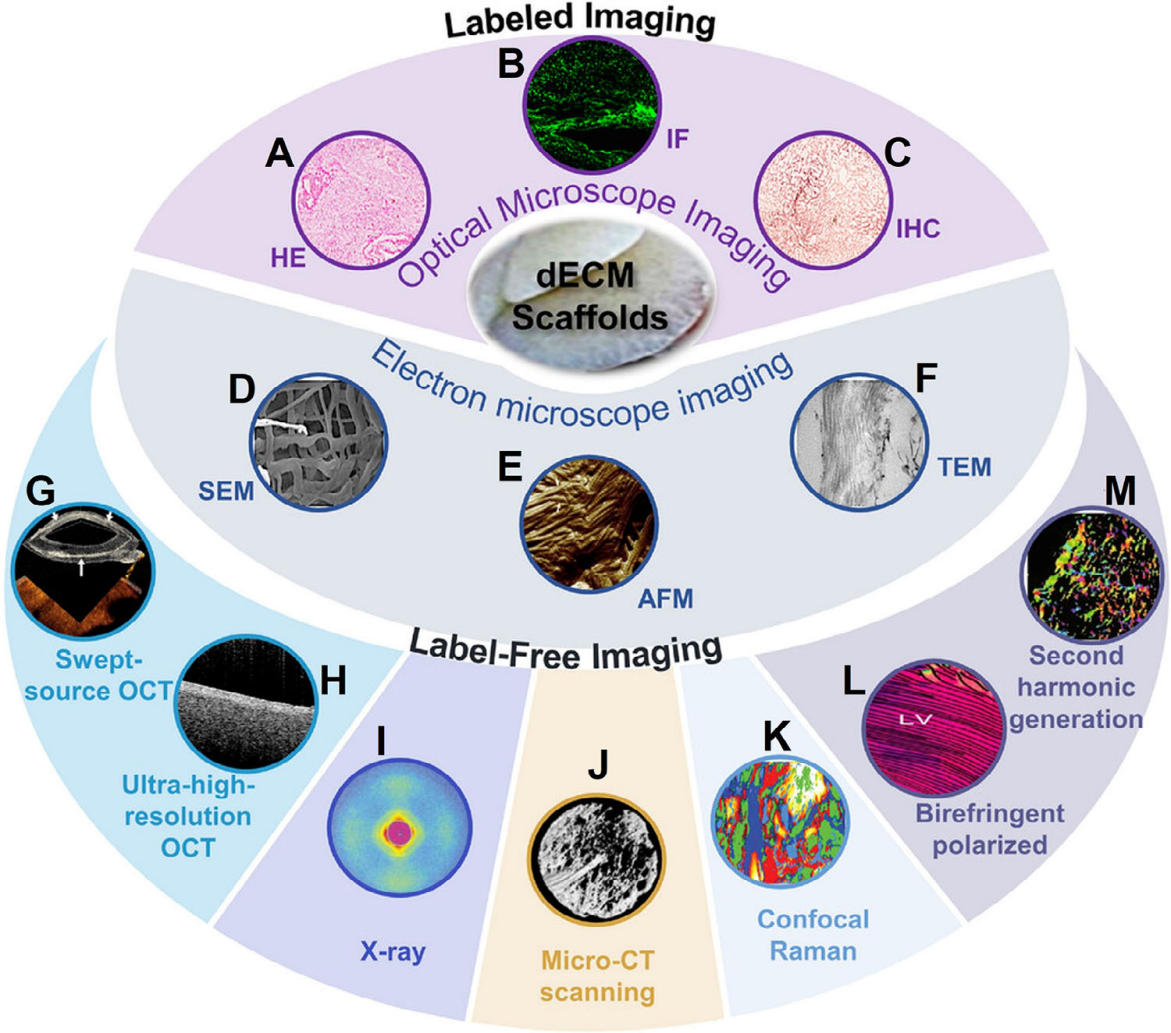
Structural and morphological analysis of dECM scaffolds using various imaging techniques: (A) H&E staining, (B) immunofluorescent staining (collagen IV), (C) immunohistochemical staining, (D) SEM, (E) AFM, (F) TEM, (G) swept‐source OCT, (H) UHR‐SD‐OCT, (I) synchrotron X‐ray scattering, (J) micro‐CT, (K) confocal Raman microscopy, (L) birefringence polarization imaging, and (M) second harmonic generation imaging. Reproduced under the terms of the CC BY 4.0 license [84]. Copyright 2024, The Author(s).

The success of the decellularization process must be comprehensively evaluated through standardized detection methods (Figure 5). Ideal dECM scaffolds should meet the following core criteria: cellular components and DNA residues reduced below safe thresholds, major structural components and bioactive molecules of ECM preserved, mechanical properties approaching those of native tissue, and absence of cytotoxicity and immunogenicity [86]. These validation standards serve not only as the basis for evaluating decellularization efficacy but also as regulatory requirements for clinical translation of dECM products [84].

Histological and immunological validation represents the primary approach for assessing decellularization efficacy. H&E staining (Figure 5A) directly visualizes cell nuclei removal and ECM structural integrity, while immunofluorescence staining (Figure 5B) and immunohistochemistry (Figure 5C) specifically detect the retention of key ECM components (such as type IV collagen) and bioactive molecules [117]. Microstructural characterization reveals the multiscale architecture of dECM scaffolds. Scanning electron microscopy (Figure 5D) displays surface morphology and porous structure, atomic force microscopy (Figure 5E) evaluates nanoscale surface properties and local mechanical performance, and transmission electron microscopy (Figure 5F) observes the ultrastructure of collagen fibers. Advanced imaging techniques enable non‐destructive three‐dimensional assessment. Swept‐source OCT (Figure 5G) and ultra‐high‐resolution OCT (Figure 5H) provide three‐dimensional reconstruction of internal scaffold structure, synchrotron X‐ray scattering (Figure 5I) analyzes collagen fiber orientation, and micro‐CT (Figure 5J) quantitatively evaluates porosity and interconnectivity. Functional imaging techniques provide unique information on ECM composition and organization. Confocal Raman microscopy (Figure 5K) enables label‐free identification of ECM chemical composition, birefringence polarization imaging (Figure 5L) assesses collagen alignment order, and second harmonic generation imaging (Figure 5M) specifically visualizes the three‐dimensional collagen fiber network [118].

For osteochondral tissue, validation standards must also consider its stratified structural characteristics: the cartilage layer requires preservation of type II collagen and GAGs to maintain compressive resistance, the subchondral bone layer requires preservation of mineralized matrix and type I collagen to provide mechanical support, and the interface region requires preservation of gradient structure to enable stress transfer [86]. Therefore, validation of osteochondral dECM necessitates separate assessment of different layers, comprehensively employing multiple technical approaches to fully characterize scaffold properties [119].

#### 3.2.1 DNA Residue Detection

The evaluation of decellularization efficacy primarily relies on assessing DNA residues, which serve as a critical indicator. Residual DNA fragments can be recognized by the host immune system, triggering the Toll‐like receptor 9 (TLR9) signaling pathway. This activation leads to the release of proinflammatory cytokines, potentially resulting in acute or chronic inflammatory responses. Furthermore, residual DNA may interfere with seed cell adhesion, proliferation, and differentiation, affecting tissue regeneration outcomes.

The internationally recognized decellularization criteria were proposed by Crapo et al. [120]. in 2011: (1) DNA content <50 ng/mg dry tissue weight; (2) DNA fragment length <200 bp; (3) no visible nuclear remnants by H&E staining or DAPI staining. These criteria are based on substantial research evidence demonstrating that when DNA residues fall below these thresholds, the immunogenicity of implants is significantly reduced.

DNA quantification primarily employs the PicoGreen/Quant‐iT dsDNA assay, which is the standard method recommended by the FDA and academia. This method is based on the specific binding of fluorescent dyes to double‐stranded DNA, measuring fluorescence intensity via fluorescence spectrophotometry and calculating DNA concentration according to standard curves. It offers high sensitivity (capable of detecting DNA as low as 25 pg/mL), simple operation, and good reproducibility [121]. Spectrophotometric methods (UV absorption) calculate DNA concentration by measuring absorbance at 260 nm wavelength; this approach is rapid but has lower sensitivity and is typically used for preliminary screening.

DNA fragment length detection is achieved through agarose gel electrophoresis. Extracted DNA samples are electrophoresed on 1%–2% agarose gels and compared with DNA molecular weight standards. Adequately decellularized tissues should display diffuse low‐molecular‐weight DNA smears (<200 bp) rather than high‐molecular‐weight genomic DNA bands [122].

Histological detection provides a direct visual assessment of DNA residues. In H&E staining, hematoxylin stains cell nuclei blue‐purple; adequately decellularized tissues should show no obvious blue‐purple nuclear staining. DAPI staining is more sensitive and can detect trace amounts of residual DNA under fluorescence microscopy [123]. However, histological methods are semi‐quantitative and must be used in combination with quantitative methods.

For osteochondral tissue, DNA detection faces special challenges. The dense and negatively charged cartilage ECM adsorbs DNA fragments; minerals in bone tissue interfere with DNA extraction reagents, reducing extraction efficiency [124]. Therefore, digestion and extraction protocols require optimization, such as initial demineralization with EDTA followed by thorough digestion with proteinase K.

#### 3.2.2 ECM Component Retention Assessment

The decellularization process inevitably causes a certain degree of ECM damage, including degradation of structural proteins, loss of bioactive molecules, and disruption of ultrastructure [125]. The degree of ECM component retention directly determines the biological functions of dECM scaffolds, including cell adhesion, proliferation, differentiation, and tissue regeneration capacity [85].

Collagen assessment represents the core indicator of ECM retention. For osteochondral tissue, the cartilage layer primarily contains type II collagen (Col II), while the bone layer primarily contains type I collagen (Col I) [126]. Histological staining provides the most direct assessment method: Masson's trichrome staining colors collagen fibers blue or green, while Picrosirius red staining under polarized light microscopy can distinguish different collagen types (type I collagen appears yellow‐red, type II collagen appears green) [127]. Immunohistochemical staining (IHC) uses specific antibodies to detect the distribution and expression intensity of different collagen types, enabling semi‐quantitative assessment of collagen retention [128]. Biochemical quantitative analysis evaluates total collagen content through hydroxyproline measurement (approximately 13% of collagen protein is hydroxyproline), while Western blot or ELISA can quantitatively detect specific collagen types [129].

Glycosaminoglycan (GAG) assessment is particularly important for cartilage tissue, as GAGs are key components of cartilage compressive resistance [130]. Alcian Blue staining colors sulfated GAGs blue, while Safranin O staining colors GAGs red; staining intensity reflects GAG content [131]. Biochemical quantitative analysis employs the dimethylmethylene blue (DMMB) method, calculating GAG content based on absorbance changes (525 nm or 656 nm) after DMMB binding to sulfated GAGs [132]. GAG loss during decellularization is a common problem, particularly when using ionic detergents (such as SDS), with GAG losses reaching 50‐90%.

Growth factor and cytokine assessment is crucial for the bioactivity of dECM. Growth factors sequestered within the ECM (such as TGF‐β, BMP‐2, VEGF, FGF, etc.) play key regulatory roles in tissue regeneration [133]. ELISA is the most commonly used quantitative method, capable of specifically detecting the content of target growth factors. Immunohistochemical staining can reveal the spatial distribution of growth factors [134]. However, growth factor retention during decellularization represents a major challenge, as chemical reagents and enzymatic treatments lead to growth factor inactivation or elution.

Ultrastructural assessment employs scanning electron microscopy (SEM) and transmission electron microscopy (TEM) to observe the three‐dimensional network structure [135], fiber arrangement, and porosity of ECM. SEM can display the arrangement of collagen fiber bundles, surface morphology, and porous structure, while TEM can observe the periodic cross‐striations (D‐periodicity, approximately 67 nm) and ultrastructural integrity of collagen fibers [136]. Adequately decellularized tissues with well‐preserved ECM should display ordered fiber networks and open porous structures, whereas excessive processing leads to fiber breakage and structural collapse.

#### 3.2.3 Mechanical Property Assessment

Mechanical properties represent critical indicators of whether dECM scaffolds can withstand physiological loads and maintain tissue structural integrity [137]. Loss of ECM components and structural disruption during decellularization lead to decreased mechanical performance, affecting the clinical application of scaffolds [138]. Osteochondral tissue possesses unique mechanical characteristics: the cartilage layer primarily bears compressive stress, requiring evaluation of compressive modulus and creep behavior; the subchondral bone layer bears tensile and shear stress, requiring evaluation of elastic modulus and tensile strength [139].

Compression testing is the standard method for evaluating cartilage mechanical properties [140]. A materials testing machine applies uniaxial compressive loads to cylindrical samples, recording stress–strain curves and calculating compressive modulus (the slope of the linear region of the stress–strain curve). Native articular cartilage has a compressive modulus of approximately 0.5–2 MPa; adequately decellularized cartilage dECM with well‐preserved ECM should approach this range [85]. Creep testing evaluates the time‐dependent deformation of cartilage under constant load, reflecting its viscoelastic properties.

Tensile testing evaluates the tensile properties of bone tissue and cartilage. Samples are fabricated into dumbbell shapes and subjected to uniaxial tensile loads until fracture, recording stress–strain curves and calculating elastic modulus, ultimate tensile strength, and elongation at break [141]. Native bone tissue has an elastic modulus of approximately 10–20 GPa and ultimate tensile strength of approximately 50–150 MPa; native cartilage has a tensile modulus of approximately 5–25 MPa [100]. Mechanical properties after decellularization typically decline to some extent but should remain above 50%–80% of native tissue.

Nanoindentation testing can evaluate local mechanical properties at the micron scale, making it particularly suitable for assessing the gradient mechanical properties of the osteochondral interface region [142]. Dynamic mechanical analysis (DMA) evaluates the viscoelastic response of materials under cyclic loading, including storage modulus, loss modulus, and damping factor, more closely approximating mechanical behavior under physiological conditions [143].

#### 3.2.4 Immunogenicity and Biocompatibility Testing

Even when DNA residues meet standards, dECM scaffolds may still contain other immunogenic components, such as α‐Gal antigen (the major antigen in xenotransplantation), residual detergents, and cellular debris [144]. Therefore, immunogenicity and biocompatibility testing are necessary validations prior to clinical application of dECM [92].d

In vitro cytotoxicity testing represents the most fundamental biocompatibility assessment. dECM extracts are co‐cultured with cells (such as fibroblasts or mesenchymal stem cells), and cell viability is evaluated through MTT, CCK‐8, or Live/Dead staining. Cell survival rates should exceed 80% to be considered free of significant cytotoxicity [145]. Cell adhesion and proliferation testing evaluates the adhesion, spreading, and proliferation capacity of cells on dECM scaffolds, reflecting the bioactivity of scaffolds.

In vivo immune response assessment is the ultimate validation standard. dECM is implanted into animal models (such as mouse subcutaneous, rat intramuscular, or rabbit osteochondral defects), and samples are collected at different time points for histological assessment of the type and degree of inflammatory response. H&E staining observes inflammatory cell infiltration (neutrophils, macrophages, lymphocytes) and fibrous capsule formation [146]. Immunohistochemical staining detects inflammatory markers (CD68 macrophage marker, CD3 T cell marker) and polarization states (M1 proinflammatory vs. M2 reparative macrophages). Ideal dECM should induce constructive remodeling responses dominated by M2‐type macrophages rather than chronic inflammation. Detection of α‐Gal antigen is particularly important for xenogeneic dECM (such as porcine or bovine sources). This galactose‐α‐1,3‐galactose (α‐Gal) epitope is a glycosylated antigen on the cell surface of non‐primate mammals; the human body contains natural anti‐α‐Gal antibodies that trigger hyperacute rejection reactions [147]. ELISA or immunohistochemistry is used to detect α‐Gal residues, ensuring their content is reduced to safe levels.

Validation of decellularization efficacy is a multidimensional, multilevel systematic endeavor requiring comprehensive integration of multiple indicators, including DNA residues, ECM retention, mechanical properties, and immunogenicity [148]. For osteochondral tissue, particular attention must be paid to differential assessment of stratified structures [149]. Only through rigorous validation standards can the safety and efficacy of dECM scaffolds be ensured, establishing a foundation for clinical application.

## 4 Human‐Derived Materials

Human‐derived materials refer to tissues, cells, or ECM sourced from the patient's own body or other human bodies, used as biomaterials for tissue engineering and regenerative medicine. Compared to xenogeneic materials (such as porcine or bovine sources), human‐derived materials possess superior biocompatibility, lower immunogenicity, and biological properties more closely resembling native tissue, thus offering unique advantages in osteochondral repair [150]. Human‐derived materials are primarily classified into two major categories: autologous and allogeneic sources, each with distinct characteristics regarding immunogenicity, availability, and clinical application.

Autologous materials are harvested from the patient's own body, completely avoiding immune rejection reactions and representing the “gold standard”; allogeneic materials are obtained from other donors, enabling batch preparation and storage, thereby addressing the limited availability of autologous materials [151]. However, both face their respective challenges: autologous materials present donor site morbidity and limited quantity issues; allogeneic materials require rigorous donor screening, viral inactivation, and immunogenicity control [152]. This chapter will separately discuss the current application status, advantages, limitations, and future development directions of these two categories of human‐derived materials in osteochondral repair.

It should be noted that, unlike the non‐human‐derived materials discussed in subsequent sections—which are primarily evaluated based on preclinical evidence—the human‐derived approaches presented in this section largely reflect strategies that have already been translated into clinical practice. Furthermore, some of the approaches discussed herein, including autologous chondrocyte implantation (ACI), mesenchymal stem cells (MSCs), and platelet‐rich plasma (PRP), are not strictly classified as biomaterials in the conventional sense. Rather, they represent established clinical repair techniques or biological therapies, and are included here to provide relevant clinical context for osteochondral repair. Each of these approaches has been extensively reviewed elsewhere, and a comprehensive discussion falls beyond the scope of the present review.

### 4.1 Autologous Materials

Autologous materials refer to tissues, cells, or ECM harvested from the patient's own body, which are processed in vitro and then used to repair the patient's own osteochondral defects [100]. Since the materials are derived from the patient themselves, they possess complete histocompatibility, present no immune rejection reactions, and carry no risk of disease transmission, thus being considered the safest and most ideal repair materials [153]. Autologous materials have diverse application forms in osteochondral repair, including autologous osteochondral transplantation, autologous chondrocytes, autologous mesenchymal stem cells, and autologous blood‐derived products.

#### 4.1.1 Autologous Osteochondral Transplantation

Autologous osteochondral transplantation (AOT), also known as mosaicplasty, is the most classic autologous material application technique [154]. This technique harvests cylindrical osteochondral plugs with diameters of 4–10 mm from non‐weight‐bearing areas of the patient's knee joint (such as the intercondylar notch or patellofemoral joint margins) and transplants them to the defect site, where they are fixed by press‐fit [155]. The greatest advantage of autologous osteochondral transplantation is that the graft contains an intact cartilage layer, calcified cartilage layer, and subchondral bone layer, immediately providing a native articular cartilage surface and mechanical support.

Clinical studies have demonstrated that AOT achieves favorable short‐term and mid‐term outcomes for small to moderate cartilage defects (<2–4 cm^2^) [156]. Long‐term follow‐up studies by Gruson et al. [157] showed that patients with femoral condyle defects achieved 92% excellent‐to‐good results at 10 years postoperatively, while those with patellar defects achieved 79%. The transplanted cartilage maintains the histological characteristics of hyaline cartilage and integrates well with surrounding cartilage. However, AOT also has significant limitations: (1) Donor site morbidity: the harvest site may experience pain, cartilage degeneration, joint stiffness, and other problems, with an incidence of approximately 3%–10%; (2) Limited availability: the quantity of osteochondral tissue in non‐weight‐bearing areas is limited, typically only sufficient to repair defects <4 cm^2^, rendering it inadequate for large defects; (3) Surface irregularity: gaps exist between multiple cylindrical grafts, preventing complete reconstruction of a smooth articular surface and potentially affecting long‐term outcomes; (4) Requirement for secondary surgery: harvesting and transplantation typically must be completed in the same surgical procedure, increasing operative time and trauma.

To overcome these limitations, researchers have developed composite techniques combining microfracture with autologous osteochondral transplantation, as well as modified methods such as autologous osteochondral particulate transplantation [158]. The latter involves fragmenting autologous osteochondral tissue into small particles (0.5–1 mm), which are mixed with fibrin glue or other carriers and then used to fill defects, allowing more flexible adaptation to irregular defect shapes while reducing donor site injury.

#### 4.1.2 Autologous Chondrocyte Transplantation

Autologous chondrocyte implantation (ACI) is the first cell therapy technique approved by the FDA, ushering in a new era of cartilage regeneration [72]. The basic procedure of ACI includes: (1) arthroscopic harvesting of a small amount of cartilage tissue (approximately 200–300 mg) from non‐weight‐bearing areas; (2) in vitro isolation and expansion of chondrocytes to 12–48 million cells (typically requiring 3–5 weeks); (3) a second surgery to inject the cell suspension into the defect site, which is then covered and sealed with a periosteal flap or collagen membrane.

The main advantage of autologous chondrocyte implantation (ACI) is that only a small donor tissue sample is needed to generate large numbers of cells via in vitro expansion, theoretically enabling repair of defects of any size. Long‐term clinical studies indicate that ACI yields favorable outcomes for large cartilage defects (>2 cm^2^), with 60%–80% of patients achieving excellent or good results at 10–20 years postoperatively. For example, Peterson et al. [159] reported that 92% of femoral condyle and 67% of patellar defect cases showed good or excellent outcomes after 20 years.

However, first‐generation ACI has notable limitations: it requires two surgeries, increasing patient burden and cost; periosteal flap complications such as hypertrophy, calcification, or detachment occur in 10%–25% of cases; chondrocyte dedifferentiation during monolayer culture leads to fibrocartilage formation; and the procedure demands advanced surgical skills [160].

To address these issues, ACI has evolved through three generations. Second‐generation ACI (ACI‐C) replaces the periosteal flap with a collagen membrane, reducing complications and enabling minimally invasive arthroscopic fixation. Clinical results are comparable to the first generation, but with fewer adverse events. Third‐generation ACI (MACI) uses three‐dimensional scaffolds for cell delivery, offering more uniform cell distribution, shorter operative times, minimally invasive implantation, and better maintenance of the chondrocyte phenotype [161]. MACI is approved in Europe and the US, with randomized trials showing efficacy equal to or better than microfracture.

Newer approaches include Spherox, which delivers chondrocyte spheroids directly into defects without scaffolds, and AMIC, which combines microfracture with collagen membranes to stimulate endogenous cartilage regeneration without cell culture [162].

#### 4.1.3 Autologous Mesenchymal Stem Cells

Mesenchymal stem cells (MSCs) possess multipotent differentiation potential, self‐renewal capacity, and immunomodulatory functions, making them ideal seed cells for osteochondral tissue engineering [163]. Autologous MSCs can be harvested from various tissues, including bone marrow, adipose tissue, synovium, and umbilical cord, among which bone marrow‐derived mesenchymal stem cells (BMSCs) and adipose‐derived mesenchymal stem cells (ADSCs) are the two most commonly used types [164].

BMSCs are the earliest discovered and studied MSCs, possessing robust osteogenic and chondrogenic differentiation capabilities. BMSCs are typically obtained through iliac crest aspiration, followed by density gradient centrifugation to isolate mononuclear cells, which are then cultured under adherent conditions to obtain MSCs. BMSCs can differentiate into chondrocytes in culture medium containing growth factors such as TGF‐β and BMP‐2, expressing cartilage markers including type II collagen, aggrecan, and Sox9. Clinical studies have shown that autologous BMSCs combined with scaffold materials (such as collagen membranes or hyaluronic acid hydrogels) for cartilage defect repair can promote hyaline cartilage‐like tissue formation and improve clinical symptoms [165]. However, BMSCs also have limitations: (1) Significant harvest‐related trauma: bone marrow aspiration causes pain and bleeding; (2) Low cell numbers: the MSC content in bone marrow is extremely low (approximately 0.001%–0.01%), requiring prolonged in vitro expansion; (3) Age‐related decline: with advancing age, the quantity and differentiation capacity of BMSCs decrease significantly.

Adipose‐derived mesenchymal stem cells (ADSCs) have emerged as an alternative cell source receiving considerable attention in recent years. ADSCs are obtained through liposuction, offering minimal trauma, abundant availability (MSC content in adipose tissue is 500 times that of bone marrow), and rapid expansion [166]. ADSCs possess multipotent differentiation potential and immunomodulatory functions similar to BMSCs, demonstrating promising application prospects in cartilage tissue engineering [167]. Animal experiments have shown that ADSCs combined with hydrogels or nanofiber scaffolds can effectively repair osteochondral defects and form hyaline cartilage‐like tissue. However, some studies have indicated that the chondrogenic capacity of ADSCs is slightly weaker than that of BMSCs, with a greater tendency toward osteogenic differentiation [168].

Synovium‐derived mesenchymal stem cells (SMSCs) are MSCs from articular synovial tissue, possessing the strongest chondrogenic differentiation capacity and being considered the “optimal” stem cells for cartilage repair. SMSCs can be obtained through arthroscopic synovial biopsy with minimal trauma, and being homologous to articular cartilage, are more suitable for cartilage regeneration [169]. However, the obtainable quantity of SMSCs is limited, and their clinical application remains in the exploratory stage.

Clinical application forms of autologous MSCs include: (1) Direct injection: intra‐articular injection of expanded MSC suspension, which is simple but has low cell retention rates; (2) Scaffold loading: seeding MSCs onto three‐dimensional scaffolds and implanting them into defect sites, providing structural support and cellular microenvironment; (3) Microfracture combined with MSCs: injecting MSCs or covering with MSC‐containing membranes after microfracture surgery to enhance endogenous repair.

The biological origin of autologous MSCs fundamentally shapes their chondrogenic potential, osteogenic tendency, and clinical applicability, with SMSCs offering the greatest chondrogenic capacity, BMSCs providing balanced osteo‐chondrogenic potential, and ADSCs excelling in availability but with relatively weaker chondrogenic differentiation. Accordingly, the choice of MSC source should be tailored to the specific repair target and individual patient factors, as tissue origin remains a critical determinant of osteochondral repair outcomes.

#### 4.1.4 Autologous Blood‐Derived Products

Platelet‐rich plasma (PRP) is a platelet concentrate obtained through centrifugal concentration of autologous whole blood, containing high concentrations of growth factors (such as PDGF, TGF‐β, VEGF, IGF‐1, etc.) and cytokines [170]. Upon activation, PRP releases growth factors that promote cell proliferation, angiogenesis, and tissue repair. Application forms of PRP in osteochondral repair include: (1) Intra‐articular injection: used for conservative treatment of early osteoarthritis to relieve pain and improve joint function; (2) Combined with microfracture or ACI: injecting PRP into defects or mixing with cells during surgery to enhance repair outcomes; (3) As a cell culture supplement: PRP can replace fetal bovine serum to promote proliferation and differentiation of chondrocytes and MSCs.

Clinical studies show controversy regarding the efficacy of PRP. Some studies have demonstrated that PRP can improve clinical scores and imaging outcomes of cartilage defect repair [171], but meta‐analyses have also indicated that the effects of PRP are limited and highly dependent on factors such as preparation methods, platelet concentration, and activation approaches. The advantages of PRP lie in its simple preparation, convenient availability, and high safety profile, but its mechanisms of action and optimal application protocols require further investigation.

Autologous fibrin glue is prepared from the patient's own plasma, containing fibrinogen, thrombin, and fibrin stabilizing factor, and can serve as a natural carrier and adhesive for cells and tissue fragments. Fibrin glue is used in osteochondral repair to fix grafts, seal defects, and load cells and growth factors. Compared to commercial fibrin glue, autologous fibrin glue has no immunogenicity or disease transmission risk, but its preparation process is more complex.

#### 4.1.5 Advantages and Limitations of Autologous Materials

The greatest advantage of autologous materials lies in their complete tissue compatibility and safety. Since the materials are harvested from the patient's own body, there is no immune rejection, eliminating the need for immunosuppressive drugs and avoiding risks associated with long‐term immunosuppression, such as infection and malignancy [172]. Meanwhile, autologous materials completely eliminate the possibility of disease transmission, with no risk of viral (such as HIV, HCV, HBV), prion, or bacterial infections—issues that allogeneic and xenogeneic materials cannot entirely circumvent. Furthermore, clinical application of autologous materials faces fewer ethical and regulatory barriers, as the FDA classifies autologous cell therapies as “minimal manipulation” or “homologous use,” with relatively streamlined approval processes. Patient acceptance of using their own tissues to repair their own defects is also high, with minimal psychological burden and good compliance.

However, autologous materials also have significant limitations. First, the harvesting process causes donor site complications, such as additional surgical trauma, pain, bleeding, infection risk, and functional impairment of donor site tissue, which is particularly prominent in autologous osteochondral transplantation [173]. Second, the obtainable quantity of autologous tissue is limited, making it difficult to meet the repair demands of large defects (>4 cm^2^) or multiple‐site defects, restricting its clinical application scope [155]. Third, the quality of autologous materials is significantly influenced by patient‐specific factors; age, disease status (such as osteoarthritis or diabetes), and lifestyle factors (such as smoking or obesity) all reduce cell viability and tissue regeneration capacity, with autologous material quality often being suboptimal in elderly patients or those with severe conditions. Fourth, autologous cell therapies (such as ACI) require in vitro isolation, culture, and expansion, typically requiring 3‐6 weeks, precluding immediate use, prolonging treatment duration, and increasing patient waiting time and the risk of secondary surgery. Finally, the individualized preparation, quality control, and cell culture facilities (GMP laboratories) for autologous materials all require substantial costs, limiting their dissemination in primary healthcare institutions [174].

### 4.2 Allogeneic Materials

Allogeneic materials refer to tissues, cells, or extracellular matrices obtained from allogeneic donors of the same species, which are processed and used for osteochondral repair [175]. Compared to autologous materials, allogeneic materials can be obtained in bulk, standardized in preparation, and stored long‐term, enabling “off‐the‐shelf” availability, avoiding donor site complications and in vitro culture waiting time. Over one million allogeneic bone grafts are used annually in the United States [176]. However, allogeneic materials face challenges, including immune rejection, disease transmission risk, and limited donor availability. How to maximize preservation of tissue function while ensuring safety is the core issue in allogeneic material development.

#### 4.2.1 Allogeneic Osteochondral Transplantation

Allogeneic osteochondral transplantation (OCA) is an important surgical approach for treating large osteochondral defects, particularly suitable for complex cases such as trauma, osteonecrosis, and post‐tumor resection reconstruction. OCA provides a complete stratified structure (hyaline cartilage, calcified cartilage layer, and subchondral bone), capable of immediately restoring articular surface and weight‐bearing function [177].

Fresh allogeneic grafts (Fresh OCA) are implanted within 14 days after donor death, with chondrocyte viability reaching 70%–90%, maintaining cartilage metabolic activity. Long‐term follow‐up studies have shown that fresh OCA demonstrates favorable survival rates, with 10‐year survival ranging from 71%–87.9% and outcomes varying by anatomical location. A systematic review by Assenmacher et al. [178] of 291 patients with a mean 12.3‐year follow‐up revealed that 67% of lesions were located on femoral condyles, which showed significantly better outcomes compared to patellofemoral lesions. However, fresh OCA has limitations, including donor scarcity, need for rapid matching, higher immunogenicity, disease transmission risk, and short storage time (maximum 28–42 days).

Frozen allogeneic grafts (Frozen OCA) are preserved long‐term through deep cryopreservation (−80°C or liquid nitrogen), allowing establishment of tissue banks for on‐demand supply. However, freezing causes massive chondrocyte death (viability <10%) and ECM structural damage, with clinical efficacy significantly lower than fresh OCA and 5‐year failure rates as high as 30%–50% [179]. Novel preservation technologies such as controlled‐rate freezing, vitrification, hypothermic preservation, and organ culture systems are under development to improve cell viability and extend preservation time.

#### 4.2.2 Allogeneic Decellularized Extracellular Matrix (dECM)

Allogeneic decellularized ECM (dECM) removes cellular components and immunogenic substances while preserving ECM structure and bioactivity, representing an important strategy for addressing immune rejection and disease transmission issues [180]. Human‐derived dECM exhibits better biocompatibility and biochemical composition closer to native tissue compared to xenogeneic dECM.

Human osteochondral dECM typically employs mild decellularization protocols, such as low‐concentration SDS (0.1%–0.5%) combined with nucleases, or Triton X‐100 combined with hypertonic salt solutions, to maximally preserve ECM components. Commercialized products include GraftJacket (human decellularized dermis), AlloPatch (human decellularized fascia), and Cartiform (human decellularized cartilage matrix), which are already in clinical use. Clinical studies have shown that human cartilage dECM combined with microfracture or autologous cells can promote hyaline cartilage‐like tissue regeneration [181].

The advantages of human‐derived dECM include low immunogenicity, capability for batch preparation and storage, retention of bioactivity, and malleability for processing into various forms. Limitations include restricted donor availability, significant quality variation, mechanical property degradation due to the decellularization process, and the need for rigorous disease screening.

#### 4.2.3 Allogeneic Cell Therapy

Allogeneic cell therapy uses cells from healthy donors (typically MSCs or chondrocytes) for osteochondral repair, avoiding donor site complications associated with autologous cells and enabling “off‐the‐shelf” cell products.

Allogeneic MSCs have become an ideal choice due to their low immunogenicity and immunomodulatory functions. MSCs express low levels of MHC‐I, do not express MHC‐II or co‐stimulatory molecules, and can secrete immunosuppressive factors (IDO, PGE2, TGF‐β), creating a local immune tolerance environment. Commercialized products such as Cartistem (allogeneic umbilical cord blood MSCs with hyaluronic acid scaffold) have been approved in South Korea, with clinical studies demonstrating its ability to promote cartilage regeneration without severe adverse reactions [182]. JointStem (allogeneic adipose‐derived MSCs) is administered via intra‐articular injection to treat osteoarthritis, relieving pain and improving joint function [183].

Challenges of allogeneic cell therapy include unclear long‐term immune responses, controversy over cell survival and functional maintenance, and more stringent regulatory requirements.

#### 4.2.4 Tissue Bank Management and Quality Control

The safety and efficacy of allogeneic materials are highly dependent on rigorous tissue bank management and quality control. The American Association of Tissue Banks (AATB) and the FDA have established detailed standards [184].

Donor screening includes medical history review (excluding infectious diseases, malignancies, etc.), serological testing (HIV, HBV, HCV, etc.), and microbiological culture, with donor exclusion rates typically reaching 30%–50%. Tissue processing includes cleaning, decellularization, and sterilization (irradiation, chemical sterilization, supercritical CO_2_ sterilization) [185]. Quality control encompasses sterility testing, viral safety, biological performance, and mechanical property testing. Traceability systems ensure complete documentation of each tissue from donor to recipient.

#### 4.2.5 Advantages and Limitations of Allogeneic Materials

The primary advantages of allogeneic materials lie in their accessibility and ready‐to‐use nature. They can be obtained centrally from organ donors, processed through standardized procedures to establish tissue banks, enabling on‐demand supply without waiting for culture, avoiding donor site trauma, and are particularly suitable for large defects, multi‐site defects, or emergency repair cases [184]. Tissue quality obtained from young healthy donors is not affected by the recipient patient's age or disease status, making them suitable for elderly or severely ill patients. Large‐scale production can reduce unit costs and improve healthcare resource utilization efficiency [186].

However, allogeneic materials also face significant limitations. The risk of immune rejection cannot be completely eliminated, particularly with fresh grafts containing viable cells. Despite rigorous screening and sterilization protocols, the possibility of viral, prion, or bacterial transmission cannot be entirely excluded [187]. Human tissue supply is far from meeting clinical demand, resulting in high costs and a shortage. Factors such as different donors' age, health status, cause of death, and ischemic time affect tissue quality, making complete standardization difficult [188]. Processing and storage procedures (decellularization, freezing, irradiation) damage the ECM structure and bioactivity, reducing mechanical properties and regenerative potential.

## 5 Non‐Human‐Derived Materials

Non‐human‐derived materials occupy an important position in osteochondral tissue engineering, primarily comprising two major categories: xenogeneic animal‐derived materials and synthetic materials [189]. Compared to human‐derived materials, non‐human‐derived materials possess significant advantages, including wide availability, low cost, and scalability for mass production, making them the most widely used biomaterial types in current clinical applications. Xenogeneic animal‐derived materials retain the biological characteristics and ECM structure of native tissues, providing favorable cellular microenvironments and biological signals; synthetic materials possess controllable physicochemical properties and mechanical performance, allowing precise design according to specific requirements [190]. However, non‐human‐derived materials also face challenges, including immunogenicity, biocompatibility, and degradation rate matching, requiring optimization of their performance through strategies such as material modification, surface functionalization, and composite formation [191].

To provide a comprehensive overview of these materials, Table 5 summarizes the major categories of non‐human‐derived materials, their sources, advantages, limitations, key characteristics, representative preclinical findings in osteochondral repair models, and available clinical/translational evidence.

**TABLE 5:** Summary of non‐human‐derived materials for osteochondral tissue engineering.

Origin category	Representative biomaterials	Biological source	Advantages	Limitations	Key characteristics and osteochondral repair evidence	Clinical/translational evidence	Reference
Xenogeneic animal‐derived materials	Decellularized ECM: SIS, DCM, DBM	Porcine small intestine; xenogeneic cartilage; xenogeneic bone	Native bioactivity	Immunogenicity/ batch variability	SIS‐KGN promoted rabbit cartilage repair; DCM enhanced chondrogenesis; DBM supported synchronized cartilage‐bone regeneration	DBM has bone repair applications; osteochondral use remains mostly preclinical	[13, 192]
	Other decellularized matrices	Squid cartilage; fish scales; sea cucumber body wall; eggshell membrane; deer antler	Diverse sources	Poor standardization	Rich in collagen/GAGs; supported cartilage matrix synthesis	Early preclinical stage	[87, 89]
	Collagen‐based proteins: type I/II collagen, gelatin/GelMA, collagen peptides	Bovine/porcine/fish tissues; animal cartilage; collagen hydrolysates	Good cell adhesion	Weak mechanics/rapid degradation	Type I collagen for bone layer; type II collagen maintained cartilage phenotype; bilayer Col II scaffold promoted rabbit OCD repair	Chondro‐Gide, NOVOCART 3D, BioCartilage and related products	[193]
	Other ECM proteins: FN, LN, fibrin	Animal tissue ECM; blood‐derived proteins	Adhesion/ delivery	Weak structural support	Used as surface modifiers or cell carriers to improve cell–material interactions	Tisseel and Evicel are clinically used	[194]
	Animal‐derived polysaccharides: chitosan, CS, animal‐derived HA	Crustacean shells; animal cartilage; rooster comb/bovine vitreous	GAG‐mimetic/anti‐inflammatory	Weak mechanics /source variability	Chitosan promoted M2 polarization; CS‐Zn scaffold promoted porcine OCD repair; HA maintained cartilage homeostasis	BST‐CarGel; HA injection; Hyalograft C and Hyalofast	[132, 195]
	Silk proteins: silk fibroin/sericin	Bombyx mori cocoons; wild silkworm silk	Good mechanics/ tunable degradation	Few cell‐recognition sites	Silk/PCL scaffold promoted rat OCD repair; sericin showed anti‐inflammatory/antioxidant effects	Silk sutures are clinically used; OCD scaffolds remain preclinical	[196]
Plant/algae‐derived materials	Algal polysaccharides: alginate, agarose, carrageenan	Brown algae; red algae	Mild gelation	Weak adhesion/ mechanics	GG/alginate hydrogel promoted rabbit OCD stratified regeneration; agarose maintained chondrocyte phenotype	Mainly preclinical	[197]
	Plant polysaccharides: cellulose derivatives, LBG/LBG‐MA	Plant cell walls; carob tree seeds	Abundant/ easily modified	Weak cell adhesion	CMC/gelatin/nHA hydrogel promoted osteochondral repair; LBG‐MA promoted hyaline cartilage‐like repair in rabbits	Mainly preclinical	[198]
Microbial‐derived materials	Bacterial polysaccharides: BC, microbial HA, GG	Acetic acid bacteria; Streptococcus; Sphingomonas elodea	High purity/ stability	Limited adhesion or mechanics	BC reinforced mechanics; HAMA modulated immunity; GG promoted rabbit OCD stratified regeneration	Clinical‐grade HA production is mature; scaffold use needs further translation	[199, 200]
	PHA/PHB/PHBV	Pseudomonas, Bacillus, and other bacteria	Biodegradable/ printable	Hydrophobic/ weak adhesion	PHBV/HAp supported MSC osteogenic and chondrogenic differentiation	Animal experimental stage	[201, 202]

### 5.1 Xenogeneic Animal‐Derived Materials

Xenogeneic animal‐derived materials refer to tissues, ECM, or biological macromolecules obtained from non‐human animals (such as pigs, cattle, horses, sheep, etc.) that are appropriately processed for human osteochondral repair. Compared to human‐derived materials, xenogeneic materials possess advantages including abundant supply, low cost, controllable quality, and standardized production capability, making them the primary source of current commercialized biomaterials. However, the core challenge of xenogeneic materials lies in immunogenicity, particularly xenoantigens such as α‐Gal (galactose‐α‐1,3‐galactose) that may trigger hyperacute rejection reactions [203]. Therefore, xenogeneic materials typically require methods such as decellularization, crosslinking, and enzymatic treatment to reduce immunogenicity while preserving the structure and bioactivity of the ECM.

#### 5.1.1 Xenogeneic Decellularized Extracellular Matrix (dECM)

Xenogeneic decellularized extracellular matrix (dECM) is a biomaterial produced by removing cellular components (including cell membranes, nuclei, DNA, cytoplasmic proteins, etc.) from xenogeneic tissues through physical, chemical, or enzymatic methods, while preserving the three‐dimensional structure, biochemical composition, and bioactive molecules of the ECM. The core objective of the decellularization process is to remove immunogenic components while maximally preserving functional components of the ECM, such as collagen fiber networks, glycosaminoglycans, growth factors, and cell adhesion sites. Ideal decellularized ECM should meet the following criteria: residual DNA content <50 ng/mg dry weight, DNA fragment length <200 bp, no visible nuclear remnants, retention of major ECM components (collagen, GAGs, elastin, etc.), and maintenance of original tissue structure and mechanical properties [204].

The advantages of xenogeneic decellularized ECM in osteochondral repair include: (1) Natural three‐dimensional structure: preserves tissue‐specific ECM architecture, providing a natural scaffold for cell adhesion, migration, and differentiation; (2) Bioactive molecule reservoir: contains multiple growth factors (TGF‐β, BMP, FGF, etc.), cytokines, and chemokines that can recruit host cells and regulate tissue regeneration; (3) Low immunogenicity: removal of major immunogenic components after decellularization reduces the risk of rejection reactions; (4) Degradability and tissue integration: ECM can be degraded by host enzymes, releasing bioactive fragments while being gradually replaced by nascent tissue; (5) Wide availability and low cost: can be obtained in large quantities from slaughterhouses or farms, enabling industrial‐scale production.

##### 5.1.1.1 Porcine Small Intestinal Submucosa (SIS)

Small intestinal submucosa (SIS) is a natural ECM material derived from porcine small intestine that has been widely applied in various tissue repair fields due to its abundant bioactive components and excellent tissue‐inductive capacity [205]. SIS retains the composition and structure of native tissue, containing multiple bioactive molecules such as collagen, glycosaminoglycans, and growth factors, providing scaffold support and biochemical signaling for cells.

**FIGURE 6:**
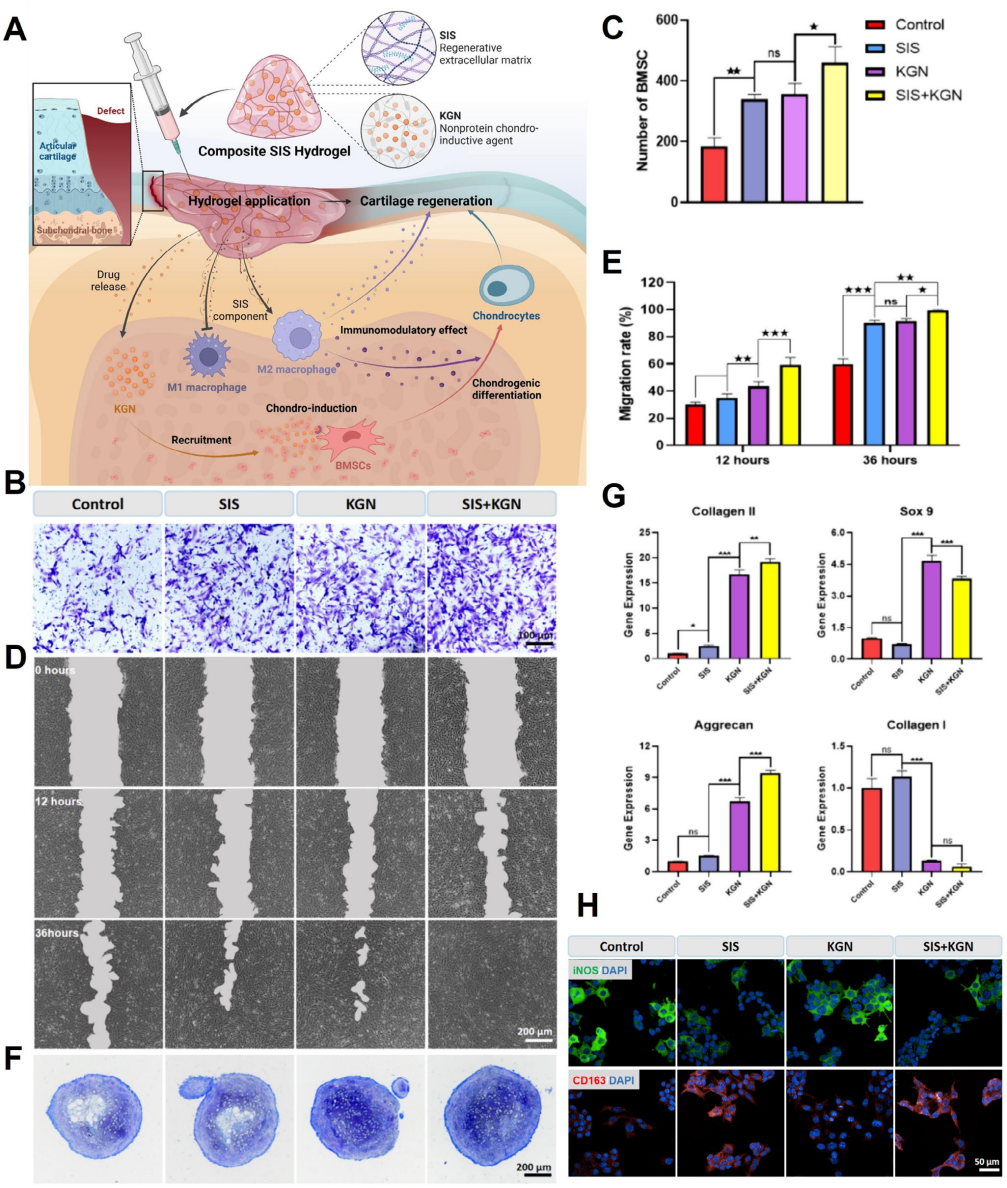
Mechanism and experimental analysis of SIS hydrogel encapsulating KGN for accelerated cartilage regeneration. Schematic illustration (A) demonstrates how SIS hydrogel encapsulating KGN promotes cartilage regeneration through immunomodulation and enhanced chondrogenesis. Transwell assay and its quantitative analysis (B, C) show the impact of SIS hydrogel on cell migration, while scratch assay and corresponding quantitative analysis (D, E) compare the effects of different SIS hydrogels. 3D spheroid culture and Toluidine Blue staining (F) highlight cartilage formation, and the relative expression levels of chondrogenesis‐related genes (G) confirm enhanced chondrogenesis. Immunofluorescence staining of iNOS (M1) and CD163 (M2) (H) further reveals the immunomodulatory effects of SIS hydrogel. Reproduced with permission [13]. Copyright 2024, Elsevier.

To overcome the limitations of the original SIS morphology for cartilage defect treatment, Huang et al. [13] processed SIS into a thermosensitive hydrogel through enzymatic digestion (Figure 6). This hydrogel remains liquid at room temperature, facilitating injection and filling of irregular defects, and rapidly gelates under physiological temperature to form a three‐dimensional porous structure that mimics the microenvironment of native cartilage. This porous structure not only provides necessary scaffold support for progenitor cells during the repair process, but also promotes nutrient diffusion and metabolic waste removal.

A prominent advantage of SIS lies in its exceptional immunomodulatory capacity. Studies have demonstrated that SIS can induce macrophage polarization from pro‐inflammatory M1 phenotype to pro‐regenerative M2 phenotype. M1 macrophages primarily participate in inflammatory responses during the early injury phase, releasing pro‐inflammatory factors and inhibiting mesenchymal stem cell (MSC) differentiation toward chondrocytes; whereas M2 macrophages release anti‐inflammatory factors, exerting protective and promotive effects on chondrogenic differentiation of MSCs [206]. By improving the local immune microenvironment, SIS creates favorable conditions for cartilage regeneration.

To further enhance the therapeutic potential of SIS hydrogel, Huang et al. [13] incorporated the non‐protein chondrogenic agent Kartogenin (KGN) into the SIS hydrogel, constructing an SIS‐KGN composite hydrogel system (Figure 6A). KGN can recruit host endogenous bone marrow mesenchymal stem cells (BMSCs) and induce their differentiation toward chondrocytes, thereby achieving in situ cartilage regeneration without cell transplantation. In vitro experimental systems validated the biological functions of this composite hydrogel. Transwell migration assays demonstrated that SIS‐KGN hydrogel significantly promoted chondrocyte migration, with the number of migrated cells markedly increased compared to the control group (Figure 6B,C). Scratch assays further confirmed that SIS hydrogels at different concentrations all accelerated cell migration and defect healing, with scratch closure rates significantly improved within 24 h (Figure 6D,E). 3D spheroid culture and toluidine blue staining revealed that SIS‐KGN hydrogel promoted cartilage matrix synthesis and proteoglycan deposition, with increased spheroid volume and enhanced staining intensity (Figure 6F). qRT‐PCR analysis showed that the expression levels of chondrogenesis‐related genes (such as COL2A1, ACAN, SOX9) were significantly upregulated (Figure 6G), confirming that KGN can effectively recruit BMSCs and promote their chondrogenic differentiation. Immunofluorescence staining results demonstrated that SIS‐KGN hydrogel could modulate macrophage polarization, with decreased expression of the M1 marker iNOS and increased expression of the M2 marker CD163 (Figure 6H), indicating that this hydrogel indirectly enhanced the chondrogenic differentiation capacity of BMSCs by promoting the M2 anti‐inflammatory phenotype.

In a rabbit knee cartilage defect model, the regenerated tissue in the SIS‐KGN composite hydrogel group closely resembled native hyaline cartilage, exhibiting favorable tissue integration and mechanical properties. Histological analysis revealed that the regenerated cartilage was rich in type II collagen and proteoglycans, with chondrocytes displaying typical rounded morphology and uniform distribution within cartilage lacunae. This result demonstrated that the synergistic effect of SIS hydrogel and KGN can effectively promote cartilage defect repair and regeneration: SIS creates a pro‐regenerative microenvironment through immunomodulation, while KGN achieves cartilage regeneration by recruiting and inducing endogenous stem cells; their combined action accomplishes in situ cartilage repair without exogenous cell transplantation.

As a natural ECM material, SIS provides a simple yet effective therapeutic strategy for cartilage tissue engineering through its unique immunomodulatory function and three‐dimensional scaffold structure, combined with the synergistic action of chondrogenic agents.

##### 5.1.1.2 Decellularized Cartilage Matrix

Decellularized cartilage matrix (DCM) is a decellularized ECM extracted from articular cartilage, costal cartilage, or tracheal cartilage of xenogeneic animals (primarily pigs, cattle, and horses), which preserves the cartilage‐specific ECM composition and structure, making it an ideal scaffold material for cartilage tissue engineering [207]. Compared to dECM from other tissue sources, DCM contains high concentrations of type II collagen (50%–70% of dry weight), proteoglycans (primarily the aggregating proteoglycan aggrecan, 20%–35% of dry weight), cartilage oligomeric matrix protein (COMP), and other cartilage‐specific components, providing a “cartilage‐like” microenvironment for chondrocytes or stem cells that promotes cartilage phenotype maintenance and matrix synthesis [208].

The dense structure and avascular nature of cartilage make its decellularization process more challenging than other tissues [209]. The high density and negative charge characteristics of cartilage ECM limit the penetration of decellularization reagents, requiring longer processing times and more stringent decellularization conditions. Commonly used decellularization methods include: high‐concentration SDS treatment (0.5%–1% SDS, 3‐7 days) can effectively remove cells but may lead to substantial GAG loss (up to 50%–80%); Triton X‐100 combined with hypertonic saline solution is milder with higher GAG retention rates (70%–90%), but slightly lower decellularization efficiency; freeze‐thaw cycles combined with enzymatic treatment disrupt cell membranes through ice crystal formation, followed by nuclease and trypsin treatment to remove cellular remnants, achieving effective decellularization while preserving ECM components.

DCM has diverse application forms in osteochondral repair. DCM particles or powder (50–500 µm) possess a high specific surface area and can be mixed with cells and growth factors to fabricate injectable composite scaffolds. Rowland et al. [178] demonstrated that DCM particles loaded with BMSCs for cartilage defect repair resulted in type II collagen content in regenerated tissue 2.5 times higher than the BMSCs‐only group at 12 weeks. DCM hydrogels are produced through enzymatic digestion and can self‐assemble into fibrous networks under physiological conditions, retaining the biochemical signals of cartilage ECM and inducing MSC differentiation toward chondrocytes with 5–10 fold increases in cartilage‐related gene expression. Bulk DCM scaffolds preserve the layered structure and mechanical anisotropy of native cartilage, but cell infiltration is challenging and typically requires freeze–drying or pore‐forming techniques to increase porosity. DCM composite scaffolds combine DCM with synthetic materials (such as PCL, PLGA) or natural polymers (such as chitosan), integrating the advantages of each material and demonstrating excellent stratified regeneration effects in osteochondral defect repair.

DCM has diverse application forms in osteochondral repair. DCM particles or powder (50–500 µm) possess a high specific surface area and can be mixed with cells and growth factors to fabricate injectable composite scaffolds. Rowland et al. [178] demonstrated that DCM particles loaded with BMSCs for cartilage defect repair resulted in type II collagen content in regenerated tissue 2.5 times higher than the BMSCs‐only group at 12 weeks. DCM hydrogels are produced through enzymatic digestion and can self‐assemble into fibrous networks under physiological conditions, retaining the biochemical signals of cartilage ECM and inducing MSC differentiation toward chondrocytes with 5–10 fold increases in cartilage‐related gene expression. Bulk DCM scaffolds preserve the layered structure and mechanical anisotropy of native cartilage, but cell infiltration is challenging and typically requires freeze–drying or pore‐forming techniques to increase porosity. DCM composite scaffolds combine DCM with synthetic materials (such as PCL, PLGA) or natural polymers (such as chitosan), integrating the advantages of each material and demonstrating excellent stratified regeneration effects in osteochondral defect repair.

##### 5.1.1.3 Decellularized Bone Matrix

Decellularized bone matrix (DBM) is an ECM material obtained by removing cells and partial minerals from xenogeneic animal bone tissue, primarily composed of type I collagen (>90% of organic components), non‐collagenous proteins (osteocalcin, osteopontin, bone sialoprotein, etc.), and residual hydroxyapatite [210]. DBM retains the osteoinductive capacity of bone tissue and contains multiple bone morphogenetic proteins (BMPs), making it an important material for bone tissue engineering and osteochondral interface repair [211].

Decellularization of bone tissue is relatively straightforward due to the porous structure of bone, which facilitates reagent penetration. Commonly employed methods include physical approaches such as high pressure, ultrasound, and freeze‐thaw cycles; chemical treatments using low‐concentration SDS, Triton X‐100, or hydrogen peroxide; and enzymatic digestion with trypsin or nucleases. Following decellularization, partial demineralization treatment with EDTA or dilute hydrochloric acid is typically performed to expose collagen fibers and growth factors, thereby enhancing bioactivity.

Ci et al. [192] developed a biomimetic stratified scaffold based on DBM for osteochondral repair. This scaffold was fabricated through gradient demineralization treatment: the bone layer retained 80%–90% minerals, the intermediate transition layer retained 40%–60% minerals, and the cartilage layer was completely demineralized. The scaffold surface was grafted with hyaluronic acid through dopamine modification to improve hydrophilicity and cell adhesion. Mechanical testing showed that the bone layer had a compressive modulus of 150 MPa, approaching that of cancellous bone (100–200 MPa), while the cartilage layer had a compressive modulus of 1.8 MPa, approaching that of native cartilage (0.5–2 MPa). In a rabbit knee osteochondral defect model, the stratified DBM scaffold group achieved synchronized regeneration of cartilage and subchondral bone at 12 weeks, with calcified cartilage layer formation in the interface region, accomplishing a natural cartilage‐to‐bone transition [212].

**FIGURE 7:**
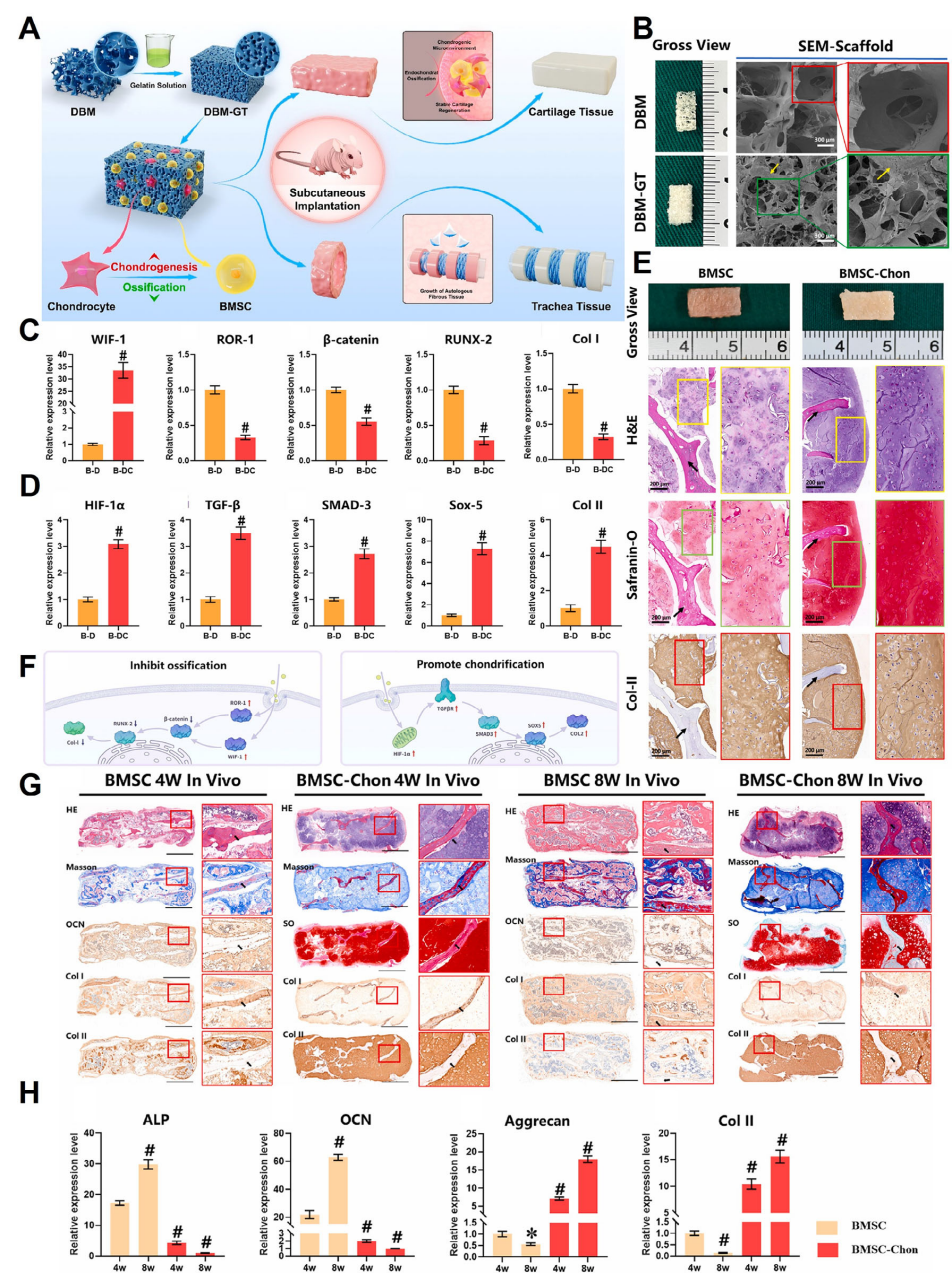
Microstructural modification of decalcified bone matrix (DBM) scaffolds and co‐culture of bone marrow stem cells (BMSCs) with chondrocytes to promote stable cartilage regeneration. (A) Schematic illustration showing how microstructurally modified DBM scaffolds and BMSC‐chondrocyte co‐culture promote stable cartilage regeneration. (B) Overall morphology and scanning electron microscopy (SEM) images of DBM and DBM‐GT scaffolds. (C) RT‐PCR analysis of osteogenesis‐related gene expression. (D) RT‐PCR analysis of chondrogenesis‐related gene expression. (E) Macroscopic appearance and histological results (H&E, Safranin O, and Col II staining) of regenerated cartilage after 4 weeks of culture in chondrogenic medium (CM) in different groups. (F) Schematic illustration showing the inhibition of osteogenic differentiation and promotion of chondrogenesis. (G) Histological staining (H&E, Masson, OCN, Col I, and Col II) of regenerated tissues from BMSC and BMSC‐Chon groups after 4 and 8 weeks of implantation. Scale bar = 1.25 mm. (H) Expression levels of bone‐related genes (ALP, OCN) and cartilage‐related genes (Aggrecan, Col II) in regenerated tissues after 4 and 8 weeks of implantation. *: *p* < 0.05, #: *p* < 0.01. Reproduced with permission [192]. Copyright 2025, Elsevier.

To further optimize the cartilage regeneration capacity of DBM scaffolds and inhibit unnecessary osteogenic differentiation, researchers developed microstructure‐modified decalcified bone matrix scaffolds (DBM‐GT) combined with a bone marrow stem cell‐chondrocyte co‐culture strategy (Figure 7A). Scanning electron microscopy revealed that DBM‐GT scaffolds modified with gelatin‐transglutaminase formed a more uniform microporous structure on the surface (Figure 7B). In vitro gene expression analysis demonstrated that DBM‐GT scaffolds significantly suppressed the expression of osteogenesis‐related genes (ALP, OCN) (Figure 7C), while significantly upregulating the expression of chondrogenesis‐related genes (aggrecan, type II collagen) (Figure 7D). After 4 weeks of culture under chondrogenic conditions, the BMSC‐chondrocyte co‐culture group formed denser cartilage tissue, with safranin O staining showing abundant proteoglycan deposition and type II collagen immunohistochemistry displaying strong positivity (Figure 7E,F). In vivo experiments showed that at 4 and 8 weeks post‐implantation, the BMSC‐Chon group regenerated tissue exhibited typical cartilage tissue structure, with negative osteocalcin staining indicating effective inhibition of osteogenic differentiation and strongly positive type II collagen staining confirming the regenerated tissue as hyaline cartilage (Figure 7G). Gene expression analysis further confirmed that the BMSC‐Chon group showed significantly decreased bone‐related gene expression and significantly increased cartilage‐related gene expression (Figure 7H).

The advantages of DBM in osteochondral repair include osteoinductive capacity, osteoconductivity, tunable mineralization degree, and favorable tissue integration. Application forms of DBM include DBM particles, DBM strips or blocks, DBM gel or putty, and DBM composite scaffolds [213]. However, DBM also has limitations such as large batch‐to‐batch variability, unstable growth factor content, immunogenicity risks, and a mismatch between degradation rate and bone regeneration.

##### 5.1.1.4 Other Xenogeneic Decellularized Matrices

In addition to commonly used porcine and bovine decellularized matrices, researchers have explored various other xenogeneic ECM materials for cartilage and osteochondral repair, which demonstrate potential application value due to their unique compositional and structural characteristics.

Marine organism‐derived ECM has become a research hotspot in recent years. Decellularized squid cartilage (DSC) has attracted attention due to its abundance in type II collagen and proteoglycans, and its biochemical composition is similar to mammalian cartilage [214]. The advantages of DSC lie in its low immunogenicity and favorable biocompatibility; decellularization treatment can effectively remove xenoantigens while preserving cartilage‐specific ECM components. Studies have shown that DSC scaffolds can support chondrocyte adhesion, proliferation, and matrix synthesis, promoting hyaline cartilage‐like tissue formation. Additionally, marine biological tissues such as fish scales and sea cucumber body walls have also been developed as decellularized matrix materials, with their abundant collagen and chondroitin sulfate providing bioactive support for cartilage repair [215].

Avian‐derived ECM also shows application potential. Decellularized chicken sternal cartilage has been investigated for cartilage tissue engineering due to its easy accessibility, low cost, and abundance in cartilage‐specific components. Eggshell membrane (ESM) contains components such as type I and type X collagen, hyaluronic acid, and chondroitin sulfate, possessing the capacity to promote chondrocyte differentiation and matrix synthesis [12].

Other mammalian‐derived ECM includes equine, ovine, and lapine decellularized matrices, which are selected in certain regions due to resource accessibility and cultural acceptance. Decellularized deer antler matrix demonstrates unique advantages in promoting cartilage regeneration due to its abundance in growth factors and cartilage progenitor cell microenvironment components [216].

Common challenges for these xenogeneic decellularized matrices include decellularization efficiency, immunogenicity control, batch‐to‐batch consistency, and standardization of functional validation. Nevertheless, the diversified xenogeneic ECM sources provide more options for cartilage tissue engineering, particularly possessing practical application value under specific cultural contexts or resource‐limited conditions.

#### 5.1.2 Animal‐Derived Collagen

##### 5.1.2.1 Type I Collagen

Type I collagen, the most abundant collagen in mammals, accounts for approximately 90% of total body collagen and serves as the principal structural protein in bone, tendon, ligament, skin, and cornea. In bone tissue, type I collagen constitutes over 90% of the organic matrix and, in conjunction with hydroxyapatite minerals, forms the composite architecture of bone, conferring tensile strength and toughness. In osteochondral tissue engineering, type I collagen represents the material of choice for bone layer scaffold construction owing to its exceptional biocompatibility, biodegradability, and cell adhesion properties. The type I collagen molecule comprises two α1(I) chains and one α2(I) chain assembled into a triple helical structure with a molecular weight of approximately 300 kDa. Each α chain consists of Gly‐X‐Y repeating sequences, where positions X and Y are frequently occupied by proline and hydroxyproline [217]. Collagen molecules self‐assemble into fibers with diameters of 50–200 nm, exhibiting characteristic 67 nm periodic banding. Type I collagen contains abundant cell‐binding motifs, including RGD and GFOGER sequences, which mediate cell adhesion through integrins and promote osteoblast attachment, proliferation, and differentiation [218].

Animal‐derived type I collagen is primarily extracted from bovine, porcine, and piscine tissues. Bovine collagen exhibits high purity and favorable mechanical properties but carries potential immunogenicity and disease transmission risks. Porcine collagen demonstrates similar performance, though religious and cultural considerations limit its application. Fish‐derived collagen has garnered attention due to its low immunogenicity and high acceptability, although its relatively low denaturation temperature (approximately 15°C–25°C) results in comparatively weaker mechanical properties. Extraction methods include acid and enzymatic approaches, followed by purification, lyophilization, and crosslinking to fabricate scaffold materials [219]. Type I collagen finds application in bone layer scaffolds in multiple forms: collagen sponges with high porosity (>90%) and pore sizes of 50–300 µm facilitate cell migration and vascular ingrowth; collagen membranes with enhanced mechanical strength are employed for guided bone regeneration; and injectable collagen hydrogels are suitable for filling irregular defects [220]. Physical, chemical, or enzymatic crosslinking is commonly employed to improve mechanical properties and modulate degradation kinetics.

Type I collagen is frequently composited with hydroxyapatite (HA) to recapitulate native bone architecture. Collagen‐HA composites are fabricated through biomimetic mineralization, coprecipitation, or physical blending, with optimal mass ratios ranging from 70:30 to 50:50. Nano‐hydroxyapatite (nHA) demonstrates superior performance due to its high specific surface area and enhanced bioactivity [221]. Several collagen‐HA composite products, including Healos and Collagraft, have received clinical approval and demonstrated efficacy in promoting bone defect repair. Type I collagen scaffolds facilitate bone regeneration through multiple mechanisms: providing cell adhesion sites and activating FAK, ERK, and Akt signaling pathways; generating degradation products that serve as building blocks for bone formation and activate specific receptors such as DDR2; and adsorbing and sustaining the release of growth factors, including BMP‐2 and VEGF. In bilayer osteochondral scaffolds, the type I collagen bone layer provides mechanical support, promotes vascularization and osseointegration, thereby establishing a stable foundation for overlying cartilage regeneration.

Despite widespread application, type I collagen scaffolds face several challenges: limited mechanical strength (compressive modulus <1 MPa versus 10–20 GPa for native bone), rapid degradation (half‐life of 2–4 weeks), batch‐to‐batch variability, and potential immunogenicity [222]. Future advances will likely focus on recombinant human type I collagen to eliminate immunogenicity, biomimetic scaffolds with gradient porosity and hierarchically aligned architectures that recapitulate native tissue organization, and 3D bioprinting technologies for patient‐specific fabrication with precise spatial control.

##### 5.1.2.2 Type II Collagen

Type II collagen is the major structural protein of hyaline cartilage ECM, accounting for 90%–95% of total cartilage collagen, and serves as the core component for maintaining structural integrity and biomechanical function of cartilage tissue. Unlike type I collagen, which is primarily distributed in tissues such as bone and tendon, type II collagen is almost exclusively expressed in cartilage tissue; its unique molecular structure and biological functions make it a key material for maintaining hyaline cartilage phenotype and avoiding fibrosis and hypertrophy in cartilage tissue engineering. In osteochondral repair, type II collagen scaffolds can provide tissue‐specific microenvironments for chondrocytes, promoting deposition of cartilage‐characteristic matrix (type II collagen and proteoglycans), achieving hyaline cartilage regeneration rather than fibrocartilage substitution [223].

The type II collagen molecule consists of three identical α1(II) chains forming a homotrimeric structure, with a molecular weight of approximately 300 kDa. Compared to type I collagen, type II collagen possesses higher hydroxylysine content and glycosylation levels, enabling it to form tighter interaction networks with proteoglycans. Type II collagen fibers have smaller diameters (typically 10–20 nm) and display a randomly interwoven network structure; this structure confers cartilage tissue the ability to resist compressive stress. Type II collagen contains chondrocyte‐specific binding sites that mediate cell adhesion through integrins α1β1, α2β1, and α10β1, activating downstream signaling pathways (such as PI3K/Akt and MAPK pathways) and maintaining the rounded morphology of chondrocytes and cartilage phenotype gene expression (SOX9, COL2A1, ACAN). Studies have shown that chondrocytes cultured on type II collagen matrices can maintain the cartilage phenotype long‐term, whereas on type I collagen or plastic culture dishes, they rapidly dedifferentiate into fibroblast‐like cells [129].

Animal‐derived type II collagen is primarily extracted from bovine, porcine, and chicken articular cartilage or sternal cartilage, with chicken sternal cartilage being the most commonly used source due to its high type II collagen content (approximately 50% of dry weight) and ease of large‐scale procurement [224]. Compared to type I collagen, the extraction of type II collagen is more challenging because cartilage tissue is dense with high proteoglycan content, requiring more stringent purification conditions to remove impurities. Type II collagen has diverse application forms in cartilage layer scaffolds: collagen sponges are fabricated by lyophilization with porosity of 85‐95% and pore size of 50‐200 µm, suitable for three‐dimensional chondrocyte culture and matrix secretion; collagen hydrogels are formed through neutralization and temperature‐controlled induced fibrillation, possessing injectability and in situ gelation properties, suitable for minimally invasive treatment and irregular defect filling; collagen membranes are prepared by compression or electrospinning and can be used to cover defect surfaces or serve as substrates for cell sheet engineering [225]. To improve mechanical properties and degradation rate, physical crosslinking (dehydrothermal treatment, UV irradiation), chemical crosslinking (genipin, carbodiimide, transglutaminase), or photocrosslinking (methacrylated collagen) are commonly employed. Chemical crosslinking can significantly enhance compressive modulus and resistance to collagenase degradation, but the crosslinking degree must be controlled to avoid cytotoxicity and reduced bioactivity.

**FIGURE 8:**
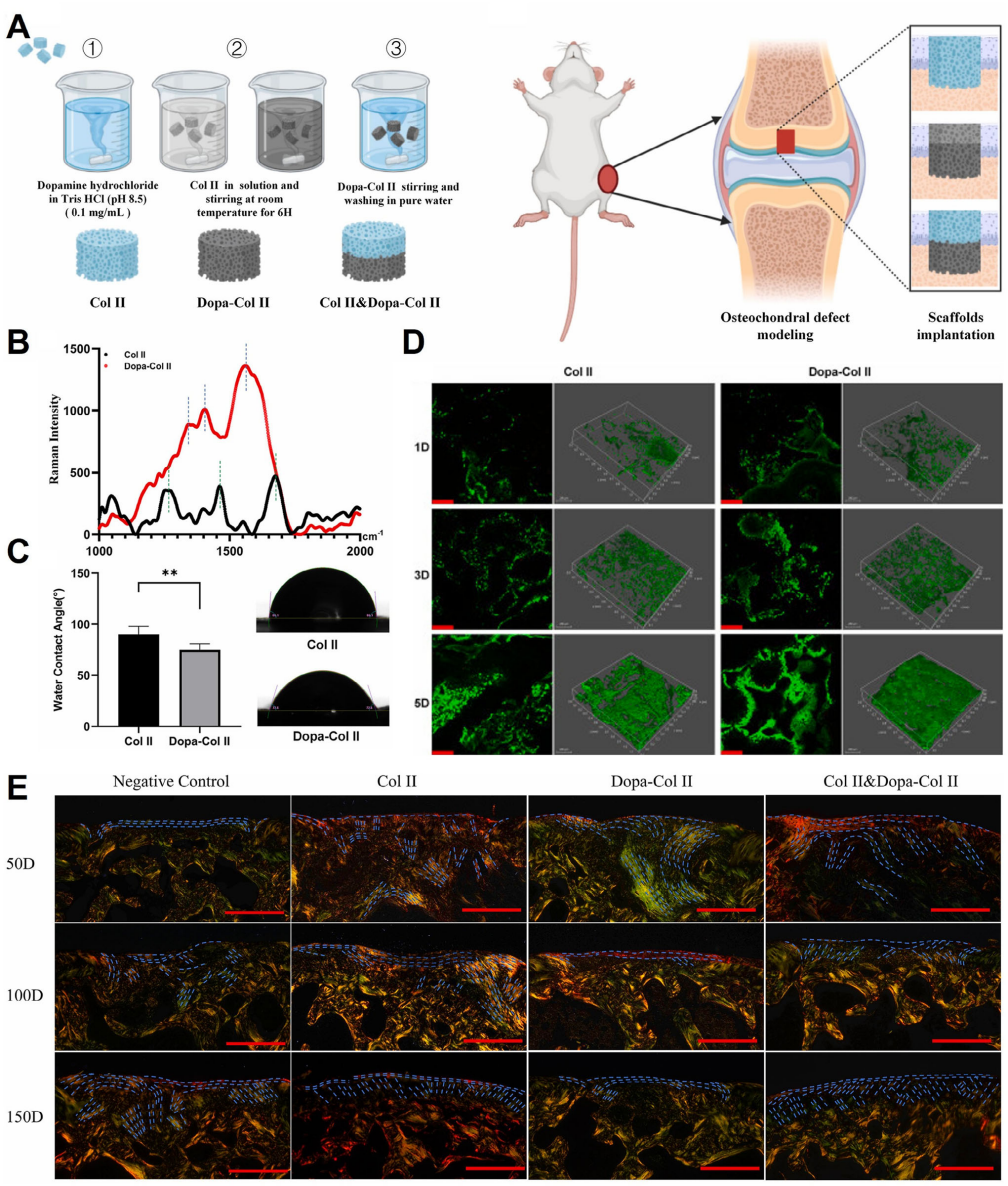
Scaffold construction steps and characterization, along with implantation in an osteochondral defect model. (A) Schematic illustration of the scaffold fabrication process and the implantation procedure in an osteochondral defect model. (B) Raman spectra of the surfaces of different scaffolds, demonstrating their compositional characteristics. (C) Water contact angles of different scaffolds (*n* = 3), indicating their hydrophilicity. (D) Live/dead cell staining of chondrocytes seeded on different scaffolds at days 1, 3, and 5 (live cells: green, dead cells: red; scale bar = 200 µm). (E) Sirius Red staining to analyze the alignment of collagen fibers, with blue dashed lines indicating the fitted fiber orientation. Scale bar: 500 µm. Reproduced under the terms of the CC BY‐NC‐ND 4.0 license [193]. Copyright 2025, The Author(s).

Zhang et al. [193] developed a type II collagen bilayer scaffold (Col II & Dopa‐Col II) that provides an innovative strategy for osteochondral repair. The upper layer of the scaffold is pure type II collagen for hyaline cartilage regeneration; the lower layer is polydopamine (PDA)‐coated type II collagen for promoting endochondral ossification and vascularized subchondral bone regeneration (Figure 8A). Raman spectroscopy analysis revealed a characteristic peak at 1580 cm^−^^1^ for the PDA coating, confirming successful coating modification (Figure 8B). Water contact angle testing demonstrated that the PDA coating significantly reduced the contact angle of the scaffold, enhancing hydrophilicity and cell affinity (Figure 8C). Live/dead cell staining showed that chondrocytes exhibited favorable viability (>95%) on both scaffolds, with cell density significantly increasing at day 5, indicating excellent biocompatibility of the scaffolds (Figure 8D). Sirius red staining combined with polarized light microscopy analysis revealed that collagen fibers in the Col II scaffold displayed a randomly interwoven network structure with uniform fiber orientation distribution; this structure is favorable for resisting multidirectional compressive stress (Figure 8E).

In a rabbit knee osteochondral defect model, the Col II & Dopa‐Col II bilayer scaffold achieved synchronous regeneration of hyaline cartilage and vascularized subchondral bone at 12 weeks. Histological scoring (ICRS II score) demonstrated that the Col II & Dopa‐Col II group was significantly superior to the Col II‐only group and blank control group, with regenerated cartilage exhibiting smooth surfaces, uniform cell distribution, strongly positive matrix staining, intact subchondral bone structure, regularly arranged bone trabeculae, and high vascular density. Immunohistochemical staining revealed abundant type II collagen expression with minimal type I collagen expression in the regenerated cartilage, confirming maintenance of the hyaline cartilage phenotype; dense distribution of type X collagen and CD31‐positive vessels in the subchondral bone region confirmed successful achievement of endochondral ossification and vascularization. Biomechanical testing showed that the compressive modulus and hardness of the Col II & Dopa‐Col II group approached those of native osteochondral tissue, significantly higher than other control groups.

Type II collagen scaffolds have made progress in clinical translation. Multiple type II collagen‐based products have been approved or entered clinical trials, such as Chondro‐Gide (porcine‐derived type II collagen membrane for MACI surgery), NOVOCART 3D (type II collagen scaffold combined with autologous chondrocytes), and BioCartilage (micronized cartilage matrix enriched with type II collagen). Clinical studies have shown that these products can significantly improve cartilage defect repair outcomes, with regenerated tissue exhibiting superior histological and biomechanical properties compared to traditional microfracture surgery.

However, type II collagen scaffolds still face challenges: the mechanical strength of natural type II collagen (compressive modulus typically 50–200 kPa) is far lower than that of native cartilage (0.5–1 MPa), limiting its application in load‐bearing sites [226]; the immunogenicity and batch‐to‐batch variability of animal‐derived type II collagen require stringent quality control; the degradation rate of scaffolds needs to be precisely matched with the rate of new tissue formation—excessively rapid degradation may lead to structural collapse, while excessively slow degradation hinders tissue remodeling.

##### 5.1.2.3 Gelatin

Gelatin is a product of partial hydrolysis and thermal denaturation of collagen protein following acid, alkali, or enzymatic treatment, losing the triple‐helical structure of collagen and presenting as randomly coiled single‐chain or double‐chain polypeptides. Gelatin retains the major amino acid composition of collagen (rich in glycine, proline, and hydroxyproline) and cell‐binding motifs (such as RGD sequences), but due to structural denaturation possesses better water solubility, processability, and lower immunogenicity. According to different extraction methods, gelatin is classified into Type A (acid extraction, isoelectric point pH 7–9) and Type B (alkali extraction, isoelectric point pH 4.5–5.5), with differences in gel strength, dissolution temperature, and cell interactions between the two types [223].

In osteochondral tissue engineering, gelatin is commonly used as a substitute material for collagen or as a composite component. Gelatin hydrogels are formed through temperature regulation or chemical crosslinking (such as glutaraldehyde, transglutaminase), possessing injectability and favorable cell encapsulation capacity, suitable for loading cells and growth factors. Methacrylated gelatin (GelMA) is the most commonly used photocrosslinkable gelatin derivative, rapidly forming gels upon UV irradiation with a tunable crosslinking degree, widely applied in 3D bioprinting and minimally invasive injection therapy. Gelatin sponges and gelatin microspheres are commonly used for drug sustained release and hemostatic materials [129].

The advantages of gelatin scaffolds lie in low cost, wide availability, good biocompatibility, and degradation products that can be absorbed and utilized by the body [220]. However, the mechanical strength of gelatin is far lower than that of native collagen (compressive modulus typically <10 kPa), rapid degradation (in vivo half‐life of days to weeks), and lower thermal stability (gelation temperature approximately 25°C–35°C) limit its standalone application. Therefore, gelatin is often used in combination with other materials, such as gelatin‐chitosan, gelatin‐hydroxyapatite, gelatin‐polycaprolactone composite scaffolds, improving mechanical properties and bioactivity through synergistic effects. In osteochondral bilayer scaffolds, gelatin can serve as a transition layer or growth factor carrier, promoting interface integration and signaling molecule delivery [227].

##### 5.1.2.4 Collagen Peptides

Collagen peptides are small molecular polypeptide fragments generated through enzymatic or chemical hydrolysis of collagen protein, typically with molecular weights of 2–10 kDa, composed of several to dozens of amino acid residues [228]. Compared to intact collagen protein, collagen peptides possess higher water solubility, bioavailability, and tissue permeability, and lack immunogenicity and antigenicity. Collagen peptides retain the characteristic amino acid sequences of collagen (such as Gly‐Pro‐Hyp, Gly‐Pro‐Ala), can be directly absorbed by the intestine, and are targeted for distribution to cartilage and bone tissues [229].

Collagen peptides primarily exert bioactive functions rather than structural support roles in osteochondral repair. Studies have shown that collagen peptides can stimulate chondrocyte and osteoblast proliferation, promote the synthesis of type II collagen, proteoglycans, and type I collagen, and upregulate expression of key genes such as SOX9, COL2A1, RUNX2, and OCN. Collagen peptides also possess anti‐inflammatory and antioxidant activities, capable of inhibiting IL‐1β and TNF‐α‐induced cartilage degradation, downregulating MMP‐1, MMP‐13, and ADAMTS‐5 expression, and delaying osteoarthritis progression. Additionally, collagen peptides can promote angiogenesis and callus formation, accelerating fracture healing [230].

In tissue engineering applications, collagen peptides are commonly used as bioactive additives incorporated into scaffold materials or hydrogels, continuously released through sustained‐release systems to maintain local high concentrations. Collagen peptides can also be immobilized on scaffold surfaces through surface modification (such as grafting, covalent coupling), enhancing cell adhesion and differentiation. Oral collagen peptide supplements are clinically used to improve joint function and relieve pain, with multiple randomized controlled trials demonstrating certain efficacy in osteoarthritis patients [231]. However, the mechanisms of action of collagen peptides have not been fully elucidated, and their optimal molecular weight range, amino acid sequences, and administration routes still require further investigation.

##### 5.1.2.5 Other ECM Proteins

In addition to collagen proteins, other structural proteins in the ECM also play important roles in osteochondral tissue engineering. Fibronectin (FN) is a high molecular weight glycoprotein (approximately 440 kDa) that binds to integrins α5β1 and αvβ3 through RGD sequences and PHSRN synergy sites, mediating cell adhesion, spreading, and migration. Fibronectin can promote initial attachment and chondrogenic differentiation of mesenchymal stem cells on scaffold surfaces, commonly modifying biomaterials through surface coating or covalent grafting methods, significantly improving cell seeding efficiency and distribution uniformity. Studies have shown that fibronectin‐modified scaffolds can upregulate integrin signaling pathways, enhance mechanical transduction at the cell‐material interface, and promote cartilage‐specific gene expression [232].

Laminin is a major component of the basement membrane, a cross‐shaped glycoprotein (approximately 400–900 kDa) composed of α, β, and γ chains, containing multiple cell‐binding domains (such as IKVAV and YIGSR sequences). Laminin promotes cell migration, neurite outgrowth, and angiogenesis through integrin α6β1 and dystroglycan receptors, playing an important role in vascularization and innervation of the osteochondral interface. Laminin coating or peptide sequence modification can guide stem cell migration toward the subchondral bone region, promoting bone‐cartilage interface integration. However, laminin is expensive and difficult to extract, limiting its large‐scale application, with synthetic peptide fragments (such as IKVAV peptide) becoming economical alternatives [233].

Fibrin is the terminal product of the coagulation cascade, formed by polymerization of fibrinogen under the action of thrombin into a three‐dimensional network structure. Fibrin gels possess excellent injectability and in situ gelation capability, with gelation time precisely controllable (from seconds to minutes) by adjusting thrombin and calcium ion concentrations, widely used for minimally invasive cell delivery and growth factor sustained release. Fibrin contains multiple cell‐binding sites and matrix metalloproteinase (MMP) degradation sites, supporting cell invasion, remodeling, and new tissue formation. In osteochondral repair, fibrin gels are commonly used as cell carriers (such as MSCs, chondrocytes) or in combination with other scaffold materials, with clinical products such as Tisseel and Evicel approved for surgical hemostasis and tissue adhesion [194]. However, the rapid degradation of fibrin (in vivo half‐life approximately 1–2 weeks) and lower mechanical strength (compressive modulus <1 kPa) limit its standalone application, often requiring chemical crosslinking (such as factor XIIIa, transglutaminase) or compositing with polymers to extend degradation time and enhance mechanical properties.

#### 5.1.3 Animal‐Derived Polysaccharides

Polysaccharides are natural macromolecular polymers formed by monosaccharides linked through glycosidic bonds, widely present in animal and plant tissues. Animal‐derived polysaccharides play important roles in cartilage and osteochondral tissue engineering due to their good biocompatibility, biodegradability, and bioactivity. Compared to synthetic polymers, animal‐derived polysaccharides possess the structural and functional characteristics of natural ECM components, capable of better mimicking the cartilage tissue microenvironment and promoting cell adhesion, proliferation, and cartilage‐specific differentiation. This section focuses on three animal‐derived polysaccharides most widely applied in cartilage repair: chitosan, chondroitin sulfate, and hyaluronic acid.

##### 5.1.3.1 Chitosan

Chitosan is a natural cationic polysaccharide prepared by deacetylation of chitin, primarily sourced from the exoskeletons of crustaceans such as shrimp and crabs, and can also be extracted from fungal cell walls. The chemical structure of chitosan is a linear copolymer of β‐(1→4)‐linked D‐glucosamine and N‐acetyl‐D‐glucosamine, with a degree of deacetylation (DD) typically between 60%‐100%, which determines the material's solubility, degradation rate, and biological properties. The cationic nature of chitosan enables it to carry positive charges at physiological pH, allowing electrostatic interactions with negatively charged cell membranes, proteins, and nucleic acids, conferring good biocompatibility, biodegradability, antibacterial properties, hemostatic properties, and mucoadhesive properties. In vivo, chitosan is primarily degraded by lysozyme into non‐toxic oligosaccharides and monosaccharides, ultimately metabolized into carbon dioxide and water, with a degradation rate closely related to molecular weight, degree of deacetylation, and crystallinity [234].

Chitosan provides good support for chondrocytes, promoting chondrocyte adhesion, proliferation, and phenotype maintenance. Studies have shown that chitosan matrices can upregulate expression of cartilage‐specific markers such as type II collagen, aggrecan, and Sox9, while inhibiting expression of type I collagen and type X collagen, preventing chondrocyte dedifferentiation and hypertrophy. Chitosan also possesses immunomodulatory effects, capable of regulating macrophage polarization, promoting M2‐type anti‐inflammatory phenotype, and reducing joint inflammatory responses. Chitosan can be processed into various forms for cartilage repair, including porous scaffolds, hydrogels, microspheres, nanofibers, and composite materials. Chitosan porous scaffolds are typically prepared through freeze–drying, phase separation, or porogen leaching methods, with porosity reaching 80%–95% and pore size ranging from 50–300 µm, suitable for cell migration and nutrient diffusion. However, pure chitosan scaffolds have relatively low mechanical strength (compressive modulus typically <1 MPa), making it difficult to meet the demands of load‐bearing cartilage repair [235].

To overcome the limitation of insufficient mechanical properties, researchers have developed various chitosan‐based composite materials. Chitosan‐collagen composite scaffolds combine the antibacterial properties of chitosan and the cell adhesion properties of collagen, demonstrating superior cartilage repair effects compared to single materials. Chitosan‐hydroxyapatite composite materials are suitable for osteochondral interface repair, and chitosan‐polycaprolactone (PCL) composite nanofiber scaffolds prepared by electrospinning can achieve compressive modulus of 5–10 MPa. Chemical modification of chitosan can further enhance its properties: sulfated chitosan can bind growth factors (such as TGF‐β, BMP‐2), enhancing chondrogenic induction capability; carboxymethyl chitosan possesses better water solubility and has been developed as injectable hydrogels; chitosan surface grafting with RGD peptides can significantly improve cell adhesion and spreading. The cationic nature of chitosan makes it an ideal drug carrier, capable of encapsulating growth factors, small molecule drugs, and gene therapy vectors, achieving controlled release and targeted delivery [236].

Chitosan‐based materials have been applied in clinical cartilage repair. BST‐CarGel (chitosan‐blood implant) is the first chitosan product approved in Canada and Europe for cartilage defect repair, with clinical studies showing it can improve the quality of cartilage repair in microfracture surgery [237]. However, chitosan applications still face challenges, including batch‐to‐batch variability, insufficient mechanical properties, degradation rate control, and potential immunogenicity.

##### 5.1.3.2 Chondroitin Sulfate

Chondroitin sulfate (CS) is a sulfated glycosaminoglycan (GAG) formed by alternating linkages of D‐glucuronic acid and N‐acetylgalactosamine through β‐1,3 and β‐1,4 glycosidic bonds, accounting for approximately 3%–6% of cartilage dry weight, primarily existing as proteoglycans in the cartilage ECM [238]. CS is mainly extracted from animal cartilage tissues such as bovine tracheal cartilage, porcine nasal cartilage, chicken sternal cartilage, and shark cartilage. Based on different sulfation positions, CS is classified into isomers, including CS‐A (4‐sulfated), CS‐C (6‐sulfated), CS‐D (2,6‐disulfated), and CS‐E (4,6‐disulfated), with these structural differences affecting their interactions with growth factors and cell receptors [239].

CS performs multiple functions in cartilage: as aggrecan side chains, it interacts with the collagen fiber network to maintain cartilage structural integrity; its negative charges attract water molecules to form a highly hydrated gel‐like matrix, conferring compressive resistance to cartilage; it binds and stabilizes growth factors such as TGF‐β, FGF, and BMP, regulating signal transduction; it reduces production of pro‐inflammatory factors such as IL‐1β and TNF‐α by inhibiting the NF‐κB signaling pathway; it regulates chondrocyte proliferation, differentiation, and matrix synthesis through receptors such as CD44 and RHAMM [240].

Chondroitin sulfate is commonly combined with type I or type II collagen for cartilage tissue engineering to mimic natural cartilage ECM. Collagen‐chondroitin sulfate (Col‐CS) composite scaffolds are prepared through freeze–drying, electrospinning, and other methods, possessing high porosity (80%–95%) and interconnected porous structures [195]. The addition of CS increases interfibrillar crosslinking through electrostatic interactions, enhancing scaffold compressive modulus and enzymatic degradation resistance, and upregulating type II collagen and aggrecan expression in chondrocytes. Clinically, MACI uses type I/III collagen‐CS membranes as autologous chondrocyte carriers, widely applied in cartilage defect repair; CaReS employs type I collagen gel combined with CS to promote hyaline cartilage‐like tissue regeneration [241].

**FIGURE 9:**
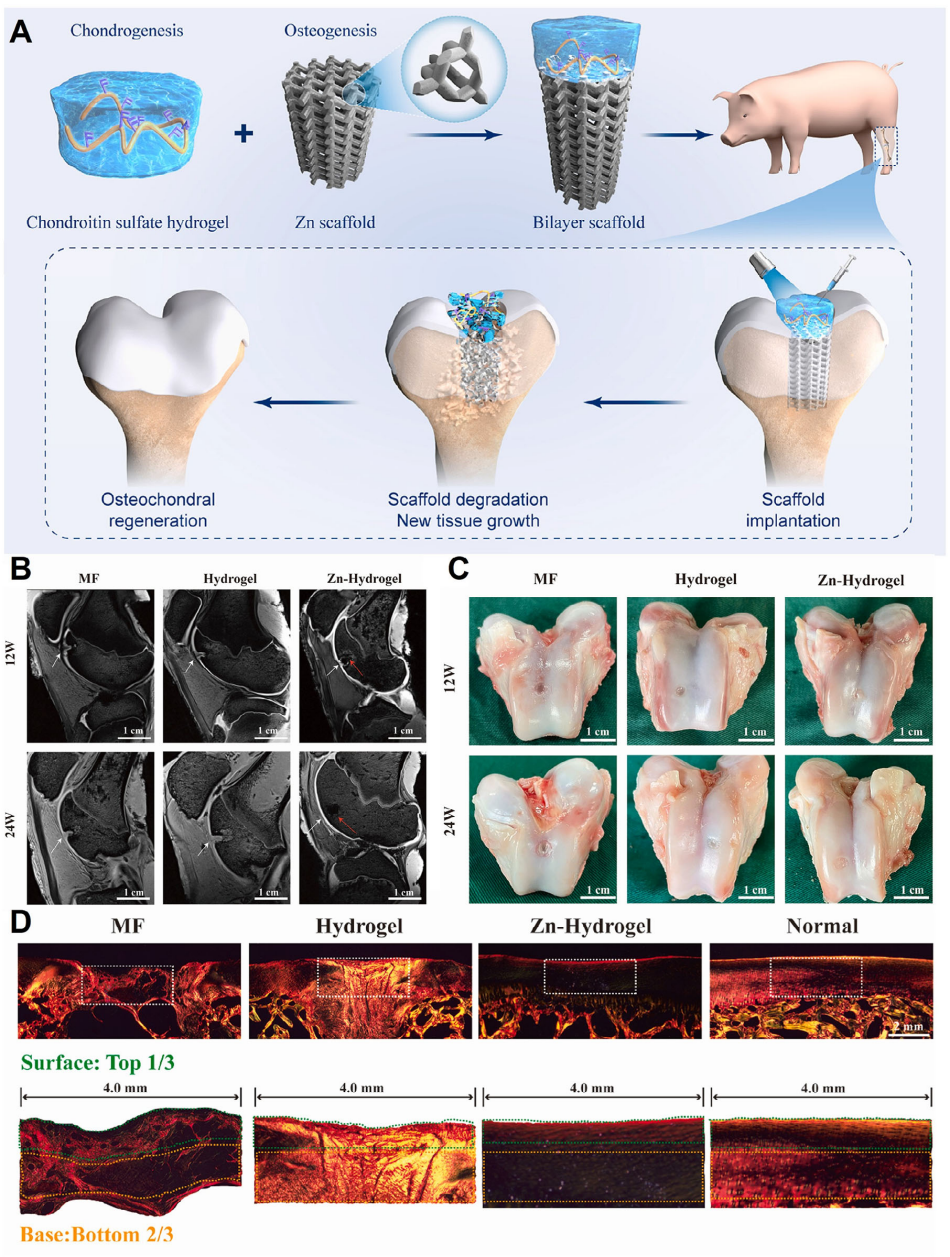
Bilayer scaffold composed of chondroitin sulfate (CS) hydrogel and porous zinc for osteochondral defect repair. (A) Schematic illustration of the bilayer scaffold designed with CS hydrogel and porous zinc for the repair of osteochondral defects. (B) Magnetic resonance imaging (MRI) of knee joint repair at different time points (white arrows: repair sites; red arrows: zinc scaffold). (C) Macroscopic observation of cartilage defect repair at weeks 12 and 24. (D) Picrosirius Red staining of regenerated tissue and normal cartilage to evaluate collagen organization. Reproduced under the terms of the CC BY‐NC‐ND 4.0 license [195]. Copyright 2024, The Author(s).

Yang et al. [195] developed a bilayer scaffold composed of photocurable CS hydrogel and porous pure zinc scaffold for osteochondral repair (Figure 9A). The cartilage layer adopted photocurable CS hydrogel (compressive strength 82 kPa), and the bone layer adopted a porous zinc scaffold (yield strength 11 MPa, stiffness 0.8 GPa), mimicking the osteochondral gradient structure. In a Bama miniature pig trochlear groove defect model, this scaffold promoted synchronous bone and cartilage regeneration. Magnetic resonance imaging showed effective defect filling at 12 and 24 weeks post‐implantation, with gradual zinc scaffold degradation (Figure 9B). Macroscopic observation revealed smooth repaired cartilage surfaces, forming hyaline‐like cartilage with good integration with surrounding tissues (Figure 9C). Picrosirius red staining showed collagen fiber arrangement in regenerated tissue similar to normal cartilage (Figure 9D).

CS can be functionalized through chemical modification. Methacrylated CS (CS‐MA) can be photocrosslinked to form hydrogels, with mechanical properties and degradation rate controllable through crosslinking density, and has been used for encapsulating stem cells and chondrocytes. CS nanoparticles can encapsulate anti‐inflammatory drugs for intra‐articular targeted delivery, and CS‐modified liposomes target chondrocytes through CD44 receptor‐mediated endocytosis [195].

Oral CS as a dietary supplement for osteoarthritis treatment has a history of several decades, but its efficacy remains controversial, which may be related to CS source, purity, molecular weight, and sulfation patterns [242]. In tissue engineering applications, CS faces challenges including source dependency, purification difficulty, immunogenicity risk, and high cost.

##### 5.1.3.3 Hyaluronic Acid

Hyaluronic acid (HA), also known as hyaluronan or sodium hyaluronate, is a non‐sulfated glycosaminoglycan, a linear macromolecular polysaccharide formed by alternating linkages of D‐glucuronic acid and N‐acetylglucosamine through β‐1,3 and β‐1,4 glycosidic bonds [243]. HA is an important component of synovial fluid and cartilage ECM, playing key roles in joint lubrication, cartilage protection, and cell signal transduction. Hyaluronic acid was initially isolated from bovine vitreous humor, and currently animal‐derived HA is mainly extracted from tissues such as rooster comb, bovine vitreous humor, and umbilical cord, with molecular weight typically between 500–2000 kDa. Although microbial fermentation has become the mainstream production method, animal‐derived HA still holds a position in high‐end medical applications due to its natural structure and biological properties [199].

The biological functions of hyaluronic acid are highly dependent on its molecular weight. High molecular weight HA (>1000 kDa) possesses anti‐inflammatory, immunosuppressive, and tissue‐protective effects, capable of inhibiting macrophage activation, reducing pro‐inflammatory factor release, and promoting chondrocyte survival and matrix synthesis through CD44 receptors, providing lubrication and cushioning functions in synovial fluid. Low molecular weight HA (<200 kDa) exhibits pro‐inflammatory effects, activating TLR2/TLR4 signaling pathways and inducing pro‐inflammatory factor production. During cartilage degradation, hyaluronidase and free radicals degrade HA, reducing its molecular weight and leading to aggravated joint inflammation. Medium molecular weight HA (200–1000 kDa) demonstrates balanced biological effects in cartilage repair [244].

Hyaluronic acid has diverse application forms in cartilage repair. High molecular weight HA as a viscosupplementation for osteoarthritis treatment has a history of over 30 years, capable of improving joint lubrication, reducing pain, and improving joint function. Crosslinked HA (such as Hylan G‐F 20) has increased molecular weight and viscoelasticity through chemical crosslinking, prolonging intra‐articular retention time. Methacrylated hyaluronic acid (HA‐MA) forms hydrogels through photocrosslinking with tunable mechanical properties and degradation rate, widely used in cartilage tissue engineering [245]. HA is commonly combined with other materials to improve mechanical properties, such as HA‐collagen composite hydrogels, HA‐chitosan composite scaffolds, and HA‐nano‐hydroxyapatite composite materials. Multiple HA‐based products have been approved for cartilage repair, such as Hyalograft C and Hyalofast, with clinical studies showing these products can promote cartilage‐like tissue formation and improve patient symptoms [244].

Hyaluronic acid regulates chondrocyte behavior through interactions with cell surface receptors. CD44 is the primary HA receptor, mediating HA regulation of chondrocyte proliferation, migration, and matrix synthesis, activating PI3K/Akt, MAPK, and Rho GTPase pathways to promote chondrocyte phenotype maintenance. RHAMM is another important HA receptor, participating in cell migration and proliferation regulation [199]. HA can also influence immune responses through TLR4 receptors, with low molecular weight HA fragments acting as damage‐associated molecular patterns (DAMPs) to activate innate immune responses [89].

The application of hyaluronic acid in cartilage repair still faces challenges, including rapid degradation, insufficient mechanical properties, molecular weight control, and cost issues. Future research directions include developing chemically modified HA (such as sulfated HA, phosphorylated HA) to enhance bioactivity, designing HA‐growth factor conjugates for targeted delivery, and developing dynamic HA hydrogels with self‐healing capabilities.

#### 5.1.4 Insect‐Derived Proteins

##### 5.1.4.1 Silk Protein

Silk is a natural protein fiber secreted by the silk glands of the domesticated silkworm (Bombyx mori), with an application history of over 5000 years [196]. As a renewable biomaterial, silk has received widespread attention in the tissue engineering field due to its excellent biocompatibility, tunable degradability, good mechanical properties, and easy processability. Silk is mainly composed of fibroin and sericin, which differ significantly in structure, function, and biological properties, together conferring unique material properties to silk [246].

Silk fibers consist of an inner fibroin core and an outer sericin coating layer. Fibroin accounts for 70%–80% of silk dry weight, composed of heavy chain (approximately 350 kDa), light chain (approximately 25 kDa), and P25 glycoprotein (approximately 30 kDa) through disulfide bonds in a 6:6:1 molar ratio, forming a complex. Its heavy chain is rich in glycine (Gly), alanine (Ala), and serine (Ser), with repetitive sequences (such as GAGAGS) forming highly ordered β‐sheet crystal structures through hydrogen bonds, conferring excellent tensile strength (500–740 MPa) and toughness to silk. Sericin accounts for 20%–30% of dry weight, is a water‐soluble protein rich in serine (approximately 30%), aspartic acid, and glycine, distributed on the fibroin fiber surface, serving adhesive and protective functions [196].

Domesticated silkworm silk differs from wild silkworm silk (such as Antheraea pernyi) in amino acid composition. Wild silkworm silk contains more charged amino acids and RGD cell adhesion sequences, showing superior performance in promoting cell attachment and proliferation, but its mechanical properties and processing stability are inferior to domesticated silkworm silk. Domesticated silkworm silk has become the most commonly used silk source in tissue engineering research due to its standardized production, strong controllability, and excellent mechanical properties [247].

Silk possesses good biocompatibility, with mild inflammatory responses after in vivo implantation, capable of supporting chondrocyte, osteoblast, and mesenchymal stem cell adhesion, proliferation, and differentiation. The degradation rate of silk fibroin can be precisely controlled by regulating β‐sheet content and crosslinking degree, with degradation periods ranging from weeks to years, suitable for different tissue regeneration requirements. Silk is mainly degraded by proteases (such as chymotrypsin and collagenase), with degradation products being amino acids and small peptides that can be absorbed and metabolized by the body without significant toxicity. The immunogenicity of silk fibroin primarily originates from sericin residues. Undegummed silk may trigger allergic reactions; therefore, biomedical applications typically require removal of sericin through alkaline treatment (such as boiling in sodium carbonate solution) or enzymatic treatment [248]. Degummed silk fibroin exhibits significantly reduced immunogenicity and has been FDA‐approved for surgical sutures. Recent studies have shown that moderate retention or separate application of sericin can exert biological activities such as promoting cell proliferation, antioxidant, and anti‐inflammatory effects.

The multi‐scale processability of silk enables the fabrication of various scaffold morphologies, including fibers, films, sponges, hydrogels, and composite scaffolds [246]. Silk fibroin fibers can be constructed into scaffolds with high porosity and directionally aligned structures through weaving or electrospinning techniques, mimicking the anisotropy of natural cartilage and bone tissues; silk fibroin sponges are prepared through freeze–drying or salt‐leaching methods, with tunable pore size and porosity; silk fibroin hydrogels achieve in situ gelation through ultrasound, vortexing, or enzyme induction, possessing injectability and minimally invasive treatment potential [249]. In osteochondral tissue engineering, the advantages of silk lie in its excellent mechanical properties and tunable degradability, capable of providing long‐term mechanical support for nascent tissue. By regulating β‐sheet content and crosslinking degree, the different mechanical requirements of cartilage and bone layers can be matched [196]. Furthermore, silk can be easily combined with materials such as collagen, hyaluronic acid, hydroxyapatite, and polycaprolactone to construct biphasic or multiphasic scaffolds with layered structures and gradient properties, mimicking the complex microenvironment of the natural osteochondral interface. As a natural, renewable biomaterial with excellent properties, silk has broad application prospects in osteochondral tissue engineering.

##### 5.1.4.2 Silk Fibroin

Silk fibroin is the major structural component of silk, accounting for 70%–80% of silk dry weight. Due to its exceptional mechanical strength, tunable degradability, and good biocompatibility, it has become one of the most promising natural polymer materials in osteochondral tissue engineering [247]. The unique molecular structure and diverse processing forms of silk fibroin enable it to meet the multiple requirements for mechanical support, cellular microenvironment, and tissue integration in osteochondral repair.

Silk fibroin is composed of a complex consisting of a heavy chain (approximately 350 kDa), light chain (approximately 25 kDa), and P25 glycoprotein, with the heavy chain being the key component determining material properties. The heavy chain is rich in glycine (Gly), alanine (Ala), and serine (Ser), with typical repeat units such as GAGAGS, GAGAGY, and GAGAGVGY self‐assembling through intermolecular hydrogen bonds to form β‐sheet crystal structures, conferring excellent mechanical strength and stability to silk fibroin [196]. The higher the β‐sheet content, the greater the tensile strength and Young's modulus of the material, and the slower the degradation rate. By regulating processing conditions (such as methanol treatment, water vapor annealing, ultrasound induction), β‐sheet content can be precisely controlled, enabling customized design of mechanical properties and degradation rates. These tunable structural features directly translate into favorable biological performance in vivo.

**FIGURE 10:**
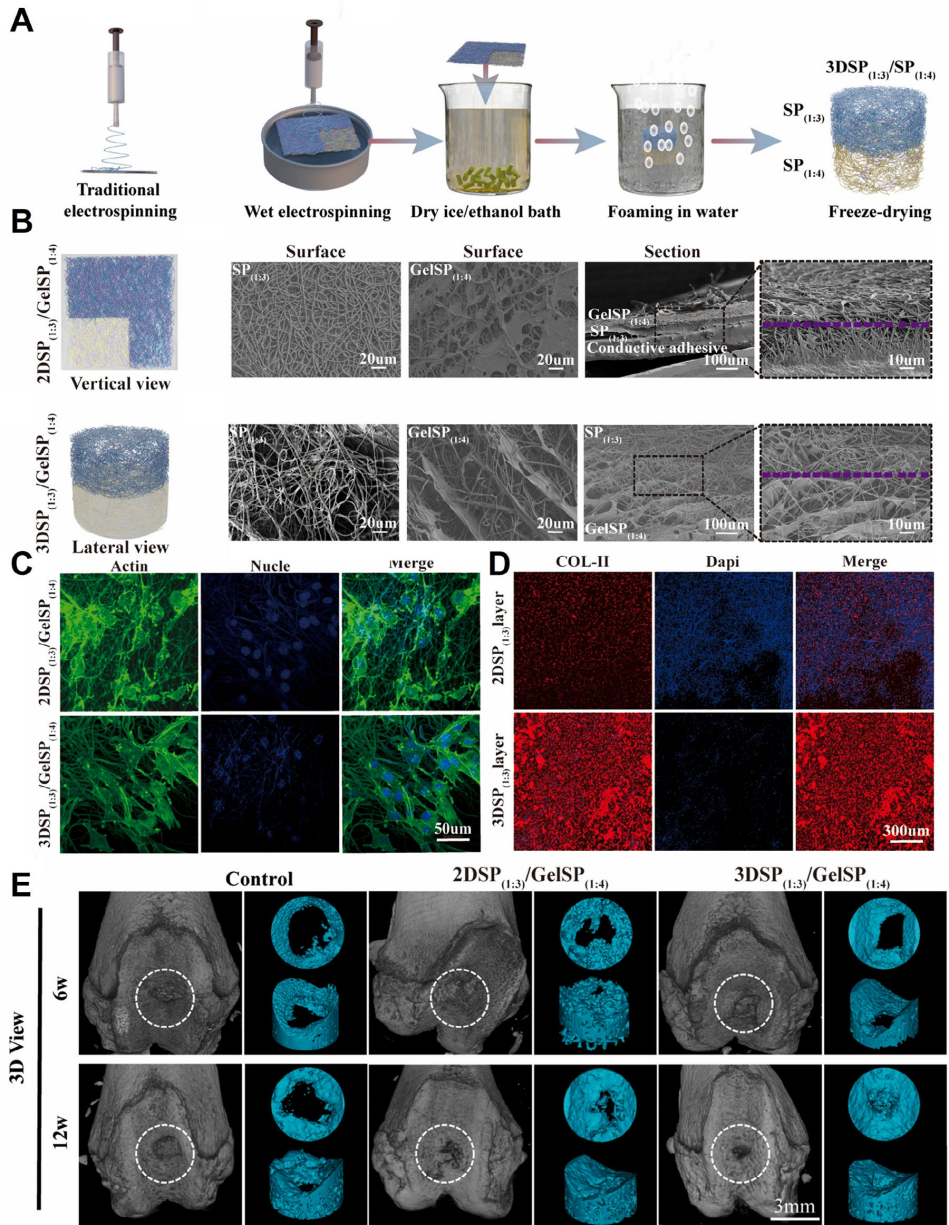
Preparation, characterization, and evaluation of 2D/3D SP (1:3)/GelSP (1:4) scaffolds for osteochondral defect repair. (A) Schematic illustration of the preparation process for 2DSP (1:3)/SP (1:4) and 3DSP (1:3)/SP (1:4). (B) Preparation process of 2DSP (1:3)/GelSP (1:4) and 3DSP (1:3)/GelSP (1:4) scaffolds (top and side views), along with surface and cross‐sectional SEM images showing scaffold morphology. (C) Cytoskeleton staining of MC3T3‐E1 cells cultured on different scaffolds for 3 days, demonstrating cellular attachment and spreading. (D) Immunofluorescence staining of COL‐II in ATDC5 cells cultured on 2D/3D SP (1:3)/GelSP (1:4) scaffolds for 14 days, highlighting chondrogenic differentiation. (E) Macroscopic observation and radiological evaluation of rat osteochondral defects, including micro‐CT 3D reconstruction of the repaired sites. Reproduced under the terms of the CC BY‐NC‐ND 4.0 license [196]. Copyright 2025, The Author(s).

In recent years, significant progress has been made in the application research of silk fibroin in osteochondral tissue engineering. Studies have shown that silk fibroin scaffolds can support chondrogenic and osteogenic differentiation of mesenchymal stem cells, promoting the expression of cartilage‐specific genes (such as COL2A1, ACAN, SOX9) and bone‐specific genes (such as RUNX2, OCN, OPN). Zhang et al. [196] developed an innovative silk fibroin/PCL bilayer integrated scaffold that successfully addressed the interface delamination problem of traditional biphasic scaffolds. This study employed layer‐by‐layer wet electrospinning technology to prepare bilayer two‐dimensional membranes, subsequently converting the 2D membranes into 3D scaffolds with continuous interfaces and heterogeneous bilayer structures through CO_2_ gas foaming technology, with gelatin coated in situ on the osteogenic layer to enhance mechanical properties (Figure 10A,B). In vitro studies demonstrated that 3D scaffolds could better maintain cell phenotype compared to traditional 2D electrospun membranes. MC3T3‐E1 osteoblasts cultured on 3D scaffolds for 3 days showed good cytoskeletal alignment (Figure 10C), and ATDC5 chondrocytes cultured on 3D silk fibroin/gelatin scaffolds for 14 days exhibited significantly enhanced type II collagen (COL‐II) expression (Figure 10D). In a rat osteochondral defect model, 3D scaffolds implanted for 12 weeks showed excellent repair effects: good cartilage layer regeneration, significantly reduced defect area, increased subchondral bone volume, Micro‐CT three‐dimensional reconstruction showed adequate bone tissue regeneration, and the scaffold‐host tissue interface was well integrated without delamination (Figure 10E).

Silk fibroin demonstrates significant advantages in osteochondral tissue engineering, including exceptional mechanical strength (tensile strength 500–740 MPa), degradation rate tunable through β‐sheet content regulation, good biocompatibility and low immunogenicity, diverse processing forms (fibers, sponges, hydrogels), and ease of compositing with inorganic materials or polymers to construct synergistic properties [199]. However, silk fibroin also has some limitations: complex extraction and purification processes lead to batch‐to‐batch performance variations, natural silk fibroin lacks cell recognition sites requiring modification to improve adhesion, matching of degradation rate with nascent tissue formation rate still needs optimization, the porous structure and mechanical properties of layered scaffolds are difficult to simultaneously meet the differential requirements of cartilage and bone layers, and interface integration and long‐term stability remain technical challenges [242]. Furthermore, clinical translation research on silk fibroin‐based scaffolds is relatively limited, and long‐term safety and efficacy data still need further accumulation. Nevertheless, through material design optimization, functionalization modification, and composite strategies, silk fibroin scaffolds still have broad application prospects in osteochondral repair, providing an important material foundation for clinical translation.

##### 5.1.4.3 Sericin

Sericin, the outer coating protein comprising 20%–30% of silk dry weight, was traditionally discarded during silk processing. Recent studies have revealed its unique bioactivities, including promoting cell proliferation, antioxidant, anti‐inflammatory, and moisturizing functions, offering new opportunities for osteochondral tissue engineering while improving silk resource utilization.

Sericin is a water‐soluble globular protein with molecular weights ranging from 20 to 400 kDa, predominantly composed of serine (30%–35%), aspartic acid, glycine, and threonine [250]. Rich in hydroxyl and carboxyl groups, sericin exhibits excellent hydrophilicity and can bind to cell surface receptors through hydrogen bonding and electrostatic interactions. Its variable composition, depending on silkworm species and extraction methods provides flexibility for functionalized applications.

Sericin demonstrates multiple beneficial biological activities. It significantly promotes proliferation of chondrocytes, osteoblasts, and mesenchymal stem cells by binding integrin receptors, activating MAPK and PI3K/Akt signaling pathways, and enhancing ECM synthesis. Its hydroxyl‐rich amino acids confer potent antioxidant activity, scavenging free radicals and enhancing antioxidant enzyme activity to protect chondrocytes from oxidative stress [251]. Additionally, sericin exhibits anti‐inflammatory effects by suppressing pro‐inflammatory cytokines through inhibition of NF‐κB and MAPK pathways, thereby modulating the inflammatory microenvironment during osteochondral repair.

Sericin can be processed into diverse forms, including solutions, hydrogels, films, and sponges, through various methods. It readily composites with other materials to create synergistic scaffolds: combining with silk fibroin integrates mechanical strength and bioactivity; with collagen or hyaluronic acid enhances chondrogenic capacity; and with hydroxyapatite improves osteoconductivity [196, 243]. Studies demonstrate that sericin promotes chondrogenic differentiation, enhances cartilage‐specific gene expression, and improves cell adhesion on scaffold surfaces. However, challenges remain, including weak mechanical properties requiring composite strategies, rapid degradation necessitating crosslinking modifications, and the need for standardized extraction methods to ensure batch consistency. Long‐term in vivo safety and immune responses also require further evaluation. Despite these limitations, sericin holds significant promise in osteochondral tissue engineering as a bioactive component to enhance scaffold performance and tissue regeneration.

### 5.2 Plant/Algae‐Derived Materials

#### 5.2.1 Algal Polysaccharides

Plant and algae‐derived biomaterials have attracted widespread attention in osteochondral tissue engineering due to their abundant natural resources, good biocompatibility, renewability, and environmental friendliness. Compared with animal‐derived materials, plant/algae polysaccharides have lower immunogenicity and disease transmission risk, with stable sources, lower costs, and ease of large‐scale production. Algal polysaccharides (such as alginate, agarose, carrageenan) are typical representatives of this class of materials, and their unique gelation properties, tunable mechanical properties, and good cytocompatibility demonstrate significant application value in cartilage repair, three‐dimensional cell culture, and injectable hydrogels.

##### 5.2.1.1 Alginate (Brown Algae)

**FIGURE 11:**
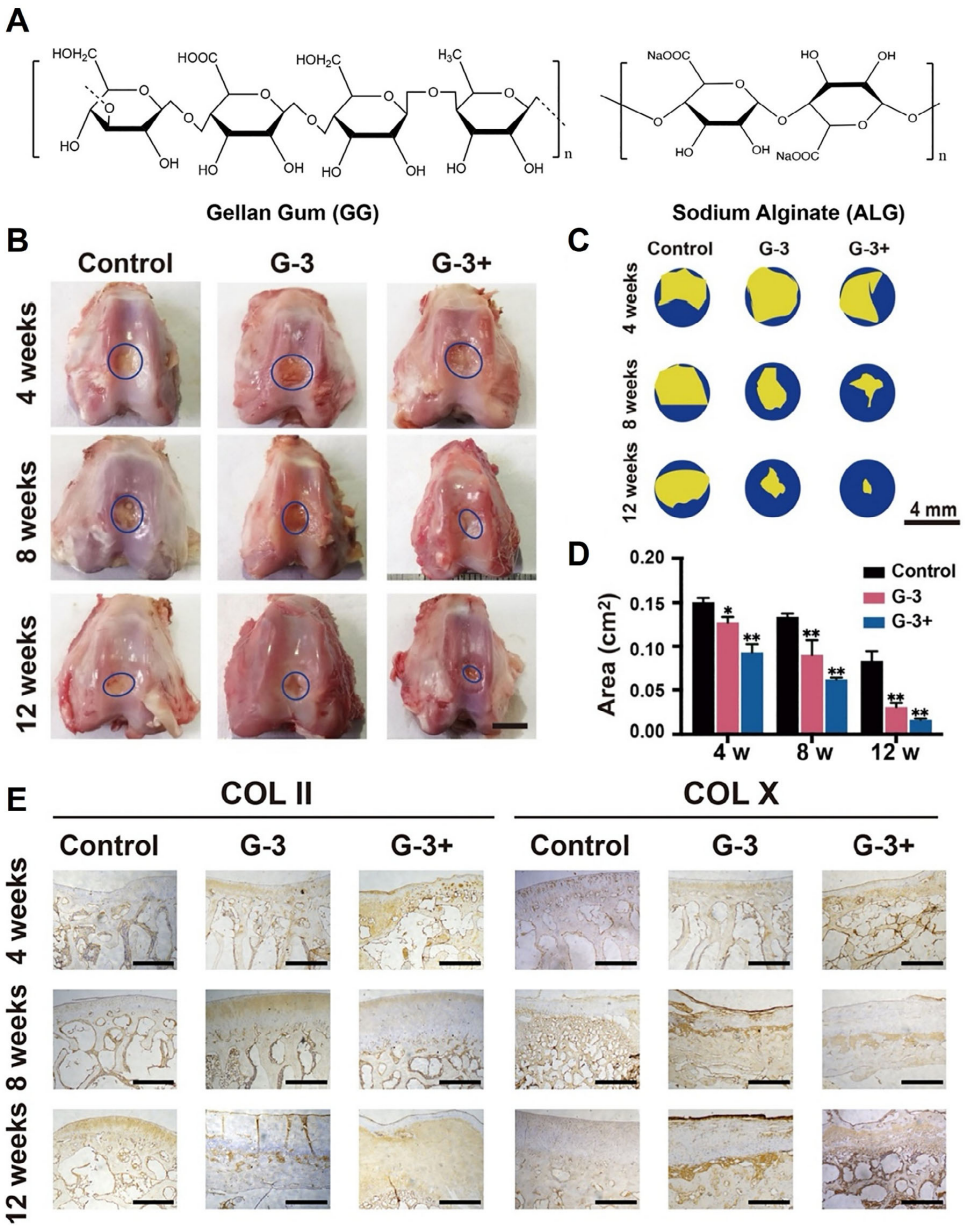
Chemical structures, macroscopic observations, and immunohistochemical analyses for evaluating polysaccharide‐based scaffolds in osteochondral defect repair. (A) Chemical structures of two polysaccharides: gellan gum and sodium alginate. (B) Photographs of defect areas (blue circles) in different groups at 4, 8, and 12 weeks post‐surgery (scale bar = 1 cm). (C) Schematic illustration of defect regeneration in vivo for different groups. The yellow region represents the healing area, while the blue region indicates the initial defect area at 4, 8, and 12 weeks post‐surgery (scale bar = 4 mm). (D) Semi‐quantitative evaluation of the repair area using Image J, a scientific image analysis software. (E) Schematic representation of specific immunohistochemical analyses for different regions. Type II collagen (COL II) is used to evaluate cartilage, and type X collagen (COL X) is used to assess the cartilage‐subchondral bone interface. Specific immunohistochemical analyses of critical defect areas in rabbits were performed to assess pathological and biochemical changes at 4, 8, and 12 weeks post‐surgery (scale bar = 200 µm, magnification ×40). * indicates a significant difference compared to the control group at the same time point (*p < 0.05, **p < 0.01). Reproduced with permission [197]. Copyright 2021, Elsevier.

Alginate is a linear anionic polysaccharide extracted from brown algae (such as kelp, giant kelp), composed of β‐D‐mannuronic acid (M units) and α‐L‐guluronic acid (G units) linked by 1,4‐glycosidic bonds, forming three different block structures: MM, GG, and MG (Figure 11A). The gelation properties of alginate primarily depend on the ionic crosslinking between G units and divalent cations (such as Ca^2^^+^, Ba^2^^+^, Sr^2^^+^), forming physical hydrogels with an “egg‐box” structure [197]. The higher the GG block content, the better the mechanical strength and stability of the gel. Alginate hydrogels possess good biocompatibility, mild gelation conditions (room temperature, physiological pH), and injectability, making them one of the most commonly used natural hydrogel materials in osteochondral tissue engineering [252].

The mechanical properties of alginate hydrogels can be optimized by regulating polymer concentration, molecular weight, G/M ratio, and crosslinker type. Typical alginate hydrogels (2%–4% w/v) have a compressive modulus of 10–100 kPa, which is lower compared to native cartilage tissue (0.5–2 MPa), but can be significantly enhanced through strategies such as covalent crosslinking, double‐network structures, or nanocomposite reinforcement. The degradation of alginate hydrogels occurs primarily through ion exchange and oxidative degradation, with the degradation rate closely related to crosslinking density, molecular weight, and the in vivo ionic environment. Through partial oxidation or grafting of degradable groups, precise control of the degradation rate can be achieved.

Alginate hydrogels lack cell adhesion sites and are unfavorable for cell adhesion and proliferation in their native state. To improve cytocompatibility, functionalization modifications are commonly performed through strategies such as RGD peptide grafting, gelatin blending, or growth factor loading. The injectability of alginate hydrogels makes them particularly suitable for repairing irregularly shaped cartilage defects. In recent years, alginate‐based bilayer hydrogels have demonstrated significant advantages in osteochondral repair. For example, Xing et al. [197] developed a calcium‐rich bilayer hydrogel composed of gellan gum/alginate. The cartilage layer of this hydrogel consists of gellan gum and sodium alginate, while the subchondral bone layer is composed of gellan gum and hydroxyapatite, mimicking the layered structure and compositional gradient of osteochondral tissue. In a rabbit knee critical‐size defect model (diameter 4.0 mm, depth 8.0 mm), this calcium‐rich hydrogel served as a calcium reservoir, promoting neovascularization at 4 weeks post‐surgery and significantly repairing the defect area at 8 weeks (Figure 11B–D). Histological and immunohistochemical analyses revealed that hyaline cartilage‐like tissue formed in the repair region, with type II collagen (COL II) highly expressed in the cartilage layer and type X collagen (COL X) expressed at the cartilage‐subchondral bone interface, indicating that the hydrogel successfully induced stratified tissue regeneration (Figure 11E). Additionally, alginate microspheres and microfibers are widely used for cell encapsulation, drug sustained release, and 3D bioprinting, providing diverse application forms for osteochondral tissue engineering.

##### 5.2.1.2 Agarose (Red Algae)

Agarose is a neutral polysaccharide extracted from red algae (such as Gelidium, Gracilaria), the main component of agar, formed by alternating linkages of D‐galactose and 3,6‐anhydro‐L‐galactose through α‐1,3 and β‐1,4 glycosidic bonds to form a linear polymer. Agarose possesses unique thermoreversible gelation properties: it dissolves to form a solution at high temperatures (>65°C) and spontaneously forms a three‐dimensional network hydrogel through intermolecular hydrogen bonding when cooled to 35°C–40°C [253]. This mild gelation mechanism requires no chemical crosslinkers, avoiding cytotoxicity issues, making agarose an ideal matrix material for three‐dimensional cell culture and tissue engineering. Agarose hydrogels have high porosity (typically >90%), with pore size controllable through concentration adjustment (0.5%–4% w/v corresponding to pore sizes of 100–500 nm), facilitating nutrient diffusion and cell migration.

The mechanical properties of agarose hydrogels are closely related to concentration and molecular weight. Low‐concentration agarose (0.5%–1% w/v) forms softer hydrogels (compressive modulus 1–10 kPa), suitable for mimicking the soft matrix environment of cartilage tissue; high‐concentration agarose (2%–4% w/v) can achieve compressive moduli of 50–200 kPa, approaching the mechanical range of native cartilage. The elastic modulus and shear modulus of agarose hydrogels can be precisely controlled by regulating gelation temperature, cooling rate, and polymer concentration, providing an excellent experimental platform for studying cellular mechanical responses [254]. The degradation rate of agarose in vivo is relatively slow, occurring primarily through enzymatic degradation (agarase) and hydrolysis, with degradation periods ranging from several weeks to months, making it suitable for long‐term tissue regeneration applications.

The primary application of agarose hydrogels in osteochondral tissue engineering is as a three‐dimensional cell culture matrix. Its inert surface and tunable porous structure can maintain the rounded morphology and chondrogenic phenotype of chondrocytes, inhibiting cell dedifferentiation. Studies have shown that chondrocytes cultured in agarose hydrogels maintain high levels of type II collagen and aggrecan expression, significantly superior to two‐dimensional culture conditions. Agarose hydrogels are also commonly used to construct in vitro models of cartilage tissue for studying cartilage development, degeneration, and drug screening. However, agarose has weak interactions with cells and lacks cell adhesion sites, limiting its effectiveness in in vivo implantation applications. To improve bioactivity, it is commonly combined with materials containing cell adhesion sites such as collagen, gelatin, and hyaluronic acid, or chemically modified to graft bioactive molecules such as RGD peptides and growth factors [232]. Additionally, the thermoreversibility of agarose makes it suitable for 3D bioprinting applications, where it can serve as a support material or sacrificial template to assist in constructing complex tissue structures.

##### 5.2.1.3 Carrageenan (Red Algae)

Carrageenan is a sulfated linear polysaccharide from red algae (Eucheuma, Hypnea), composed of D‐galactose and 3,6‐anhydro‐D‐galactose linked via α‐1,3 and β‐1,4 glycosidic bonds. Based on sulfate content, it is classified into three types: κ‐carrageenan (1 sulfate group) forms hard, brittle gels with K^+^/Ca^2^^+^; ι‐carrageenan (2 sulfate groups) produces soft, elastic gels; λ‐carrageenan (3 sulfate groups) does not gel but provides thickening properties [255]. Gelation involves coil‐to‐helix transition, helix aggregation, and ionic crosslinking. These hydrogels exhibit good biocompatibility, reversible thermogelling, and tunable mechanics, suitable for injectable scaffolds and cell encapsulation in osteochondral tissue engineering.

Mechanical properties depend on type and ionic environment. κ‐Carrageenan gels at 40°C–50°C with K^+^ (compressive modulus: 10–50 kPa); ι‐carrageenan gels at 30°C–40°C with Ca^2^^+^ (5–30 kPa). Properties are controlled by concentration (1%–3% w/v), ion type, and concentration [256]. Degradation occurs via sulfatases and glycosidases, with rates influenced by sulfation degree and molecular weight; highly sulfated forms degrade faster.

In osteochondral tissue engineering, carrageenan hydrogels are used for cartilage repair and cell delivery. Their near‐physiological gelation temperature and injectability enable minimally invasive treatment. κ‐Carrageenan supports chondrocyte function and maintains chondrogenic phenotype. Sulfate groups bind growth factors (TGF‐β, BMP‐2) for sustained release, promoting regeneration [256]. However, low mechanical strength limits standalone use in load‐bearing sites. Composites with nano‐hydroxyapatite, collagen, or chitosan enhance properties. Sulfate groups may cause mild inflammation, requiring purification. Recent advances include double‐network and self‐healing hydrogels with covalent or dynamic bonds (Schiff base, boronate ester), significantly improving mechanical stability for osteochondral applications.

#### 5.2.2 Plant Polysaccharides

Plant polysaccharides are natural polymers extracted from terrestrial plants, including cellulose derivatives, starch, pectin, locust bean gum, and guar gum. They offer wide availability, low cost, biocompatibility, biodegradability, and easy modification, showing excellent potential in osteochondral tissue engineering. Unlike animal‐derived polysaccharides, they lack immunogenicity and pathogen transmission risk, meeting clinical safety requirements. Chemical modifications (methylation, carboxymethylation, methacrylation) introduce photocrosslinking, thermosensitivity, and pH responsiveness for smart hydrogels. However, low mechanical strength and uncontrolled degradation limit their use in load‐bearing sites. Nanocomposite reinforcement, double‐network design, and covalent crosslinking can enhance properties. This section focuses on cellulose derivatives and locust bean gum in osteochondral tissue engineering.

##### 5.2.2.1 Cellulose and its Derivatives

Cellulose is a linear polysaccharide of D‐glucose linked via β‐1,4‐glycosidic bonds, abundant in plant cell walls. Its highly ordered crystalline structure provides excellent mechanical strength and stability. However, natural cellulose is water‐insoluble and lacks cell adhesion sites, limiting its direct tissue engineering use. Chemical modification produces derivatives like carboxymethyl cellulose (CMC), hydroxyethyl cellulose (HEC), and methyl cellulose (MC) with improved water solubility, biocompatibility, and tunable gelation, widely used as hydrogel matrices, drug carriers, and 3D printing inks in osteochondral tissue engineering [257].

CMC, an anionic derivative with carboxymethyl groups, forms hydrogels via ionic (Ca^2^^+^, Zn^2^^+^) or covalent (genipin, EDC/NHS) crosslinking. CMC hydrogels support chondrocyte function and phenotype, and composites with HA, bioactive glass, or growth factors enhance osteochondral repair. CMC/gelatin/nano‐HA hydrogels facilitate cartilage and subchondral bone regeneration, while CMC's shear‐thinning property enables 3D bioprinting of complex constructs. MC and HPMC, nonionic derivatives, exhibit thermoreversible gelation above 40°C–50°C, suitable for injectable systems. MC hydrogels encapsulate MSCs and chondrocytes, supporting viability and chondrogenic differentiation. MC/chitosan/β‐GP composites show improved mechanical strength and sustained growth factor release, promoting cartilage matrix formation. HEC demonstrates good biocompatibility and low immunogenicity, forming hydrogels via chemical crosslinking or polymer blending. Its high water retention is ideal for drug delivery and cell encapsulation. HEC hydrogels loaded with kartogenin enhance cartilage regeneration in animal models [258].

Cellulose derivatives have relatively low mechanical strength for load‐bearing applications. Strategies include double‐network hydrogels, nanocomposite reinforcement, and interpenetrating polymer networks. CMC/polyacrylamide double‐networks achieve cartilage‐comparable compressive strength. Nanocellulose fiber incorporation significantly enhances mechanical properties, offering promise for osteochondral tissue engineering.

##### 5.2.2.2 Locust Bean Gum

Locust bean gum (LBG) is a non‐ionic galactomannan from carob tree (Ceratonia siliqua) seeds, consisting of β‐1,4‐linked D‐mannose backbone with α‐1,6‐linked D‐galactose side chains. LBG exhibits good biocompatibility, biodegradability, low toxicity, and FDA GRAS status. As a non‐ionic hydrocolloid, LBG solutions show stability across pH, salt, and temperature variations [259]. Abundant hydroxyl groups enable chemical modification for photocrosslinking, ionic crosslinking, or covalent crosslinking capabilities.

**FIGURE 12:**
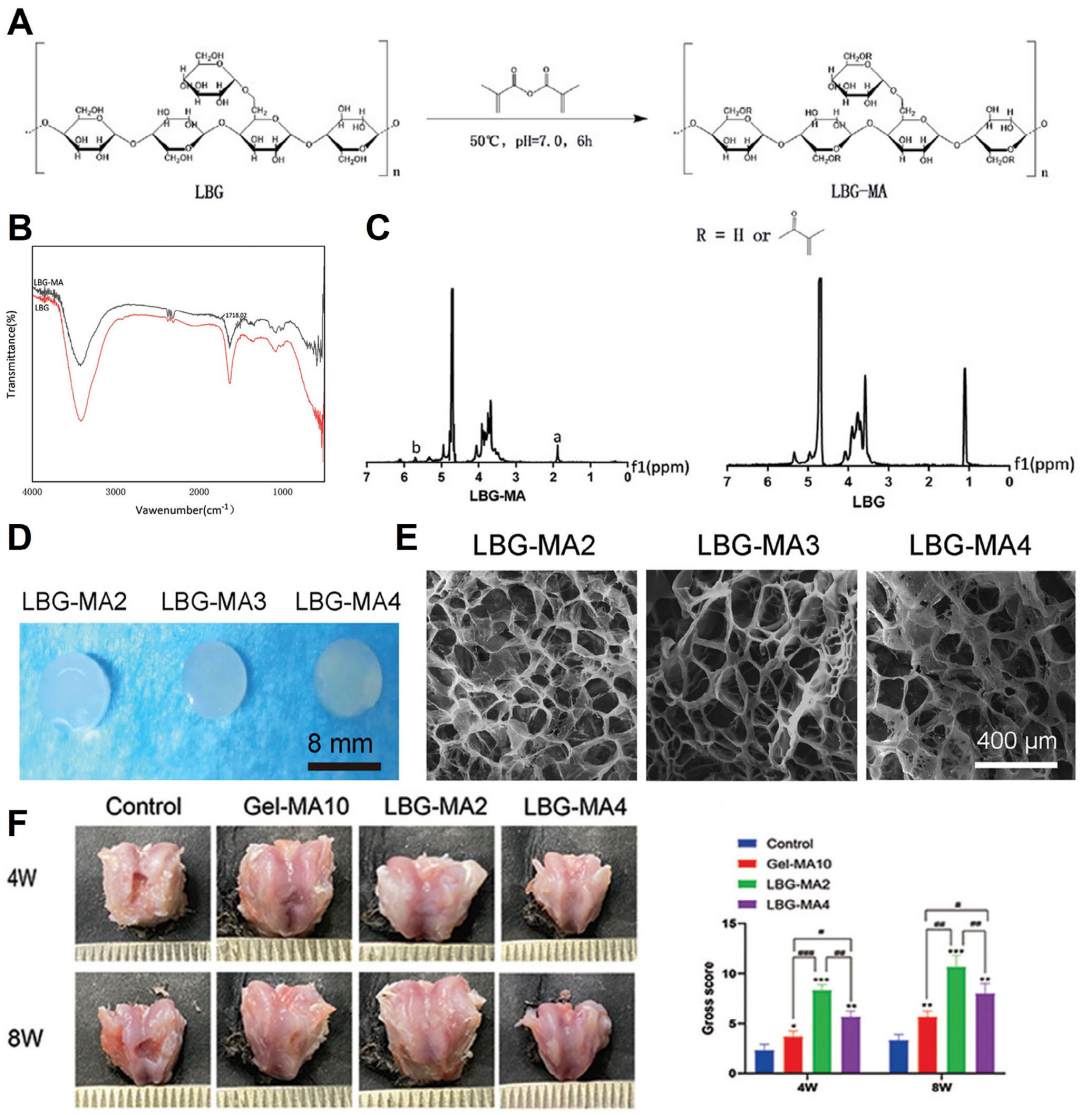
Synthesis, characterization, and evaluation of LBG‐MA hydrogels for engineered cartilage. (A) Synthesis and characterization of locust bean gum methacryloyl (LBG‐MA) polymer. The schematic shows the synthetic route for methacryloyl (MA) modification of locust bean gum (LBG). (B) FTIR spectra of LBG and LBG‐MA polymers, indicating the successful introduction of methacryloyl groups. (C) ^1^H NMR spectra of LBG and LBG‐MA polymers, confirming the chemical structure and degree of modification. (D) Gelation process of LBG‐MA hydrogel under 365 nm UV light irradiation. (E) SEM images of LBG‐MA hydrogel, showing its porous structure (scale bar = 400 µm). (F) Macroscopic images and macroscopic scores of engineered cartilage generated using LBG‐MA hydrogels. Data are presented as mean ± standard deviation (*n* = 3); *, # indicate *p* < 0.05; **, ## indicate *p* < 0.01; ***, ### indicate *p* < 0.001. Reproduced under the terms of the CC BY‐NC‐ND 4.0 license [198]. Copyright 2023, The Author(s).

Natural LBG hydrogels have weak mechanical strength, limiting tissue engineering applications [198]. Strategies to enhance properties include compositing with other polysaccharides, ionic crosslinking, double‐network construction, and chemical modification. Methacrylated LBG (LBG‐MA) has become a research focus. LBG‐MA forms covalently crosslinked networks via UV (365 nm)‐initiated polymerization, significantly improving mechanical strength and stability (Figure 12A–D). Crosslinking density is controlled by adjusting methacrylation degree and polymer concentration, optimizing mechanical properties and degradation rates. SEM reveals highly interconnected porous structures (50–200 µm), facilitating cell migration, nutrient diffusion, and tissue integration (Figure 12E).

LBG‐MA hydrogels demonstrate significant chondrogenic induction in osteochondral tissue engineering. In vitro, they promote BMSC differentiation into chondrocytes, with high GAG accumulation and upregulated chondrogenic genes (type II collagen, aggrecan, SOX9). Matrix stiffness regulates BMSC chondrogenic differentiation; optimized LBG‐MA concentration matches native cartilage mechanics. Injectable LBG‐MA enables minimally invasive delivery to irregular defects with in situ UV crosslinking. In rabbit knee cartilage defects, BMSC‐loaded LBG‐MA hydrogels accelerated healing after 8 weeks, forming hyaline cartilage‐like tissue with smooth surfaces and good integration (Figure 12F). Histological scoring and biochemical analysis showed superior repair quality versus controls (*p* < 0.001), indicating excellent in vivo chondrogenic capacity.

LBG‐MA degradation rate is controlled by crosslinking density. Appropriate degradation is crucial—too rapid causes premature failure; too slow hinders regeneration. Optimizing methacrylation degree tunes degradation within 4–12 weeks, matching cartilage regeneration timescales. Photocrosslinking properties suit 3D bioprinting and microfluidic fabrication for complex osteochondral structures. LBG‐MA composites with nanohydroxyapatite, growth factors, or extracellular vesicles further enhance repair capacity. Overall, LBG‐MA hydrogels provide a safe, effective photocrosslinkable, injectable, biodegradable scaffold for minimally invasive cartilage repair.

### 5.3 Microbial‐Derived Materials

Microbial‐derived materials are biopolymers produced through microbial fermentation or metabolism, including bacterial cellulose, microbial hyaluronic acid, polyhydroxyalkanoates (PHA), gellan gum, and xanthan gum. Compared to animal‐ and plant‐derived materials, they offer short production cycles, scalable fermentation, high batch consistency, high purity, and no pathogen contamination risk. Genetic engineering and fermentation optimization enable precise control of molecular weight, composition, and functional properties for customized material design. These materials exhibit good biocompatibility and biodegradability, meeting tissue engineering requirements. Microbial fermentation aligns with green biomanufacturing principles, utilizing agricultural waste and industrial by‐products cost‐effectively. In osteochondral tissue engineering, they are used for hydrogel scaffolds, nanofibrous networks, drug delivery systems, and 3D bioprinting inks. This section focuses on bacterial cellulose, microbial hyaluronic acid, polyhydroxyalkanoates, and gellan gum.

#### 5.3.1 Bacterial Fermentation Products

##### 5.3.1.1 Bacterial Cellulose

Bacterial cellulose (BC) is secreted by acetic acid bacteria such as Gluconacetobacter xylinus during fermentation. It shares the same β‐1,4‐glucan structure as plant cellulose but possesses higher purity exceeding 99%, crystallinity of 60%–90%, and degree of polymerization of 2000–6000. BC self‐assembles from nanofibers with diameters of 20–100 nm into a three‐dimensional network with high porosity above 90%, large specific surface area exceeding 200 m^2^/g, and excellent mechanical properties, including tensile strength of 200‐300 MPa and Young's modulus of 10–30 GPa, closely resembling the collagen fiber network of natural cartilage [260]. BC exhibits excellent biocompatibility, is free of endotoxins and immunogenicity, and has FDA approval for medical devices such as wound dressings.

BC application in osteochondral tissue engineering focuses on cartilage repair and osteochondral interface construction. Its high porosity and hydrophilicity, with water retention capacity reaching 99% facilitate nutrient diffusion and metabolic waste removal, supporting three‐dimensional chondrocyte culture and cartilage matrix secretion [261]. BC hydrogels maintain chondrocyte spherical morphology and chondrogenic phenotype, promoting type II collagen and aggrecan expression. BC degrades mainly through cellulase‐mediated enzymatic hydrolysis with a slow rate and in vivo half‐life of several months to years, suitable for long‐term tissue regeneration.

Functionalization strategies improve BC bioactivity and degradation, including surface modification with RGD peptides or hyaluronic acid, nanocomposites with hydroxyapatite or bioactive glass, and TEMPO oxidation. Bilayer scaffolds combining BC with hydroxyapatite—upper BC layer simulating cartilage and lower BC/HAp composite simulating subchondral bone—demonstrate good osteochondral repair in animal models [262]. BC's high mechanical strength suits load‐bearing cartilage repair or composites with flexible hydrogels for mechanically graded structures. BC also serves as a supporting material or reinforcing fiber in 3D bioprinting for complex osteochondral constructs.

##### 5.3.1.2 Microbial Hyaluronic Acid

Hyaluronic acid (HA), traditionally extracted from animal tissues, suffers from low purity, heterogeneous molecular weight, pathogen contamination risk, and cultural restrictions. Microbial fermentation using Streptococcus species has become the primary source of clinical‐grade HA, offering high purity (>95%), controllable molecular weight (0.1–3.0 MDa), absence of animal pathogens, batch consistency meeting GMP standards, and improved production yield (>10 g/L) through strain engineering [199].

Microbial HA is widely applied in osteochondral tissue engineering. High molecular weight HA (>1.0 MDa) exhibits excellent viscoelasticity and lubrication for osteoarthritis treatment. Chemically modified HA, such as methacrylated HA, forms photocrosslinkable hydrogels for cartilage defect filling and cell delivery. Its biodegradability via hyaluronidase‐mediated hydrolysis allows metabolic absorption, with costs only 1/3 to 1/2 of animal‐derived HA, demonstrating strong clinical translation potential [243].

**FIGURE 13:**
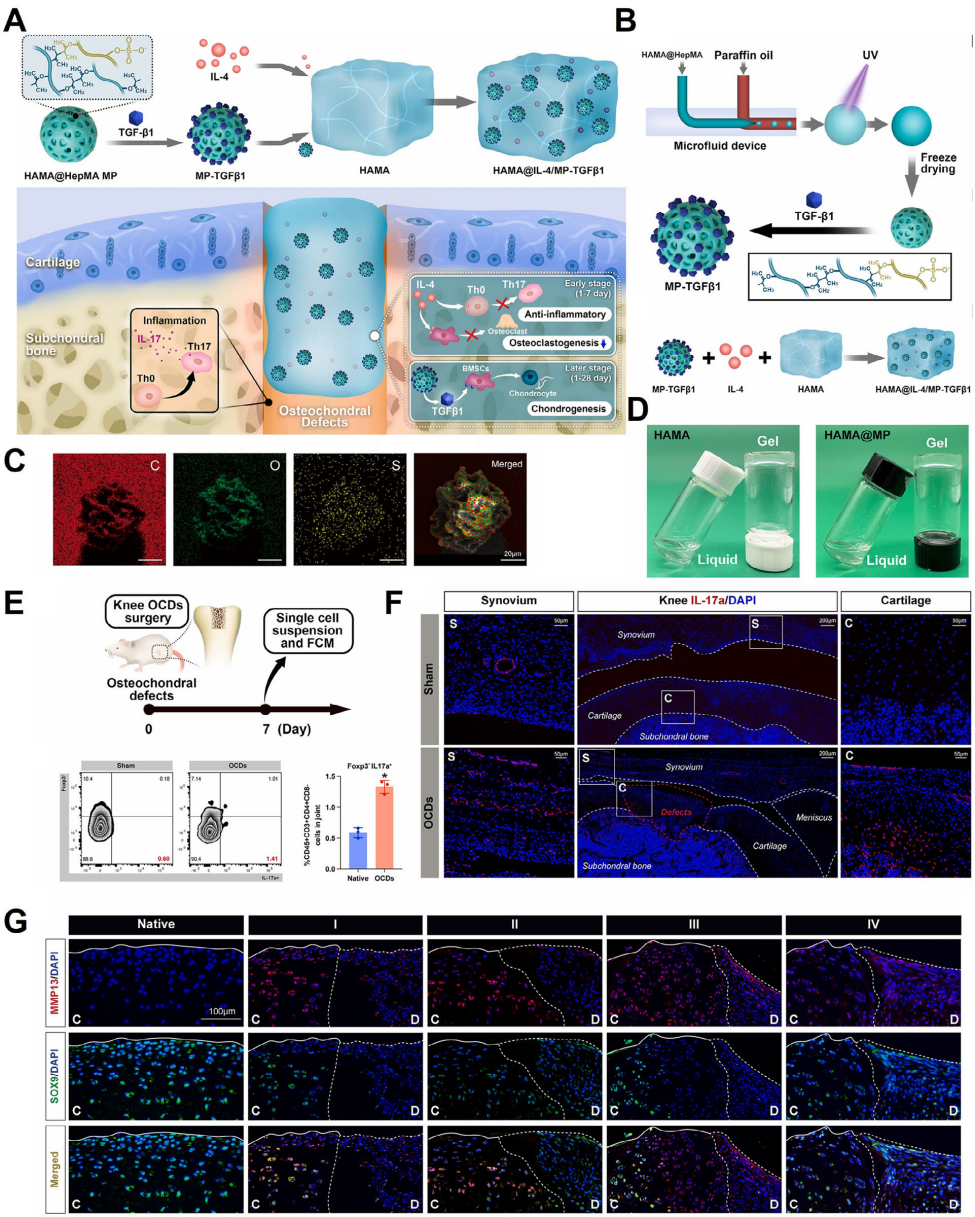
A novel sequential release hydrogel repairs osteochondral defects by modulating IL‐17‐mediated immune disorders. (A) Schematic illustration of the repair mechanism: HAMA@HepMA microparticles (MPs) were synthesized using a combination of microfluidics and photopolymerization techniques. Growth factor TGFβ1 was non‐covalently bound to heparin in MPs, while hyaluronic acid in MPs promoted cell ingrowth and adhesion. MPs and IL‐4 were encapsulated in HAMA hydrogels, where the initial release of IL‐4 corrected local immune imbalance, and the gradual release of TGFβ1 created a favorable environment for mesenchymal stem cell (MSC) chondrogenesis, ultimately promoting osteochondral defect regeneration. (B) Schematic of HAMA@HepMA microparticle synthesis via microfluidics. (C) Elemental mapping images of HAMA@HepMA microparticles obtained through energy‐dispersive spectroscopy analysis. (D) Photocuring performance of the HAMA@MP system. (E) Flow cytometry detection and multiparameter analysis of Foxp3^−^IL17a^+^ cells in the CD45^+^CD3^+^CD4^+^CD8^−^ population in rat OCD models. (F) Immunofluorescence staining of IL‐17a in synovium (S), cartilage (C), defect area, and subchondral bone. (G) Representative immunofluorescence images of SOX9 and MMP13 in OCDs regions, with dashed lines marking the boundary between defect (D) and surrounding cartilage (C). Data are presented as mean ± standard deviation (* indicates *p* < 0.05 compared to the specified group; ns indicates *p* > 0.05). Groups I to IV underwent OCD surgeries and received different treatments: Group I: PBS; Group II: HAMA@MP; Group III: HAMA@MP‐TGFβ1; Group IV: HAMA@IL‐4/MP‐TGFβ1. Reproduced under the terms of the CC BY‐NC‐ND 4.0 license [199]. Copyright 2025, The Author(s).

Multifunctional microbial HA‐based hydrogel systems show significant advantages in immunomodulation and tissue regeneration. Adaptive immune dysregulation in early osteochondral defects elevates Th17 cells and pro‐inflammatory IL‐17, exacerbating cartilage degradation [199]. Targeting this, recent research developed a sequential dual‐factor release system: methacrylated HA and heparin (HAMA@HepMA) microparticles prepared via microfluidics, co‐encapsulating IL‐4 and TGFβ1 in HAMA hydrogels (Figure 13A–D). Rapid IL‐4 release inhibits Th17 differentiation and corrects immune imbalance, while TGFβ1 achieves sustained release through heparin binding, promoting MSC chondrogenic differentiation.

In rat knee osteochondral defects, HAMA@IL‐4/MP‐TGFβ1 hydrogels significantly reduced Th17 cell proportion (*p* < 0.05) (Figure 13E), decreased IL‐17a expression in synovium and cartilage (Figure 13F), upregulated SOX9 and downregulated MMP13 in defect regions (Figure 13G), forming hyaline cartilage‐like tissue with good integration. This demonstrates that microbial HA hydrogel systems integrating immunomodulation and regenerative signals significantly enhance osteochondral repair, providing innovative strategies for orthopedic regenerative medicine.

Microbial HA offers controllable molecular weight, GMP compliance, avoidance of animal pathogen risk, and low production cost. It undergoes tunable enzymatic degradation with metabolically absorbable products. High molecular weight HA exhibits excellent viscoelasticity, and after modification forms photocrosslinkable hydrogels suitable for 3D bioprinting and injectable applications. HA mediates cell signaling via CD44 receptors, promoting migration, proliferation, differentiation, and possessing anti‐inflammatory and immunomodulatory effects [244, 245].

However, pure HA hydrogels have low compressive modulus (<10 kPa) versus native cartilage (0.5–2 MPa), requiring composites for mechanical support. Degradation rates influenced by hyaluronidase, molecular weight, and crosslinking are difficult to precisely match tissue regeneration timescales. Pure HA lacks specific osteogenic and chondrogenic signals, requiring growth factors or drugs to enhance regeneration. Multifunctional HA hydrogel fabrication is complex with high costs, and maintaining growth factor stability and bioactivity remains challenging, requiring formulation and process optimization for scaled production and clinical application.

##### 5.3.1.3 Polyhydroxyalkanoates

Polyhydroxyalkanoates (PHA) are intracellular energy storage polymers synthesized by bacteria such as Pseudomonas, Bacillus, and Rhodococcus under nutrient‐limiting conditions, representing fully biodegradable thermoplastic polyesters [202]. The PHA family includes PHB, PHBV, and PHO, with tunable composition and molecular weight through strain selection and fermentation optimization.

In osteochondral tissue engineering, PHA constructs porous scaffolds, nanofibers, and composites. Through salt leaching, freeze–drying, electrospinning, or 3D printing, PHA scaffolds achieve 60%–90% porosity with interconnected pores, supporting cell adhesion, proliferation, and differentiation [201]. Degradation rate is tunable via monomer composition—PHB degrades slowly, PHBV rapidly—with periods from weeks to years. PHA scaffolds support MSC osteogenic and chondrogenic differentiation, promoting osteochondral regeneration.

Functionalization strategies enhance bioactivity through surface modification, nanocomposites with hydroxyapatite or bioactive glass, and blending with collagen, gelatin, or chitosan. PHBV/hydroxyapatite composites demonstrate good osseointegration and cartilage repair in rabbit models. PHA's thermoplastic properties enable FDM 3D printing for patient‐specific scaffolds. PHA production from agricultural waste or industrial by‐products aligns with circular economy principles.

##### 5.3.1.4 Gellan Gum

Gellan gum (GG) is an anionic polysaccharide produced by Sphingomonas elodea fermentation, composed of glucose, glucuronic acid, and rhamnose in linear tetrasaccharide repeating units. High acyl GG (HA‐GG) forms soft elastic gels, while low acyl GG (LA‐GG) forms hard, transparent gels. Gelation is induced by cations such as Ca^2^^+^, Mg^2^^+^, and K^+^, with tunable properties via ion type, concentration, and acyl content [197, 200].

In osteochondral tissue engineering, LA‐GG gels exhibit a compressive modulus of 10–100 kPa comparable to natural cartilage and good shear‐thinning characteristics, suitable for injectable hydrogels and 3D bioprinting. Recent research developed a GG‐based bilayer hydrogel: upper LA‐GG/HA‐GG layer mimicking cartilage and lower LA‐GG/hydroxyapatite layer mimicking subchondral bone, achieving interfacial bonding strength of 30–50 kPa through ion diffusion [200]. In rabbit knee defects, this hydrogel promoted stratified tissue regeneration within 8 weeks, forming hyaline cartilage‐like tissue and subchondral bone with good interfacial integration.

**FIGURE 14:**
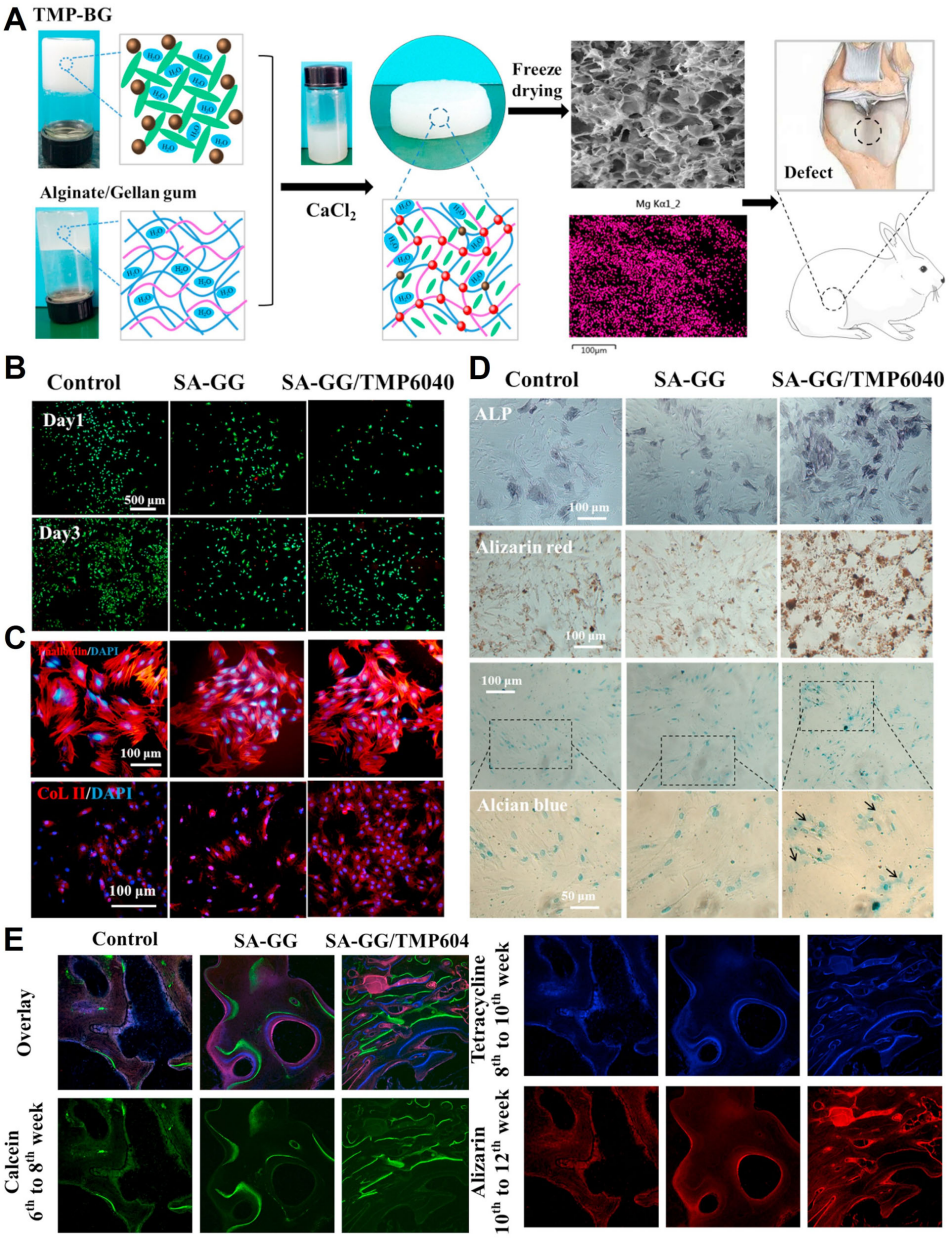
Preparation of SA‐GG/TMP‐BG hybrid hydrogel scaffold and its implantation in rabbit knee osteochondral defects. (A) Schematic illustration of the fabrication of SA‐GG/TMP‐BG hybrid hydrogel scaffold and its implantation in rabbit knee osteochondral defect models. Energy‐dispersive spectroscopy (EDS) elemental mapping of Mg indicates uniform distribution of inorganic TMP‐BG within the scaffold. (B, C) Live/dead cell staining and morphological observation of BMSCs co‐cultured with the hydrogel for 3 days. (D) Results of ALP staining, Alizarin Red staining, and Alcian Blue staining, with the control group containing only the medium. (E) Fluorescent labeling results of regenerated subchondral bone 12 weeks after implantation. Reproduced under the terms of the CC BY‐NC‐ND 4.0 license [200] Copyright 2022, The Author(s).

To address the challenge of simultaneous cartilage and subchondral bone regeneration in osteochondral defect repair, researchers developed a sodium alginate‐gellan gum/thixotropic magnesium phosphate‐based gel (SA‐GG/TMP‐BG) hybrid hydrogel system [200]. This system uniformly disperses inorganic TMP‐BG (primarily composed of MgNa_3_H(PO_4_)_2_ nanoparticles) within the SA‐GG hydrogel matrix through Mg^2^^+^ pre‐crosslinking (Figure 14A). Energy‐dispersive X‐ray spectroscopy (EDS) elemental mapping shows uniform Mg element distribution, confirming good compositing of the inorganic and organic phases. The SA‐GG/TMP‐BG hybrid hydrogel possesses tunable rheological properties, injectability, and mechanical properties, with its shear‐thinning characteristics making it suitable for minimally invasive injection and 3D bioprinting.

In vitro experiments showed that after 3 days of co‐culture of bone marrow mesenchymal stem cells (BMSCs) with SA‐GG/TMP‐BG hydrogel, live/dead cell staining demonstrated high cell viability, with cells exhibiting good adhesion and spreading on the hydrogel surface (Figure 14B,C). Alkaline phosphatase (ALP) staining, Alizarin Red staining, and Alcian Blue staining results indicated that SA‐GG/TMP‐BG hydrogel significantly promoted osteogenic and chondrogenic differentiation of BMSCs, with upregulation of related gene expression (Figure 14D). Mechanistic studies revealed that Mg^2^^+^ released from TMP‐BG contributes to BMSC recruitment and promotes their osteogenic and chondrogenic differentiation.

In a rabbit knee osteochondral defect model, SA‐GG/TMP6040 hydrogel with 60% TMP‐BG content showed significant subchondral bone regeneration after 12 weeks, with newly formed bone well‐integrated with surrounding tissue (Figure 14E). Histological evaluation revealed a stratified structure: upper hyaline cartilage‐like tissue, lower dense subchondral bone tissue, with natural interfacial transition. This demonstrates that inorganic TMP‐BG endows the hybrid hydrogel with excellent osteogenic activity, preferentially promoting subchondral bone regeneration and providing mechanical support for subsequent cartilage repair, key to successful osteochondral repair. Gellan gum biodegradability through enzymatic and hydrolytic degradation allows tunable degradation within 4–12 weeks, matching tissue regeneration timescales. Its low endotoxin content (<10 EU/g) and good biocompatibility provide promising clinical translation prospects.

#### 5.3.2 Fungal‐Derived Materials

Fungal cell walls and mycelial structures provide novel biomaterials for osteochondral tissue engineering, primarily including fungal chitosan and mycelial scaffolds, with advantages such as renewability, sustainability, and absence of animal‐derived pathogen contamination.

##### 5.3.2.1 Fungal Chitosan

Fungal chitosan is a natural polysaccharide from fungal cell walls, primarily sourced from Mucor and Rhizopus, with a higher deacetylation degree (70%–95%), molecular weight of 10‐1000 kDa, and stronger bioactivity than crustacean‐derived chitosan. In osteochondral tissue engineering, fungal chitosan is allergen‐free with superior biocompatibility and sustainable production via short fermentation cycles (3–7 days) without seasonal limitations. Its cationic surface promotes cell adhesion, supports MSC chondrogenic differentiation, upregulates SOX9, COL2A1, and ACAN expression, and exhibits antibacterial and anti‐inflammatory activities by modulating macrophage M2 polarization [263].

Applications include injectable hydrogels for irregular defects and chitosan/hydroxyapatite composite scaffolds with 5‐15 MPa compressive modulus, 60%–80% porosity, and 100–300 µm pore sizes, facilitating cell migration and vascularization. In rabbit knee defects, Kartogenin‐loaded fungal chitosan hydrogel formed hyaline cartilage‐like tissue after 8 weeks with increased GAG and type II collagen expression [264].

Limitations include low mechanical strength (<1 MPa) requiring composites or modification, acid‐only solubility needing quaternization or carboxymethylation, and batch consistency requiring optimization.

##### 5.3.2.2 Mycelial Scaffolds

Mycelial scaffolds are biomaterials from fungal mycelia of Ganoderma and Pleurotus, prepared by inoculating spores onto agricultural waste substrates, culturing 7–14 days, then heat or chemical inactivation. They possess 70%–90% porosity, 10–500 µm pore sizes, and highly interconnected structures. Main components include chitin (30%–50%), β‐glucan, proteins, and polysaccharides, with compressive moduli of 0.1–5 MPa and tensile strength of 0.5–3 MPa [265].

Applications in osteochondral repair are limited due to insufficient mechanical properties for load‐bearing joints and slow degradation. However, alkaline treatment converting chitin to chitosan improves hydrophilicity and cell adhesion. Chondrocytes show good adhesion and proliferation, with scaffold β‐glucan activating cell receptors to promote signaling and matrix synthesis.

Mycelial/hydroxyapatite composites prepared by adding calcium phosphate precursors to culture medium utilize mycelial mineralization for in situ nano‐hydroxyapatite generation, achieving 10–20 MPa compressive modulus approaching cancellous bone with good osteoconductivity. Mycelial scaffolds offer low cost and sustainability by utilizing agricultural waste [266]. However, insufficient mechanical properties, singular bioactivity, and lack of specific chondrogenic and osteogenic signals require growth factor or drug loading. Clinical safety and efficacy need extensive validation.

Overall, fungal chitosan demonstrates promising prospects as a crustacean‐chitosan alternative in osteochondral repair. Although mycelial scaffold applications are limited, their unique structure and environmental characteristics represent an important future research direction.

## 6 Future Directions: Smart Natural Materials

With the rapid development of materials science, biotechnology, and nanotechnology, osteochondral tissue engineering is advancing toward intelligent, precise, and personalized directions. Smart natural materials can sense and respond to changes in the physiological microenvironment, enabling dynamic regulation of cell behavior and tissue regeneration processes. Stimulus‐responsive hydrogels, piezoelectric materials with mechanotransduction regulation, and the integration of 3D printing with natural materials represent cutting‐edge directions in osteochondral tissue engineering, providing novel strategies and technological platforms for achieving functional tissue regeneration.

### 6.1 Stimulus‐Responsive Hydrogels

Stimulus‐responsive hydrogels are a class of smart materials capable of sensing external or internal environmental stimuli and undergoing reversible physicochemical changes. These stimuli include temperature, pH, light, magnetic field, enzymatic activity, redox state, and others. Stimulus‐responsive hydrogels possess unique advantages in osteochondral tissue engineering, enabling controlled drug release, dynamic regulation of cell behavior, and spatiotemporal precise control of tissue regeneration.

#### 6.1.1 Temperature‐Responsive Hydrogels

Temperature‐responsive hydrogels are the most common stimulus‐responsive materials, typically based on poly(N‐isopropylacrylamide) (PNIPAM) or its derivatives. These hydrogels possess a lower critical solution temperature (LCST), existing in a solution state at room temperature and rapidly gelating upon heating to body temperature (37°C). Temperature‐responsive hydrogels can be combined with natural materials (such as gelatin, hyaluronic acid, chitosan) to form composite hydrogels with good biocompatibility and injectability [234]. In osteochondral repair, temperature‐responsive hydrogels can be loaded with mesenchymal stem cells and growth factors, implanted into defect sites through minimally invasive injection, and undergo in situ gelation to form three‐dimensional scaffolds that support cell adhesion, proliferation, and differentiation [267]. Studies have shown that PNIPAM‐gelatin composite hydrogels loaded with BMSCs and TGF‐β3, when implanted for 12 weeks in a rabbit knee osteochondral defect model, resulted in the formation of hyaline cartilage‐like tissue in the repair region with mechanical properties approaching those of native cartilage.

Temperature‐responsive hydrogels can also achieve spatiotemporal controlled drug release. By modulating the LCST and crosslinking density of the hydrogel, drug release rate and duration can be controlled. For example, temperature‐sensitive nanoparticles encapsulating Kartogenin dispersed in temperature‐responsive hydrogels slowly release the drug at body temperature, with sustained release lasting 4–6 weeks, significantly promoting cartilage regeneration [264]. Additionally, temperature‐responsive hydrogels can be combined with magnetic nanoparticles to achieve on‐demand drug release through external magnetic field heating, providing a new avenue for precision therapy.

#### 6.1.2 PH‐Responsive Hydrogels

pH‐responsive hydrogels utilize pH changes under physiological or pathological conditions to achieve smart responses. Arthritis and osteochondral injury sites typically present an acidic microenvironment (pH 5.5–6.5), while normal tissue pH is approximately 7.4. pH‐responsive hydrogels usually contain ionizable groups (such as carboxyl and amino groups) that undergo protonation or deprotonation under different pH conditions, leading to hydrogel swelling or contraction [268]. Natural polysaccharides such as chitosan, hyaluronic acid, and alginate, which contain abundant carboxyl or amino groups, are ideal materials for constructing pH‐responsive hydrogels.

pH‐responsive hydrogels can achieve inflammation‐targeted drug release. In the early stage of osteochondral injury, inflammatory responses lead to local pH reduction, and pH‐responsive hydrogels swell or degrade in acidic environments, accelerating the release of anti‐inflammatory drugs (such as dexamethasone and celecoxib) to suppress inflammatory responses. As inflammation subsides and pH gradually returns to normal, the hydrogel contracts and drug release slows down, avoiding delayed tissue repair caused by excessive anti‐inflammatory effects [269]. Studies have shown that pH‐responsive chitosan/alginate hydrogels loaded with dexamethasone exhibit drug release rates under acidic conditions (pH 6.0) that are 3‐5 times higher than under neutral conditions (pH 7.4), significantly improving osteochondral repair outcomes.

#### 6.1.3 Light‐Responsive Hydrogels

Light‐responsive hydrogels achieve reversible or irreversible property changes through light irradiation, offering high spatiotemporal resolution and non‐invasiveness. Photoresponsive groups (azobenzene, coumarin, spiropyran) undergo conformational changes or chemical reactions under specific wavelength light, altering hydrogel crosslinking density, mechanical properties, and degradation rate [246]. Light‐responsive hydrogels can be chemically modified with natural materials (gelatin, hyaluronic acid) to form composite hydrogels with photoregulatory functions.

In osteochondral tissue engineering, light‐responsive hydrogels enable spatiotemporal precise control of cell behavior. Localized light irradiation modulates the mechanical properties of specific hydrogel regions to guide directional cell differentiation. In bilayer scaffolds, UV irradiation of the upper cartilage layer reduces crosslinking density, creating a softer microenvironment (10–100 kPa modulus) promoting chondrogenic differentiation, while visible light irradiation of the lower bone layer increases crosslinking density, creating a harder microenvironment (1–10 MPa modulus), promoting osteogenic differentiation [270]. Additionally, light‐responsive hydrogels achieve on‐demand drug release by triggering degradation or pore opening through light irradiation, enabling pulsatile drug release.

#### 6.1.4 Enzyme‐Responsive Hydrogels

Enzyme‐responsive hydrogels utilize tissue‐specific enzymatic activity for smart responses. During osteochondral injury and repair, enzymes such as matrix metalloproteinases (MMPs), hyaluronidase, and collagenase exhibit significant expression and activity changes. Enzyme‐responsive hydrogels contain enzyme‐specific cleavage sites and degrade under enzymatic action, releasing encapsulated growth factors or drugs [271]. These hydrogels achieve dynamic tissue regeneration regulation: rapid early degradation releases pro‐regenerative factors, while slow degradation during tissue maturation maintains mechanical support.

Studies show that gelatin hydrogels containing MMP‐2 cleavage sites at osteochondral defects undergo gradual degradation as MMP‐2 expression increases with cell migration and matrix remodeling, releasing loaded BMP‐2 and TGF‐β3 to promote synchronized subchondral bone and cartilage regeneration [272]. The degradation rate matches tissue regeneration rate, avoiding mechanical mismatch and regeneration obstacles caused by excessively fast or slow degradation of traditional hydrogels.

#### 6.1.5 Multi‐Responsive Hydrogels

Multi‐responsive hydrogels integrate multiple stimulus‐responsive mechanisms to achieve more precise smart regulation [273]. For example, temperature‐pH dual‐responsive hydrogels combine temperature sensitivity and pH sensitivity, gelating at body temperature and accelerating drug release in acidic inflammatory microenvironments. Light‐temperature dual‐responsive hydrogels achieve light‐controlled temperature elevation through photothermal conversion nanomaterials (such as gold nanorods, graphene oxide), triggering temperature‐responsive drug release [274]. Multi‐responsive hydrogels provide more flexible regulatory strategies for osteochondral tissue engineering, but their design and fabrication complexity is relatively high and requires further optimization.

### 6.2 Piezoelectric Materials and Mechanoelectrical Signal Regulation

Piezoelectric materials convert mechanical stress into electrical signals, providing biomimetic physical stimulation for osteochondral tissue engineering [275]. Bone and cartilage possess natural piezoelectric properties, with collagen having a piezoelectric coefficient of ∼0.2–2 pC/N [276]. During joint movement, mechanical stress generates charge separation and electric fields that regulate cell behavior and tissue regeneration.

Natural piezoelectric materials include collagen (1–2 pC/N), chitosan (0.5–1.5 pC/N), and cellulose nanocrystals (2–3 pC/N), but their low piezoelectric coefficients cannot meet osteochondral repair needs (cartilage layer ∼25 mV, bone layer 40–80 mV). Synthetic materials like PZT (300–600 pC/N) have high coefficients but contain toxic lead; BaTiO_3_ is lead‐free but poorly degradable; PVDF requires high‐voltage polarization and is non‐degradable [277].

**FIGURE 15:**
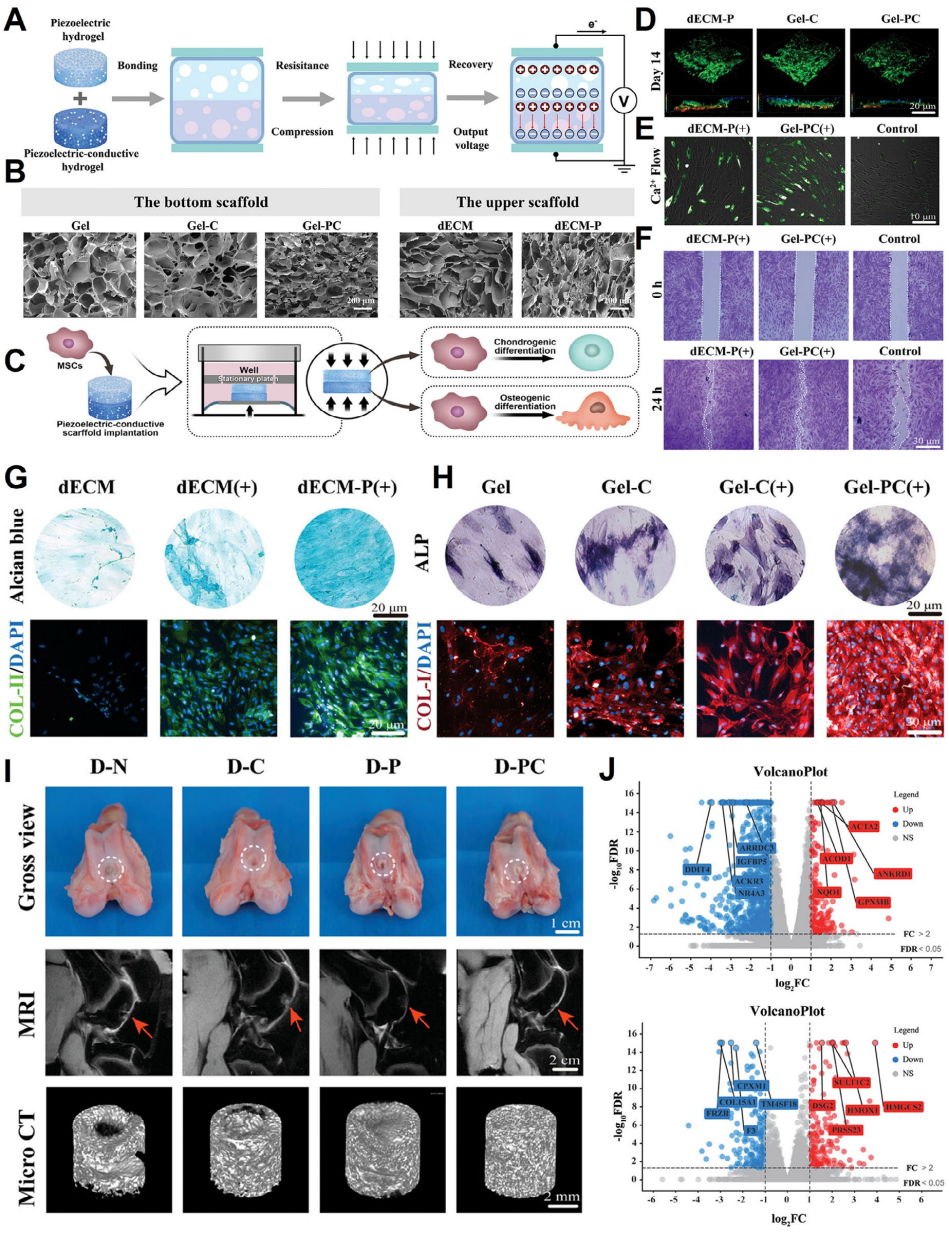
Schematic and results of a piezoelectric conductive hydrogel for osteochondral repair. (A) Schematic illustration of the composition and properties of the hydrogel. (B) Scanning electron microscopy (SEM) images showing the hydrogel microstructure. (C) Schematic of the in vitro study design. (D) Representative confocal laser scanning microscopy (CLSM) images showing cell viability after 14 days of co‐culture with the scaffold. (E) Comparison of Ca^2^^+^ influx under strong electrical stimulation versus the control group. (F) Comparison of cell migration under force‐electric stimulation versus the control group. (G) Alcian Blue staining and COL‐II immunofluorescence, and 2.5D immunofluorescence views of dECM, dECM(+), and dECM‐P(+) groups. (H) ALP staining and COL‐I immunofluorescence, and 2.5D immunofluorescence views of Gel, Gel‐C, Gel‐C(+), and Gel‐PC(+) groups. (I) Representative evaluations at 6 months post‐surgery, including gross appearance, MRI scans, and 3D micro‐CT reconstructions. (J) Differentially expressed genes (DEGs) in dECM‐P(+) and Gel‐PC(+) groups compared to controls (FC > 2 and FDR < 0.05). Reproduced with permission [278]. Copyright 2024, Wiley.

Biocompatible piezoelectric materials have emerged as research hotspots. Diphenylalanine (FF) self‐assembles into hexagonal structures with d_33_ of 18 pm/V, meeting electrical stimulation needs with excellent biocompatibility and degradability. Liu et al. [278] developed a piezoelectric‐conductive composite scaffold with an upper piezoelectric cartilage dECM layer and lower piezoelectric‐conductive modified gelatin (Gel‐PC) layer (Figure 15A). FF peptide modification conferred piezoelectricity, while PEDOT incorporation enhanced conductivity. The scaffold has interconnected porous structure (100–300 µm pores), facilitating cell migration (Figure 15B).

In vitro experiments showed electric fields activate voltage‐gated calcium channels, increasing intracellular Ca^2^^+^ (Figure 15E) and activating calcium‐dependent signaling pathways. Mechanoelectrical stimulation enhanced BMSC migration (Figure 15F), with positive charges promoting chondrogenic differentiation and negative charges promoting osteogenic differentiation. After 14 days, cell viability exceeded 90% (Figure 15D). GAG and type II collagen expression in dECM‐P(+) group were significantly elevated (Figure 15G), and ALP activity and type I collagen in Gel‐PC(+) group increased significantly (Figure 15H).

In a Parma pig knee defect model (8 mm diameter), at 6 months the piezoelectric‐conductive scaffold group showed smooth surface with good integration (Figure 15I). MRI revealed cartilage signal approaching normal levels, and micro‐CT showed good subchondral bone regeneration. Transcriptomic analysis showed differentially expressed genes (FC > 2, FDR < 0.05) enriched in cartilage development, bone formation, and ECM organization pathways (Figure 15J).

The working mechanism relies on joint movement‐generated mechanical stress. Compression generates electrical potential differences: positive charges (∼25 mV) in upper layer promote chondrogenic differentiation, negative charges (40–80 mV) in the lower layer promote osteogenic differentiation. PEDOT reduces electrical resistance, compensating for insufficient piezoelectric output from smaller lower‐layer deformation.

Challenges include low piezoelectric coefficients of natural materials, optimization needs for synthetic material biocompatibility and degradability, polarization stability requirements, and mechanism investigation needs. Integration of piezoelectric materials with stimulus‐responsive hydrogels and conductive materials will create multimodal physical stimulation platforms. By mimicking the native osteochondral mechanoelectrical coupling microenvironment, piezoelectric‐conductive composite scaffolds demonstrate substantial clinical application potential.

Notably, naturally derived piezoelectric materials, including collagen, chitosan, and cellulose nanocrystals, offer inherent advantages over synthetic counterparts in terms of biocompatibility, biodegradability, and the ability to recapitulate the native osteochondral mechanoelectrical microenvironment. These features align more closely with the biological‐origin‐centered focus of this review, and future efforts to enhance their piezoelectric output through structural modification or hybrid strategies may further unlock their potential as biomimetic platforms for osteochondral regeneration.

### 6.3 Integration of 3D Printing With Natural Materials

3D printing technology enables precise fabrication of complex scaffolds for osteochondral tissue engineering with personalized customization and multi‐material gradient distribution [22, 248]. Digital light processing (DLP) bioprinting offers high precision (10–50 µm), rapid prototyping, and good cell viability, but requires photosensitive bioinks necessitating photosensitization modification of natural materials. Type II collagen (Col II), the major cartilage ECM structural protein crucial for tensile strength and tissue integrity, provides biochemical and structural support for chondrocytes, maintaining their phenotype and promoting hyaline cartilage formation [279]. However, naturally low Col II content and complex extraction processes for high‐purity, non‐denatured Col II limit its widespread application in cartilage tissue engineering.

**FIGURE 16:**
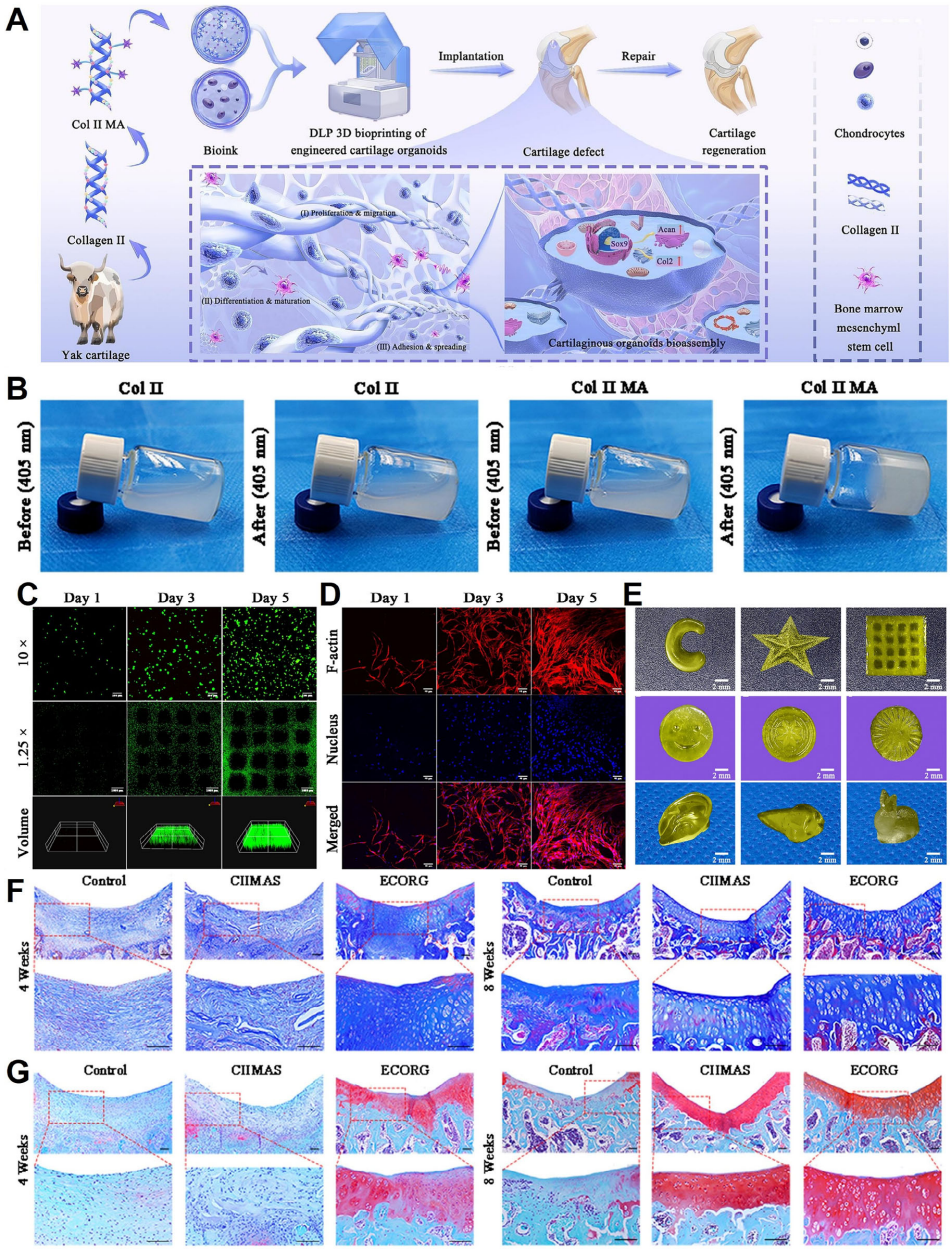
A novel DLP 3D bioprinting strategy utilizing high‐purity, non‐denatured type II collagen derived from yak cartilage for engineering cartilage organoids in osteoarthritis treatment. (A) Schematic illustration of the preparation of high‐purity, non‐denatured type II collagen and DLP 3D bioprinting using a novel bioink composed of type II collagen methacrylate (Col II MA) and chondrocytes for repairing cartilage defects and engineering cartilage organoids. (B) Morphology of Col II MA and Col II ink before and after light exposure. (C) Live/dead cell staining of chondrocytes after 1, 3, and 5 days of culture in engineered cartilage organoids (ECORG). (D) Immunofluorescence staining of chondrocytes cultured in ECORG. (E) Various shapes fabricated by DLP 3D printing using Col II MA ink. (F) Masson staining at 4 and 8 weeks post‐implantation. (G) Saf‐O/FG staining at 4 and 8 weeks post‐implantation. Reproduced with permission [279]. Copyright 2025, Elsevier.

To overcome this limitation, Yang et al. [279] extracted high‐purity, non‐denatured Col II from yak cartilage and chemically functionalized it into photoreactive type II collagen methacrylate (Col II MA) for DLP 3D bioprinting of engineered cartilage organoids (ECORG) (Figure 16A). Col II MA exists in a liquid state before light exposure and rapidly crosslinks upon illumination to form a stable hydrogel structure (Figure 16B), possessing excellent mechanical integrity, minimal swelling, and controllable enzymatic degradability. After mixing Col II MA bioink with chondrocytes, scaffolds with various complex shapes can be printed (Figure 16E), including anatomical structures such as ears and noses, demonstrating the high precision and design flexibility of DLP printing.

In vitro experiments demonstrated excellent ECORG cell compatibility. Live/dead staining showed chondrocyte viability >90% after 1, 3, and 5 days with uniform distribution (Figure 16C). Immunofluorescence revealed chondrocytes expressed type II collagen and aggrecan, indicating ECORG maintained chondrocyte phenotype and promoted cartilage matrix synthesis (Figure 16D). ECORG also promoted BMSC chondrogenic differentiation with significant upregulation of SOX9, COL2A1, and ACAN expression.

In a rat cartilage defect model, Masson staining at 4 and 8 weeks showed ECORG group formed dense collagen fiber networks with good integration with native cartilage (Figure 16F). Safranin O/Fast Green staining revealed significantly higher proteoglycan content in the ECORG group, with neocartilage structure and biochemical properties approaching native cartilage (Figure 16G). At 8 weeks, repair tissue showed a smooth surface without fibrosis, with round chondrocytes uniformly distributed in lacunae, indicating functional hyaline cartilage formation.

DLP 3D bioprinting combined with Col II MA bioink offers: (1) high biomimicry providing natural biochemical signals; (2) excellent mechanical properties from photocrosslinked networks; (3) high printing precision for microstructure control; (4) good cell compatibility, maintaining high viability. However, single‐layer scaffolds are insufficient for osteochondral defects, requiring gradient‐structured scaffolds. Multi‐nozzle systems can simultaneously print different bioinks—upper layer Col II MA‐chondrocytes, lower layer GelMA‐hydroxyapatite‐osteoblasts, with an intermediate transition layer gradually changing ratios to form continuous gradient structures mimicking native osteochondral interface.

Challenges remain: natural material bioinks need enhanced mechanical properties to withstand physiological loads; printing precision, speed, and cell viability require optimization to reduce shear stress; vascularization and innervation remain unresolved [22]. Future 4D printing technology will enable scaffolds to dynamically change shape, structure, or function in response to physiological stimuli (temperature, pH, mechanical stress) for adaptive regeneration. Integrating 3D printing with piezoelectric materials, stimulus‐responsive systems, and growth factor delivery will construct multifunctional smart scaffolds, advancing clinical translation of osteochondral tissue engineering.

## 7 Clinical Translation Pathway of Natural Materials

Natural materials have demonstrated excellent biocompatibility and regenerative potential in osteochondral tissue engineering, but the path from laboratory research to clinical application still faces stringent regulatory approval, clinical trial validation, and commercialization challenges. This chapter systematically analyzes the regulatory classification of natural material products, successful cases approved by FDA/EMA, lessons learned from clinical trial successes and failures, the impact of regulatory policies on material selection, and the translation pathway from laboratory to clinic, providing a reference for the clinical translation of natural materials.

### 7.1 Regulatory Classification and Approval Pathways

The clinical translation of natural biomaterial products requires clarification of their regulatory classification, as different classifications determine approval pathways, clinical trial requirements, and time to market. The U.S. FDA and European EMA are the world's primary regulatory agencies, and their classification systems have a decisive influence on natural material product development [280].

The FDA classifies medical devices into three risk‐based classes: Class I (low‐risk), Class II (moderate‐risk), and Class III (high‐risk). Most natural material osteochondral repair products are classified as Class III devices or biological products, requiring rigorous Premarket Approval (PMA) with complete clinical trial data. However, some products can simplify approval through HCT/P (Human Cells, Tissues, and Cellular and Tissue‐Based Products) classification [281].

HCT/P products are classified into two categories: 361 HCT/P and 351 HCT/P. Products meeting 361 HCT/P criteria must undergo only minimal manipulation (washing, cutting, freezing, or decellularization), be used for homologous purposes, not be combined with other substances, and not rely on cellular metabolic activity. These products require only FDA registration and Good Tissue Practice (GTP) compliance, significantly reducing time and costs. In contrast, 351 HCT/P products require PMA approval as biologics with complete clinical trial data. For example, the particulated juvenile articular cartilage product DeNovo NT successfully obtained market authorization through the 361 HCT/P pathway, while autologous chondrocyte products containing living cells (MACI, Carticel) required the more rigorous 351 HCT/P pathway [282].

Class II devices can utilize the 510(k) pathway by demonstrating substantial equivalence to a predicate device, typically requiring 6–12 months without clinical trials. For instance, Chondro‐Gide obtained 510(k) approval using an existing collagen membrane as the predicate device [283]. The PMA pathway for Class III devices requires randomized controlled trials, long‐term follow‐up (≥2 years), and multi‐center studies, typically taking 1–3 years with costs ranging from millions to tens of millions of dollars [284].

The European CE marking system, governed by the Medical Device Regulation (MDR) and reviewed by third‐party Notified Bodies, typically offers faster approval with less stringent clinical data requirements than the FDA. Consequently, many natural material products first obtain CE marking in Europe to accumulate clinical data before applying for FDA approval. For example, Chondro‐Gide obtained CE marking in 2000 and subsequently received FDA 510(k) approval in 2010 [285].

### 7.2 Case Analysis of Successfully Marketed Natural Material Products

Since 2000, 47 osteochondral repair products have been approved for marketing globally by the FDA or EMA, providing rich practical experience for the clinical translation of natural materials. Based on material source and composition, these products can be categorized into allogeneic sources (14 products), non‐allogeneic sources (12 products), composite sources (12 products), natural/synthetic material composites (5 products), and synthetic materials only (4 products) (Table 6). Natural materials dominate osteochondral repair products, with allogeneic and non‐allogeneic source products becoming mainstream in clinical applications due to their excellent biocompatibility and regenerative potential. The following sections analyze the successful experiences and challenges of cartilage repair, bone repair, and decellularized matrix products through specific product case studies.

**TABLE 6:** Osteochondral Repair Products Commercially Available Worldwide Since 2000.

Category	Trade name	Launch time	Material characteristics	Cells/Factors	Number of Surgeries	Reference
Human‐derived	Chondron	2001	Fibrin glue mixed with autologous chondrocytes	AC/–	2	[303]
	DeNovo NT	2007	Particulated juvenile articular cartilage transplantation (donors ≤ 13 years old)	AC/–	1	[304]
	ChondroCelect	2009	Autologous chondrocytes only	AC/–	2	[305]
	BioCartilage	2012	Made from allogeneic cartilage, serving as a scaffold for microfracture defects	–/–	1	[245]
	HiQCell	2014	Autologous adipose‐derived mesenchymal stem cells	ADMSCs/–	2	[306]
	Cartiform	2017	Allogeneic osteochondral graft transplantation	–/–	1	[307]
	Spherox	2017	Autologous chondrocytes (spheroids), representing fourth‐generation autologous chondrocyte implantation technology	AC/‐	2	[308]
	Cartigrow	2017	Fibrin glue and autologous chondrocytes	AC/–	2	[309]
	Cellistem‐OA	2018	Allogeneic umbilical cord‐derived mesenchymal stem cells	UCMSCs/–	2	[310]
	CartiLife	2019	Costal cartilage‐derived chondrocytes cultured into three‐dimensional spherical microbeads	AC/–	2	[311]
	CartiMax	2021	Allogeneic moldable cartilage matrix capable of conforming to cartilage defects of various shapes and sizes. No shaping, trimming, suturing, or gluing required.	AC/–	1	[312]
	Prochondrix	2022	Laser‐etched, cryopreserved osteochondral allograft	–/–	1	[181, 313]
	RevaFlex(DeNovo ET)	2024	Scaffold‐free tissue‐engineered cartilage construct cultured from juvenile allogeneic chondrocytes	–/–	1	[314]
	BioDRestore	Phase III ongoing	Flowable dehydrated amniotic membrane allograft	–/–	1	[315]
Non‐human‐derived	AMIC Chondro‐Gide	2004	Combined with microfracture technique and porcine type I/III collagen membrane	–/–	1	[316]
	CartiFill	2007	Liquid porcine‐derived atelocollagen type I	–/–	1	[311]
	FibroFix	2008	This product features a porous tissue‐regenerating silk protein scaffold design, aimed at promoting cartilage tissue repair and regeneration.	–/–	1	[249]
	Hyalofast	2009	Single‐stage, hyaluronic acid‐based cartilage repair scaffold	–/–	1	[317]
	CaReS‐1 Step (CaReS‐1S)	2010	Acellular rat type I collagen filling gel	–/–	1	[241]
	ChondroFillergel	2011	Increased rat type I collagen content (from 4.8 mg/mL to 10 mg/mL)	–/–	1	[318]
	BST‐Cargel	2012	First‐generation chitosan hydrogel product for enhancing cartilage repair outcomes in microfracture surgery	–/–	1	[237]
	ChondroFillerliquid (CF)	2013	Based on rat type I collagen gel, prepared as injectable collagen hydrogel through advanced processing	–/–	1	[319]
	HYALOFAST	2013	Hyaluronic acid‐based acellular three‐dimensional scaffold	–/–	1	[244]
	JointRep	2013	Improved version of BST‐CarGel (first‐generation chitosan hydrogel product)	–/–	1	[320]
	Cartimaix	2014	Biodegradable porcine‐derived type I/III collagen membrane	–/–	1	[321]
	COLTRIX	2015	Enzymatically treated porcine‐derived type I atelocollagen	–/–	1	[322]
Composite‐derived	ACI‐Maix	2000	Matrix‐induced autologous chondrocyte implantation	AC/–	2	[323]
	CaReS	2003	Rat tail type I collagen gel mixed with autologous chondrocytes	AC/–	2	[324]
	NovoCart 3D	2003	Equine‐derived type I/III collagen matrix mixed with autologous chondrocytes and FGF‐2	AC/FGF‐2	2	[325]
	ChondroArt 3D	2007	Autologous hyaline chondrocytes encapsulated in alginate‐agarose hydrogel	AC/–	2	[326]
	NovoCart Inject	2008	Autologous chondrocytes suspended in an injectable hydrogel system composed of albumin‐hyaluronic acid, supplemented with human serum and cell culture medium	AC/–	2	[327]
	JAAC	2012	Atelocollagen solution mixed with autologous chondrocytes (3% type I collagen)	AC/–	2	[328]
	BioCart II	2012	Fibrin, hyaluronic acid, and autologous chondrocytes	AC/–	2	[329]
	CARISTEM	2012	UCBMSCs + hyaluronic acid (approximately 7.5×10^6^ cells/vial)	UCBMSCs/–	1	[330]
	MACI	2013	Using porcine‐derived type I/III collagen membrane as autologous chondrocyte carrier	AC/–	2	[294]
	Ortho‐ACI	2017	Healthy autologous chondrocytes seeded on collagen scaffold	AC/–	2	[305]
	NeoCart	FDA‐RMAT (2022), not approved	Autologous chondrocytes with 3D bovine collagen honeycomb scaffold combined with bioreactor culture system, generating hyaline cartilage after implantation	AC/–	2	[331]
	Cartipatch	Phase III ongoing	Autologous chondrocytes suspended in agarose‐alginate composite hydrogel	AC/–	2	[332]
Naturally/Synthetically‐derived	BioSeed‐C	2007	PDS‐reinforced PGA‐PLA scaffold as autologous chondrocyte carrier	AC/–	2	[333]
	ChondroTissue	2007	PGA‐hyaluronic acid composite scaffold (can be combined with platelet‐rich plasma)	–/–	1	[334]
	Chondromimetic	2010	Collagen‐GAG‐calcium phosphate biphasic osteochondral scaffold	–/–	1	[335]
	MaioRegen	2010	Upper layer of pure collagen, middle layer of collagen‐hydroxyapatite transitioning to lower layer of magnesium‐enriched hydroxyapatite bone layer	–/–	1	[336]
	CAIS	2011	PDO‐reinforced minced cartilage‐PCL/PGA copolymer composite scaffold	AC/–	1	[337]
Synthetically‐derived	SaluCartilage	2002	Polyvinyl alcohol (PVA) cryogel prepared through freeze‐thaw cycling process	–/–	1	[338]
	TruFit CB	2008	Cartilage layer composed of PLGA, bone layer reinforced with calcium sulfate and PGA fibers	–/–	1	[299]
	INSTRUCT	2015	Made from poly(ethylene oxide terephthalate)/poly (butylene terephthalate) (PEOT/PBT) copolymer material	–/–	1	[339]
	Agili‐C	2022	Subchondral bone layer: pure coralline aragonite. Cartilage layer: coralline aragonite and hydroxyapatite	–/–	1	[340]

#### 7.2.1 Allogeneic Source Products

Allogeneic source products have demonstrated outstanding performance in the field of cartilage repair, with the most representative being DeNovo NT (Natural Tissue Graft) based on particulated juvenile articular cartilage (PJAC) [286]. DeNovo NT, developed by Zimmer Biomet, received FDA approval in 2007 through the 361 HCT/P pathway. The product obtains articular cartilage from donors aged 6 months to 13 years, which is washed and cut into 1–3 mm particles and preserved in physiological saline. Clinical studies of DeNovo NT showed that at 12 months post‐implantation, patient IKDC (International Knee Documentation Committee) scores improved from a preoperative 43.2 to 78.5 (*P* < 0.001), with MRI showing complete defect filling and cartilage signal approaching normal [287]. The advantages of DeNovo NT include: (1) juvenile chondrocytes have strong proliferative capacity without requiring in vitro expansion; (2) rich in growth factors and ECM components, promoting regeneration; (3) 361 HCT/P classification simplifies approval without requiring clinical trials. However, DeNovo NT relies on donor tissue, facing issues of donor shortage and batch‐to‐batch variability, which are common challenges for allogeneic source products. In addition to DeNovo NT, allogeneic cartilage transplantation products (such as BioCartilage, Cartiform) have similarly obtained approval through the 361 HCT/P pathway, providing diversified options for cartilage defect repair [288].

#### 7.2.2 Non‐Allogeneic Source Products

Non‐allogeneic source products primarily include animal‐derived materials, with porcine and bovine collagen products being widely used in cartilage repair. Chondro‐Gide is a bilayer porcine type I/III collagen membrane developed by Geistlich, which obtained CE marking in 2000 and FDA 510(k) approval in 2010 [289]. Chondro‐Gide is used as a covering membrane for autologous chondrocyte implantation (ACI) and microfracture surgery, with its bilayer structure (compact layer and porous layer) mimicking the stratified structure of cartilage, promoting cell adhesion and cartilage matrix synthesis. Clinical studies have shown that microfracture surgery covered with Chondro‐Gide is superior to microfracture alone, with IKDC scores improving by 25‐30 points [290]. The success of Chondro‐Gide lies in: (1) stable material source (porcine collagen); (2) rapid market entry through the 510(k) pathway; (3) compatibility with existing surgical techniques (ACI, microfracture). Similar non‐allogeneic products include NOVOCART 3D (porcine type I collagen scaffold) [291], Maioregen (equine type I collagen‐hydroxyapatite composite scaffold), among others, which have gradually entered the U.S. market after obtaining CE marking in Europe [292]. The advantages of non‐allogeneic source products lie in abundant material supply and relatively low cost, but strict control of viral and prion transmission risks is required to meet FDA and EMA safety requirements for animal‐derived materials.

Composite source products combine the advantages of allogeneic and non‐allogeneic materials, enhancing mechanical properties and bioactivity through material composites. For example, MACI (Matrix‐induced Autologous Chondrocyte Implantation) is a third‐generation autologous chondrocyte implantation product developed by Vericel, which received FDA approval in 2016 [293]. MACI uses a porcine type I/III collagen membrane as a cell carrier, seeding expanded patient autologous chondrocytes onto the collagen membrane and implanting it into the cartilage defect site. The MACI clinical trial (SUMMIT study) enrolled 144 patients randomly assigned to the MACI group or microfracture group, with 24‐month follow‐up. Results showed that the MACI group had significantly superior KOOS (Knee Injury and Osteoarthritis Outcome Score) pain scores compared to the microfracture group (*P* < 0.01), and MRI assessment showed higher cartilage regeneration quality in the MACI group (MOCART score: 75.2 vs. 60.8) [294]. The success of MACI lies in: (1) using natural collagen membrane to provide a three‐dimensional microenvironment, maintaining chondrocyte phenotype; (2) complete RCT data demonstrating superiority; (3) long‐term follow‐up (5 years) showing durable efficacy and safety. MACI's predecessor, Carticel, was the first‐generation autologous chondrocyte implantation product, receiving FDA approval in 1997, using periosteal covering technique to secure cell suspension [295]. However, Carticel was gradually replaced by third‐generation products like MACI due to complications such as large periosteal harvesting trauma, uneven cell distribution, and postoperative periosteal hypertrophy and calcification [296]. The lesson from Carticel is that natural material carriers (such as collagen membranes) significantly improved cell distribution and clinical outcomes, while cell suspension alone is difficult to achieve ideal repair.

#### 7.2.3 Pure Synthetic and Natural Material Composite Products

The emergence of natural/synthetic material composite products stems from the inherent deficiencies of pure synthetic materials in osteochondral repair. Pure synthetic materials (such as PLGA, PCL, PGA), while possessing controllable mechanical properties and degradation rates, suffer from bioinertness that leads to poor cell adhesion capacity, lack of cell recognition sites (such as RGD sequences), and difficulty in inducing chondrocyte differentiation and ECM synthesis [189].

Additionally, synthetic material degradation products (such as lactic acid) may cause local acidic environments and inflammatory reactions, inhibiting tissue regeneration. In contrast, natural materials (such as collagen, hyaluronic acid, cartilage ECM) are rich in bioactive molecules and cell‐binding sites, capable of promoting cell migration, proliferation, and differentiation, but their mechanical strength is insufficient to withstand joint loading [297]. Therefore, natural/synthetic material composite products achieve functional complementarity by combining the bioactivity of natural materials with the mechanical properties of synthetic materials, making them an ideal choice for osteochondral repair [298].

However, the design of composite materials is not a simple material stacking, but requires precise matching of material components, degradation rates, and interfacial bonding. The failure case of TruFit CB (Smith & Nephew) fully illustrates this point. TruFit CB is composed of poly(lactic‐co‐glycolic acid) (PLGA), calcium sulfate, and polyglycolic acid (PGA) fibers, and received FDA approval in 2008 through the 510(k) pathway for osteochondral defect repair. The product's original design intent was to provide mechanical support through PLGA, promote osteoconduction through calcium sulfate, and enhance structural stability through PGA fibers [299]. However, clinical follow‐up showed that 6–12 months after TruFit CB implantation, some patients experienced increased joint pain, incomplete cartilage regeneration, and scaffold remnants, ultimately leading to voluntary market withdrawal in 2013 [300]. The lessons from TruFit CB's failure are: pure synthetic materials lack bioactive signals, and even through composite design, it is difficult to compensate for their biological deficiencies, and the mismatch between material degradation rate and tissue regeneration rate is a key bottleneck in composite material design.

In contrast, Maioregen (Fin‐CerAMICa) successfully achieved complementary advantages of natural/synthetic materials [298]. Maioregen employs a gradient composite structure of equine type I collagen and hydroxyapatite (HA), mimicking the stratified characteristics of the osteochondral interface: the upper layer is pure collagen (simulating the cartilage layer), the middle layer is collagen‐HA composite (simulating the calcified cartilage layer), and the lower layer has high HA content (simulating the subchondral bone layer) [301]. This gradient design enables Maioregen to possess different mechanical properties and bioactivity at different levels: the cartilage layer is rich in collagen, providing cell adhesion sites and biological signals; the bone layer is rich in HA, providing osteoconductivity and mechanical support. Maioregen obtained CE marking in 2010, and clinical studies have shown good efficacy in osteochondral defect repair: at 12 months post‐implantation, IKDC scores improved from a preoperative 45.3 to 82.7 (*P* < 0.001), with MRI showing complete cartilage and subchondral bone regeneration at the defect site, without scaffold remnants or inflammatory reactions [292]. The key to Maioregen's success lies in: (1) natural collagen provides bioactivity: collagen is rich in RGD sequences and growth factor binding sites, promoting chondrocyte adhesion, proliferation, and ECM synthesis; (2) HA provides mechanical support and osteoconductivity: HA has a slow degradation rate that matches the rate of new bone formation, avoiding premature scaffold failure; (3) gradient structure mimics the natural osteochondral interface: the material composition and properties of different layers match the physiological characteristics of cartilage, calcified cartilage, and subchondral bone, promoting stratified regeneration.

#### 7.2.4 The Critical Role of Natural Materials in Clinical Translation Products

As outlined in Section 7.2, the 47 approved products were categorized by material source and composition into five groups, namely allogeneic, non‐allogeneic, composite, natural/synthetic composite, and purely synthetic, providing the classificatory framework for the comparative analysis that follows. The following comparisons illustrate how material origin shapes biological performance and clinical outcomes across these five categories.

The comparison between Maioregen and TruFit CB clearly reveals the fundamental reason why natural/synthetic composite materials are superior to pure synthetic materials: the bioactive signals provided by natural materials (such as cell adhesion sites, growth factor binding, ECM induction) are necessary conditions for tissue regeneration, while pure synthetic materials cannot provide these signals, and relying solely on mechanical support and degradation rate control is difficult to achieve ideal repair [302]. Furthermore, the degradation products of natural materials (such as collagen peptides, hyaluronic acid oligosaccharides) typically have pro‐regenerative effects, whereas the degradation products of synthetic materials (such as lactic acid) may cause inflammation, further limiting the application of pure synthetic materials.

The 47 approved products listed in Table 6 fully demonstrate the dominant position of natural materials in osteochondral repair. Products containing natural materials account for as high as 91.5% (43/47 products), among which: allogeneic and non‐allogeneic source products (26 products, 55.3%), due to their natural biocompatibility, abundant cell recognition sites, and endogenous growth factors, have become the preferred solution for cartilage repair; composite source products (12 products, 25.5%) achieved synergistic enhancement of mechanical properties and bioactivity by combining different natural materials or natural/synthetic materials; natural/synthetic material composite products (5 products, 10.6%) further demonstrate the core role of natural materials in providing biological signals and promoting tissue regeneration. In contrast, pure synthetic material products account for only 8.5% (4/47 products), and are mainly limited to specific application scenarios such as osteoconductive scaffolds, with their bioinertness and lack of cell induction capacity limiting their application in cartilage repair.

The success experiences and failure lessons of these products (such as the market withdrawal of TruFit CB versus the success of Maioregen) clearly indicate that: the bioactive microenvironment provided by natural materials—including ECM components, cell adhesion sites, growth factor binding capacity, and tissue‐specific signals—is a key element in achieving osteochondral regeneration, and these properties cannot be replaced by pure synthetic materials. Future clinical translation of natural materials should focus on tissue specificity of material selection, standardization of decellularization processes, precise matching of degradation rates with tissue regeneration, and optimization of clinical trial design and regulatory pathways, in order to fully leverage the unique advantages of natural materials in osteochondral repair.

### 7.3 Translation Challenges From Laboratory to Clinic and Future Trends

Natural materials face the “Valley of Death” in transitioning from laboratory research to clinical application, encountering multiple challenges, including technical, regulatory, cost, and commercialization barriers, while innovations in regulatory policies and technological advances are creating new opportunities for clinical translation.

#### 7.3.1 GMP Production and Process Challenges

The FDA requires medical products to be manufactured under GMP conditions, including cleanroom environments (ISO Class 5–8), standard operating procedures (SOPs), quality control (QC), equipment validation, and personnel training [341]. While these requirements apply to all medical products, natural biomaterials present unique challenges due to their biological origin and inherent variability.

Unlike synthetic materials with defined chemical structures, natural biomaterials carry intrinsic safety risks that must be addressed during manufacturing. These include endotoxin contamination (FDA limit <20 EU/device), residual DNA/RNA fragments (<50 ng/mg dry weight threshold), pathogen transmission risks (viruses, prions), and immunogenic components such as α‐Gal epitopes in xenogeneic materials. If inadequately removed, these contaminants can trigger inflammatory responses, immune rejection, or disease transmission. Furthermore, batch‐to‐batch variability arising from donor age, tissue source, and processing conditions poses significant challenges for product standardization and reproducibility.

To address these inherent challenges, manufacturers must establish strict quality standards encompassing collagen purity, molecular weight distribution, and crosslinking degree. Rigorous validation of decellularization processes is essential to ensure the complete removal of cellular components. Additionally, sterilization methods require careful optimization to balance material performance preservation with microbial inactivation efficacy, as γ‐ray irradiation may induce collagen degradation, while terminal sterilization approaches may fail to eliminate endotoxins completely. The high costs of GMP production (cleanroom construction requires millions of dollars), extensive quality testing (endotoxin assays, DNA quantification, viral clearance studies), and long validation timelines (6‐12 months) represent major barriers for small and medium‐sized enterprises [342].

#### 7.3.2 Cost‐Effectiveness and Market Access

The commercialization of natural material products requires evaluation of cost‐effectiveness ratios and insurance coverage strategies. MACI treatment costs approximately $40 000–50 000, significantly higher than microfracture surgery ($5000–8000), but successfully obtained Medicare reimbursement by providing high‐quality RCT data and long‐term follow‐up data [343]. DeNovo NT costs approximately $15 000–20 000, with the advantage of single‐surgery completion, but due to lack of RCT data, some insurance companies refuse reimbursement [344]. Cost‐effectiveness analysis must comprehensively consider material costs, R&D costs (clinical trials, FDA approval), production costs (GMP facilities, quality control), market size, and insurance reimbursement policies. Market access strategies include early communication with payers, conducting real‐world evidence (RWE) studies to supplement RCT data, and conducting health economics research to demonstrate cost‐effectiveness advantages.

#### 7.3.3 Intellectual Property Protection

Intellectual property protection is the core of commercialization. Patent strategies include material composition patents (such as the chemical structures of GelMA, Col II MA), manufacturing process patents (such as decellularization processes, crosslinking methods), application patents (specific indications), and equipment patents (3D printers, bioreactors). Patent protection for natural materials faces challenges: natural materials themselves are not patentable and must obtain protection through chemical modification or specific processes; the contradiction between patent term (20 years) and product development cycle (10‐15 years) results in short patent protection periods after market launch; patent infringement is difficult to prove. Patent portfolio planning must be conducted in the early stages of R&D, covering multiple dimensions such as materials, processes, and applications, to form a patent portfolio.

#### 7.3.4 Regulatory Innovation and Accelerated Approval

The FDA has established multiple accelerated approval pathways: Fast Track for products treating serious diseases and meeting unmet medical needs; Breakthrough Therapy Designation for products significantly superior to existing therapies, providing intensive guidance; and Regenerative Medicine Advanced Therapy (RMAT) designation for regenerative medicine products, offering early interaction, priority review, and accelerated approval. MACI shortened approval time through RMAT designation, and more natural material products may accelerate market entry through the RMAT pathway in the future.

Real‐world evidence (RWE) is data collected from real clinical practice (electronic medical records, patient registries, insurance databases), used to supplement RCT data [345]. The FDA released the RWE framework in 2018, encouraging the use of RWE to support regulatory decisions. The advantages of RWE include large sample sizes, strong representativeness, reflection of real clinical practice, and lower costs than RCTs [346]. Patient registries represent a particularly valuable tool for natural material products, enabling systematic collection of long‐term outcomes data that can support regulatory approval and reimbursement decisions. Additionally, international regulatory harmonization through IMDRF and ISO standards (ISO 10993, ISO 13485) is facilitating global market access for natural biomaterial products.

## 8 Conclusion and Future Perspectives

The prospects of biologically derived natural materials in the field of osteochondral repair should not be underestimated. We believe that in the coming years, particularly when biomaterial sources shift from autologous human tissues or fresh xenogeneic tissues to decellularized matrices, renewable natural polymers, or waste biological resources (such as shells, silk, etc.), this field will achieve tremendous scientific and technological progress. The selection of biomaterials should be based on their compositional characteristics, structural‐functional compatibility, and relevance to osteochondral tissue regeneration mechanisms, rather than solely relying on material availability or geographical origin.

However, despite the promising prospects, this field still faces a critical challenge: many newly developed biomaterials remain confined to laboratory research and scientific publications, unable to transition into clinical practice or industrial application. To bridge this translational gap, it is essential to establish reliable and verifiable correlations between biomaterial sources, processing techniques, compositional characteristics, and osteochondral repair outcomes.

The preparation and evaluation of biomaterials must prioritize “standardization” and “reproducibility.” The successful development of osteochondral graft substitutes, collagen‐based products, and hyaluronic acid formulations has demonstrated that establishing rigorous quality control systems—such as ISO 10993 biocompatibility standards, defined molecular weight specifications, and the standardized Type I/III collagen ratios employed by Chondro‐Gide—can transform biological raw materials with significant batch‐to‐batch variations into medical products with consistent performance. While new standards such as ASTM F2451 and ISO/TC 194 are refining specific requirements for osteochondral materials, establishing a complete standardized pathway from laboratory to clinic still requires sustained efforts. Only when researchers and clinicians reach consensus on standardized processing and evaluation systems can we ensure that biologically derived materials continue to advance along a scientifically rigorous trajectory.

## Author Contributions

**Hengyu Liu**: Conceptualization, Literature search, Writing – original draft, Writing – review & editing. **Hanyang Zhang**: Data collection. **Wenbo Yang**: Literature search, Data collection. **Hao Chen**: Literature search, Data collection. **Jincheng Wang**: Conceptualization, Supervision, Writing – review & editing, Project administration. **Fei Chang**: Conceptualization, Supervision, Writing – review & editing, Funding acquisition.

## Funding

This work was supported by the National Natural Science Foundation of China (No. 82572761 and No. 824B2121), and the Scientific Research Project of the Jilin Provincial Department of Education (No. JJKH20262164BS).

## Consent

All authors involved in the writing of this review have provided their consent for its publication.

## Conflicts of Interest

The authors declare that they have no known competing financial interests or personal relationships that could have influenced the content of this review.

## Data Availability

Data sharing not applicable to this article as no datasets were generated or analysed during the current study.
